# TGF-beta coordinates changes in Keratin gene expression during complex tissue regeneration

**DOI:** 10.1038/s41598-025-22590-2

**Published:** 2025-11-04

**Authors:** Dipak D. Meshram, Yuchang Liu, Molly Worth, Changqing Zhang, Tom J. Carney, Henry H. Roehl

**Affiliations:** 1 School of Bioscience, The University of Sheffield, Firth Court, Western Bank, Sheffield, S10 2TN UK; 2 Lee Kong Chian School of Medicine, Nanyang Technological University, Singapore, Singapore

## Abstract

Zebrafish larval tail regeneration can be broadly broken down into three phases: wound repair, initiation of regeneration and redevelopment of the tail. We and others have shown that activation of the Hedgehog and TGF-beta signalling pathways is required to trigger tail regeneration. In this study we initially focus on these two pathways to better understand their role regeneration. We have found that Hedgehog signalling acts upstream of the TGF-beta pathway by activating transcription of the TGF-beta ligand *tgfb1a*. We have identified differentially expressed genes that are regulated after tail excision as well as those that are dependent upon TGF-beta signalling. From this analysis we found that Keratin genes fall into two categories of co-expression: one set that is upregulated by TGF-beta signalling after injury (*krt*_*up*_) and a second set that is downregulated (*krt*_*down*_). We have mapped the expression of a representative member of *krt*_*up*_* (krt97*) and *krt*_*down*_ (*krt5*) sets during development and regeneration and show that *krt97* is found in epithelia that is moving and *krt5* is expressed in epithelia that is stationary. To see whether these two sets show co-expression more broadly, we analysed single cell RNA sequencing data and found that *krt*s also fall into the same categories during zebrafish development. Based upon our results we propose that the *krt*_*up*_ set may allow for collective epithelial migration while the *krt*_*down*_ set may inhibit mobility. These results reveal one mechanism by which TGF-beta may initiate regeneration and could help to explain how changes in cytoskeletal composition influence cell movement during development and repair.

**Supplementary Information:**

The online version contains supplementary material available at 10.1038/s41598-025-22590-2.

## Introduction

Humans are able to heal wounds and regrow many tissues such as skin and muscle however the regeneration of complex structures is limited. Aquatic vertebrates on the other hand, are able to regenerate large portions of organs including limb, tail, spinal cord, retina and heart^1^. When comparable damage occurs in humans, tissue usually fails to regrow and scarring occurs. One possible explanation for this poor regenerative potential may be a failure to recruit competent cells to the site of injury in sufficient numbers to restore the missing tissue. Thus, the characterisation of signalling pathways that are able to induce regenerative cell migration in model organisms may aid in the development of novel regenerative medicine strategies.

**Fig. 1:**
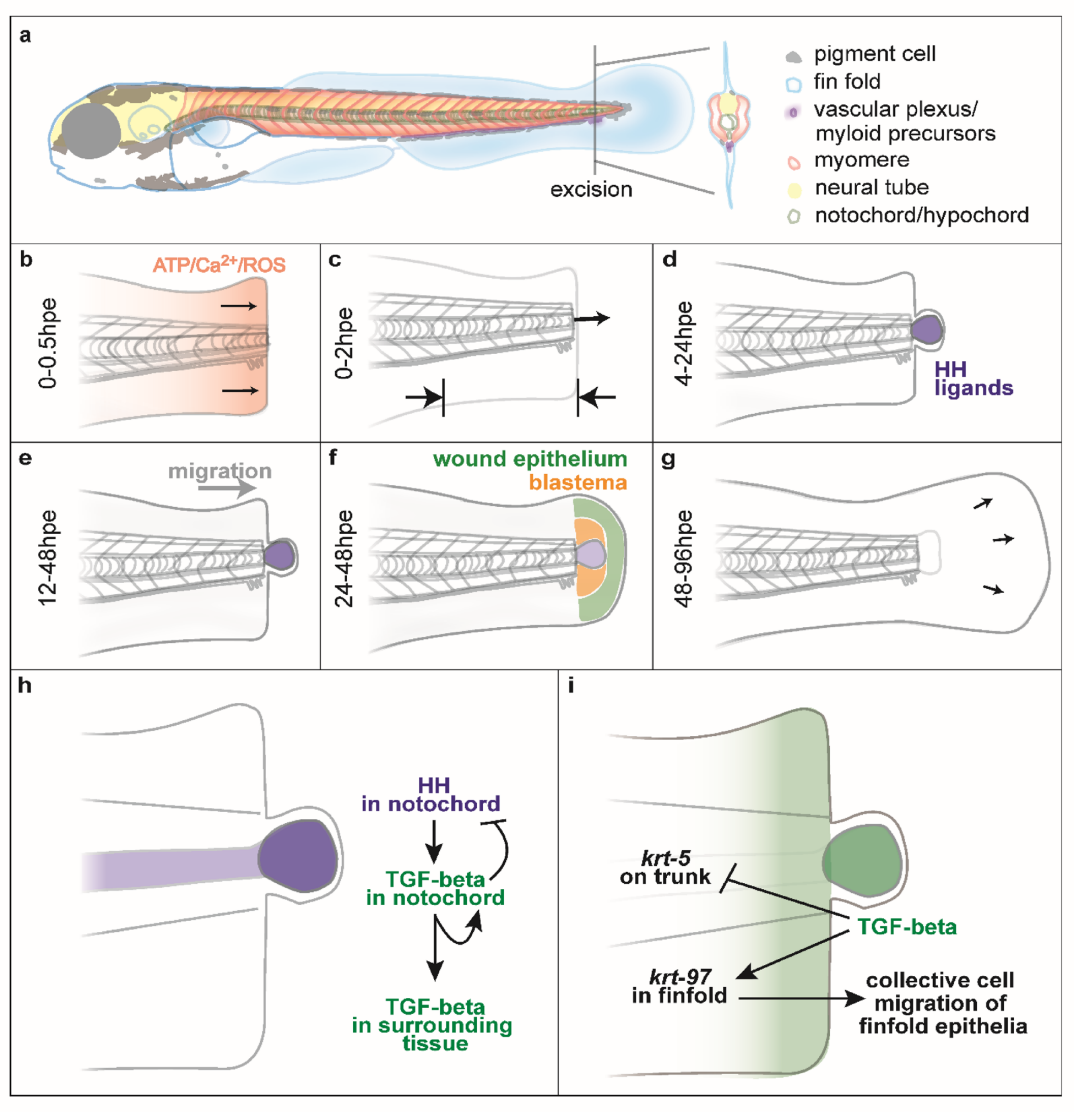
Signalling events during tail regeneration. (**a**) Larval tail excision removes the end of the zebrafish tail and includes different cell types. (**b**) Within 0.5 h post excision (hpe) early wound signals are released and the wound is closed. (**c**) The body axis shortens and the notochord bead is extruded. (**d**) The notochord bead becomes a HH signalling centre that activates regeneration. (**e**) Regenerative cells migrate towards the stump. (**f**) The wound epithelium and blastema form. (**g**) The tail reforms. (**h** and **i**) Models presented in this paper.

Zebrafish larval tail excision offers a rapid and simple model to understand the basic principles of regeneration (Fig. 1a)^2,3^. Excision is typically performed between 48 and 72 h post fertilisation (hpf) and involves removal of some of the larval fin (finfold), muscle, neural tube, notochord and other tissue. Following excision, early wound signals (ATP, Calcium and ROS) are released and the wound rapidly closes (Fig. 1b)^4^. The body axis shortens and a ball of notochord cells forms at the stump (called the notochord bead) (Fig. 1c,d). The notochord bead expresses Hedgehog (HH) ligands which induce migration of regenerative cells to the stump (Fig. 1d,e). This results in the formation of the blastema and wound epithelium (Fig. 1f). The blastema and wound epithelium express signalling pathways which orchestrate the redevelopment of the tail (Fig. 1g). Regeneration of the tail completes by around 72 h post excision (hpe).

The TGF-beta pathway plays crucial roles in wound healing in mammalian systems and is involved with wound closure, fibroblast activation, inflammation and fibrosis^5^. It has also been shown to act during the regeneration of different zebrafish tissues including heart, spinal cord, lateral line and adult skin^6–9^. Recent analysis has shown that TGF-beta signalling acts during larval tail regeneration and is linked closely with pro-regenerative metabolic reprogramming^10^. How the TGF-beta pathway relates to the existing signalling network for tail regeneration (Fig. 1a–g) is not clear.

Keratins (Krts) are intermediate filament proteins that are found mainly in the cytoskeleton of epithelial cells and are associated with desmosomes and hemidesmosomes^11^. They are divided into type I and type II which assemble into heterodimers through their conserved, central α-helical rod domains. These type I/type II heterodimers then further polymerise into filaments which elongate and branch to form a network that fills the cell. They give strength to many structures including skin, hair and nails. In addition to their structural roles they have effects on diverse cellular processes such as cell migration, signalling, metabolism and mechanotransduction^12,13^. Studies in zebrafish have primarily used *krts* as markers of epithelia and transgenic fish carrying fluorescent proteins driven by *krt* promoters are widely used^14,15^.

In this study we propose that the TGF-beta pathway plays a central role during tail regeneration. We find that it is activated by notochord-derived HH signalling through the upregulation of transcription of the ligand *tgfb1a* in the notochord bead (Fig. 1h). Notochordal TGF-beta signalling then inhibits HH signalling by repressing transcription of *indian hedgehog a* (*ihha*) in the notochord and further activates *tgfb1a* in the notochord and surrounding tissue (Fig. 1i). To better understand the downstream effects of TGF-beta signalling we use bulk RNA sequencing (RNA-seq) to identify differentially expressed genes (DEGs) after tail excision and after pharmacological inhibition of TGF-beta signalling. We identify members of the *krt* gene family as potential targets of the TGF-beta pathway after excision, and find that they fall into two categories of co-expression (synexpression): one set that is upregulated by TGF-beta signalling after injury (*krt*_*up*_) and a second set that is downregulated by TGF-beta signalling after injury (*krt*_*down*_). Next we focus on two *krt* genes, *krt97* and *krt5* as representative members of *krt*_*up*_ and *krt*_*down*_ to examine their expression during development and regeneration. We find that whereas *krt97* is expressed in epithelia that are actively moving, *krt5* is expressed in epithelium that is stationary. Using developmental single cell RNA sequencing (scRNA-seq) datasets we show that genes in *krt*_*up*_ and *krt*_*down*_ tend not to be co-expressed suggesting that these two groups have distinct functions during development. Collectively these data suggest a model where TGF-beta allows for collective cell movement in the epithelia by modulating expression of different members of the *krt* gene family.

## Results

### TGF-beta signalling acts downstream of HH signalling

**Fig. 2:**
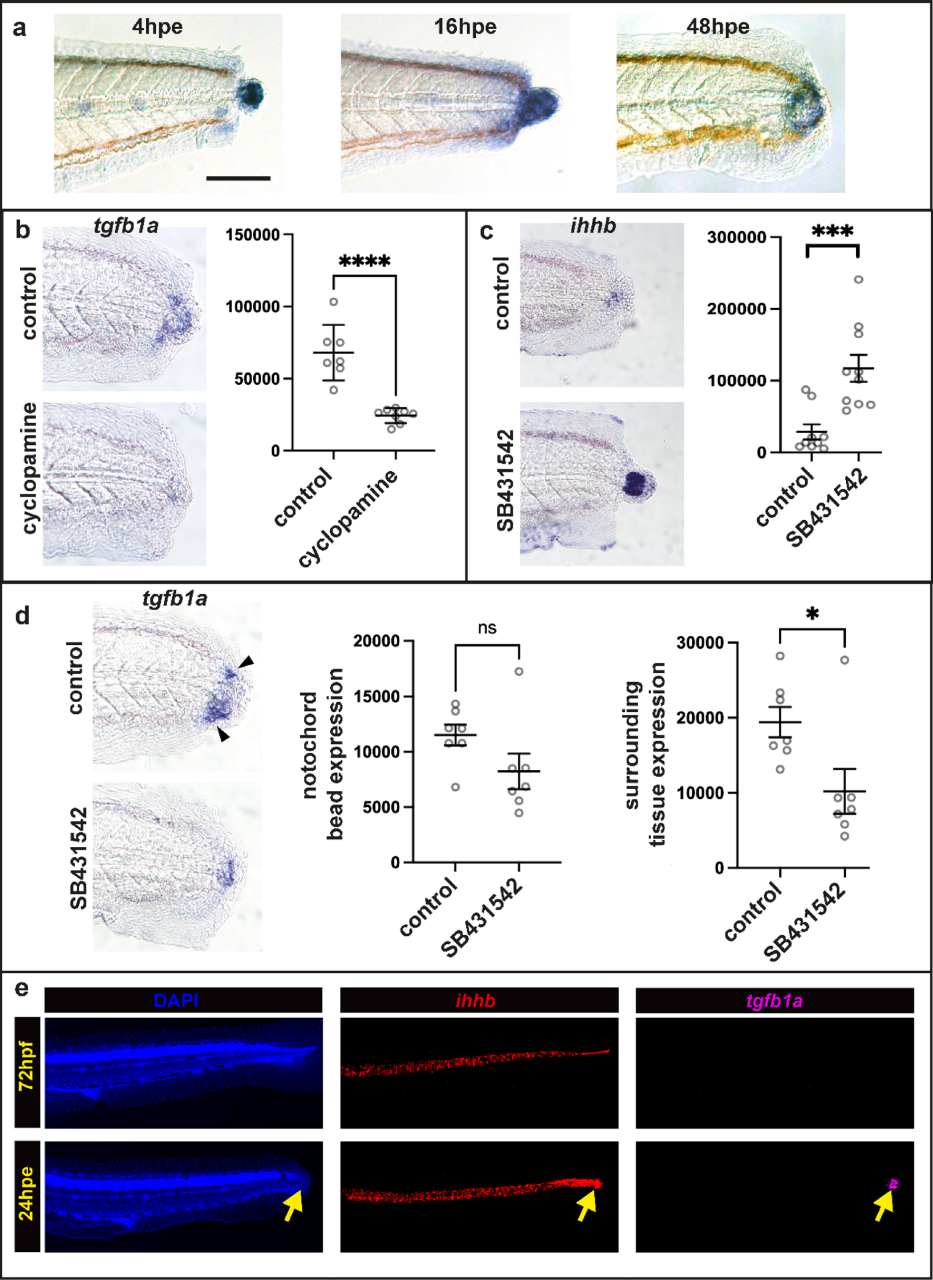
*tgfb1a* expression is activated by Hedgehog signalling in the notochord bead. (**a**) Expression of *tgfb1a* at three time points after tail excision. Expression is initially confined to the notochord, but spreads to surrounding tissue over time. Scale bar 100um (**b**) Cyclopamine treatment from -2 to 24hpe results in a significant reduction in *tgfb1a* expression. (**c**) SB431542 treatment from -2 to 24hpe results in a significant increase in *ihhb* expression. (**d**) SB431542 treatment from 4 to 24hpe results in a significant decrease in *tgfb1a* expression in the tissue that surrounds the notochord bead (arrow heads). (**e**) HCR in situs of an uninjured fish at 72hpf (top row) and injured fish at 24hpe (bottom row). In uninjured fish *ihhb* is expressed throughout the notochord prior to excision and *tgfb1a* is absent (10/10 fish). After excision, *tgfb1a* expression is activated (13/13 fish) only in the notochord bead (yellow arrows). Units for the graphs in b-d are thresholded pixels (see methods for details). Asterisks indicate significance (* = 0.05; ** = 0.01; *** = 0.001; **** = 0.0001). SB431542 concentration was 50uM and cyclopamine concentration was 20uM.

Pharmacological inhibition of the HH and TGF-beta signalling pathways have striking similarities: In both cases the wound closes and the notochord bead forms, but there is no recruitment of regenerative cells to the stump^3,10^. We have previously shown that HH ligands *ihhb* and *shha* are expressed in the notochord both before and after tail excision^3^. To begin to understand the relationship between the HH and TGF-beta pathways we used standard RNA in situ methods^16^ to identify *tgfb1a* as a candidate ligand gene that is expressed in the notochord bead immediately after wounding (Fig. 2a). Expression is initially just in the notochord and then spreads to the surrounding tissue. To determine whether the two pathways regulate each other we treated fish with either a HH pathway inhibitor (cyclopamine) or a TGF-beta pathway inhibitor (SB431542). We found that cyclopamine treatment blocks *tgfb1a* transcriptional activation (Fig. 2b) and SB431542 treatment increases *ihha* expression (Fig. 2c). These two findings suggest that TGF-beta signalling is likely to act downstream of the HH pathway and forms a negative feedback loop to modulate HH activity. In addition we found that TGF-beta activates its own transcription especially in the tissue surrounding the notochord bead (Fig. 2d). We have previously found that while the standard whole-mount RNA in situ methods detect expression in the notochord bead, it does not detect genes expressed in the intact notochord^3^. To investigate whether *tgfb1a* is expressed in notochord cells prior to injury, we switched to the hybridization chain reaction fluorescent (HCR) in situ method which utilises much smaller probes^17^. Whereas, *ihhb* is expressed through-out the notochord before and after injury (as shown in^3^), *tgfb1a* is only activated in the notochord bead upon injury (Fig. 2e). Together these findings suggest a model whereby the formation of the notochord bead causes the induction of *tgfb1a* expression by HH signalling^3^.

### Transcriptional analysis of TGF-beta signalling during regeneration

**Fig. 3:**
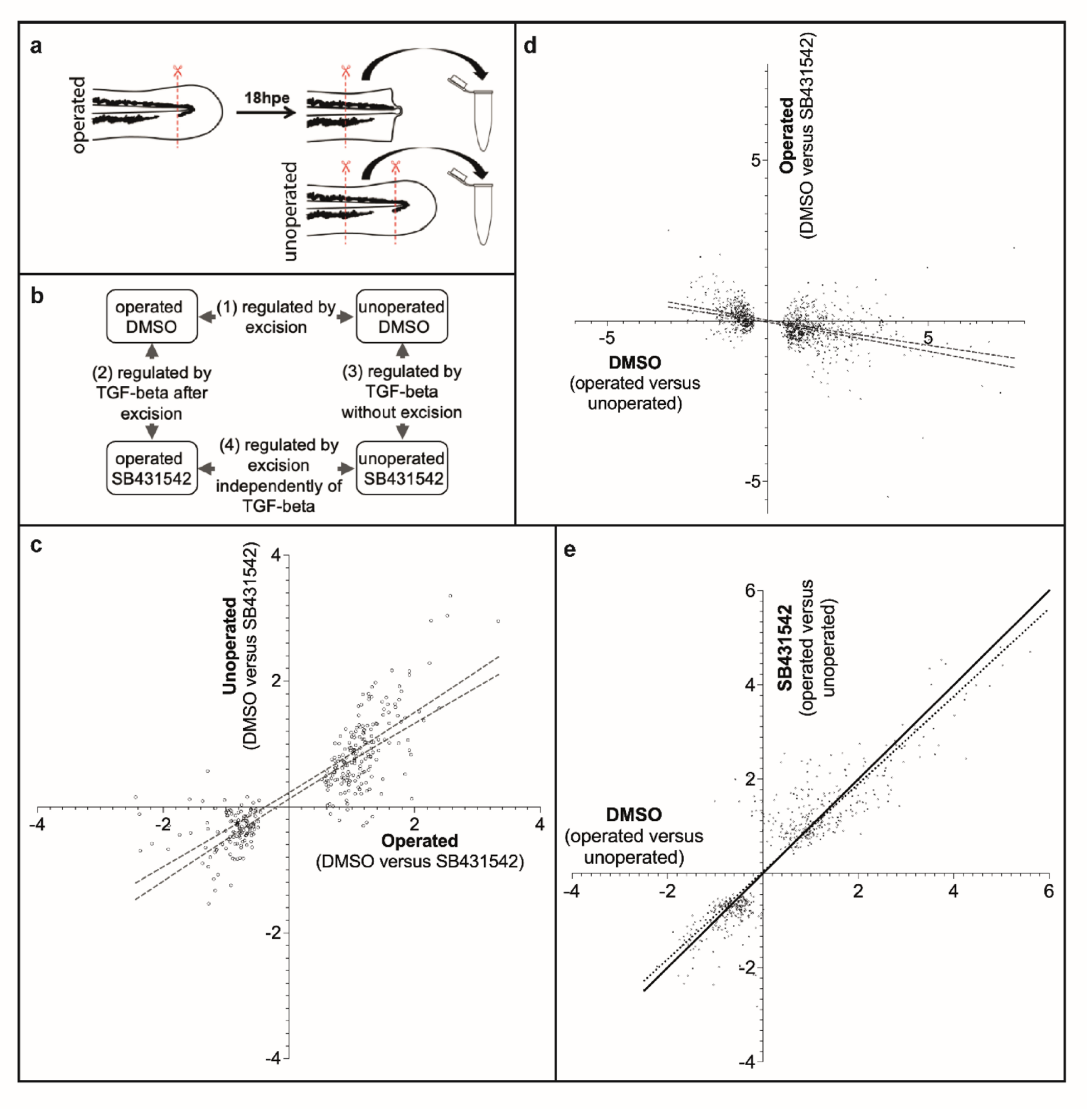
Transcriptome changes due to injury and TGF-beta activation. (**a**) Preparation of samples. Operated samples have approximately 200um of posterior tail tissue removed and are incubated until 18hpe. A second 200um tissue is then removed and processed for sequencing. The unoperated samples take a similar region of the tail for processing. (**b**) Four-way comparisons are made between the samples. (**c**) Comparison of potential TGF-beta pathway targets after injury to potential TGF-beta pathway targets without injury. The X-axis is SB431542 operated compared to DMSO operated (2 in panel b) and the Y-axis is SB431542 unoperated compared to DMSO unoperated (3 in panel b). Only DEGs that have an adjusted P-value (Padj) value < 0.05 in the SB431542 operated compared to DMSO operated set are plotted. DEGs in the upper right quadrant are genes that are potentially suppressed by TGF-beta signalling in both operated and unoperated samples. DEGs in the lower left quadrant are those that are potentially activated by TGF-beta signalling in both operated and unoperated samples. The lines indicate the regression with 95% confidence. The regression slope is 0.62 indicating that regulation is stronger in the injured dataset. (**d**) Comparison of potential DEGs after injury to potential TGF-beta pathway targets after injury. The X-axis is DMSO operated compared to unoperated (1 in panel b) and the Y-axis is SB431542 operated compared to DMSO operated (2 in panel b). Only DEGs that have a Padj value < 0.05 in the operated compared to unoperated set are plotted. DEGs in the upper left quadrant are genes that are potentially suppressed by TGF-beta signalling following injury. DEGs in the lower right quadrant are those that are potentially activated by TGF-beta signalling after injury. The lines indicate the regression with 95% confidence. (**e**) Comparison of potential targets after injury to potential TGF-beta pathway targets after injury. The X-axis is DMSO operated compared to unoperated (1 in panel b) and the Y-axis is SB431542 operated compared to SB431542 unoperated (4 in panel b). Only DEGs that have a Padj value < 0.05 in the SB431542 operated compared to SB431542 unoperated set are plotted. The solid line represents a slope of one, and the dotted line represents the linear regression. Values in (c-e) are expressed in Log2.

To begin to understand the role of TGF-beta signalling in regenerative cell recruitment, we performed bulk RNA-seq to identify DEGs after tail excision (Fig. 3a). To focus on genes that are regulated at the start of regeneration, fish were processed at 18 h post excision (hpe). RNA was collected using four treatments: DMSO (vehicle control) unoperated, DMSO operated, SB431542 unoperated and SB431542 operated (Fig. 3b). One conclusion that can be drawn from this analysis is that DEGs that are found in the SB431542 operated versus DMSO operated comparisons tend to also be DEGs in the SB431542 unoperated versus DMSO unoperated set (Fig. 3c). This may suggest that genes that are targets of TGF-beta signalling during regeneration are also responding to TGF-beta activity in uninjured fish. This is unexpected as treatment with SB431542 in unoperated fish confers no visible changes (data not shown). There are however some DEGs that are regulated by SB431542 in operated fish that do not show significant regulation by SB431542 in unoperated fish. These genes are potential wound/regeneration specific targets of the TGF-beta pathway (see Supplemental Fig. 1).

A second conclusion from the RNA-seq comparisons is that genes that are regulated during regeneration are usually influenced by SB431542 treatment (Fig. 3d). For example, DEGs that are upregulated following injury are likely to also be downregulated when injured fish are treated with SB431542. This supports the model that TGF-beta is pro-regenerative and is required during the onset of regeneration to recruit cells. However, there are some DEGs that do not fit this model (see upper right and lower left quadrants in Fig. 3d). For example, DEGs in the upper right quadrant of Fig. 3d are upregulated following injury and are upregulated when injured fish are treated with SB431542. It is not immediately clear why this is, but one possible explanation is that there is negative feedback affecting these genes and that testing at earlier time points would find that they are initially downregulated by TGF-beta. The most significant DEGs that are identified by this comparison are presented in Supplemental Fig. 2.

Further evidence that TGF-beta has a pro-regenerative role comes from comparison of DEGs after injury in DMSO to DEGs after injury in fish treated with SB431542 (Fig. 3e). In this graph, DEGs that are unaffected by the presence of SB431542 after injury fall on a slope of 1. The regression slope is closer to 0.9 indicating that there is a bias towards DEGs that are injury specific and are responding to TGF-beta. For example, genes that fall below the slope of 1 line in the upper right quadrant are predicted to be activated by TGF-beta whilst those that fall above the slope of 1 line in the lower left quadrant are predicted to be repressed by TGF-beta during regeneration. A subset of these genes are presented in Supplemental Fig. 3.

To find genes that are injury-specific but not potential TGF-beta targets we can take DEGs that fall close to the slope of 1 in Fig. 3e. We can also identify this type of gene by selecting DEGs that lie close to the X-axis in Fig. 3d. By applying both criteria we identified a subset of DEGs that are potential TGF-beta independent regeneration genes (Supplemental Fig. 4).

### Members of the Keratin family show TGF-beta-dependent responses to injury

**Fig. 4:**
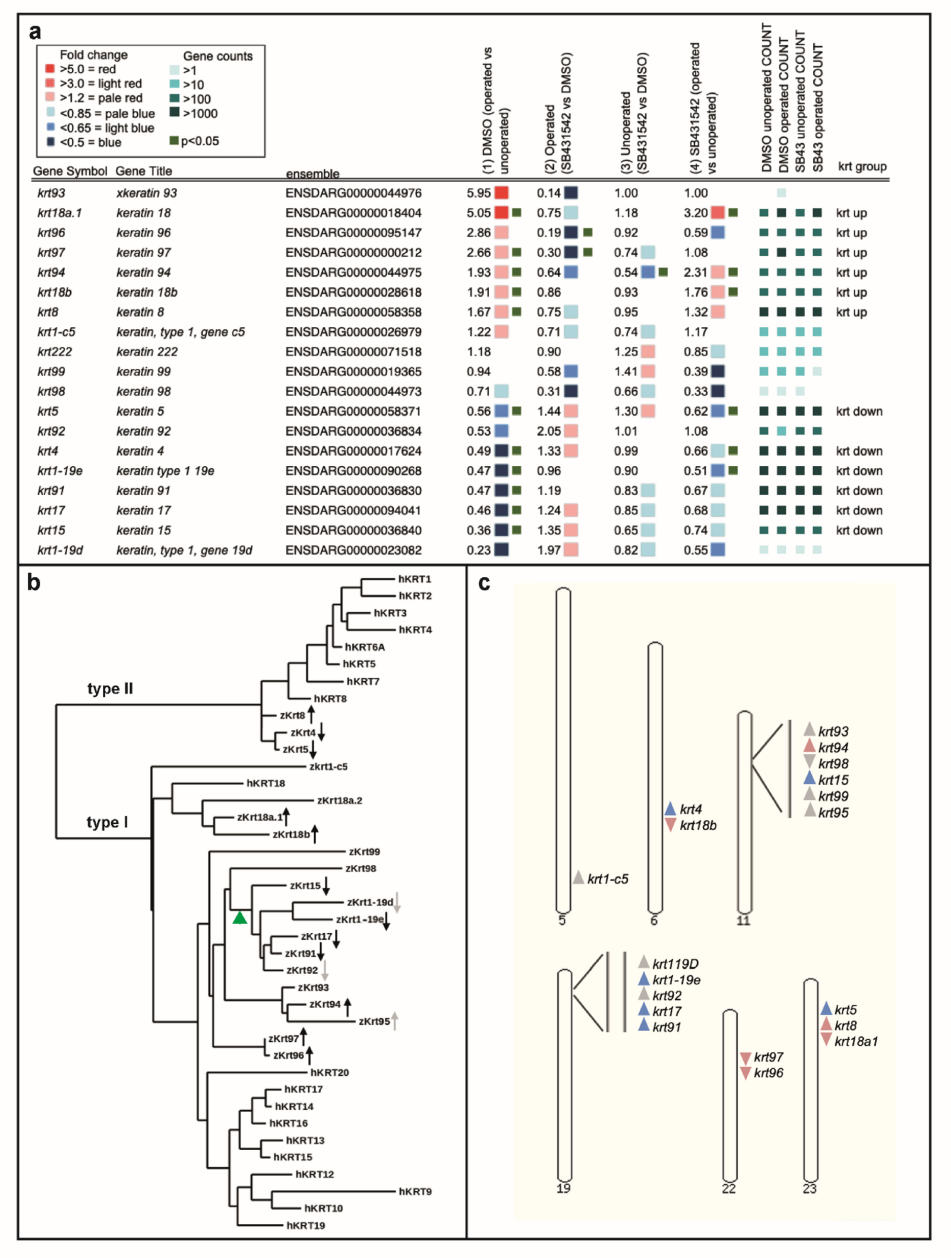
Zebrafish *krt* gene regulation and relatedness. (**a**) A list with zebrafish *krt* genes, their ensemble identifiers, the fold level of change in all four comparisons, the adjusted counts for each sample and their allocation into *krt*_*up*_ or *krt*_*down*_ groups. (**b**) Molecular phylogeny dendrogram with zebrafish (zKrt) and human (hKRT) representatives of the Keratin family. The arrows indicate upregulation or downregulation as shown in panel a. Black arrows indicate significance (*P* < 0.05) and grey arrows indicate a trend that was not significant (*P* < 0.05). Green arrowhead indicates the possible Krt_off_ branch point. Phylogram tree and alignments were done with “one click” settings www.phylogeny.fr^18^ (**c**) The genomic locations of the zebrafish *krts* with an arrowhead showing their chromosomal orientation and colour showing upregulation (pink) or downregulation (blue) by TGF-beta. The genome assembly for this analysis was GRCz11 (www.ensemble.org).

To learn more about coordinated gene expression during regeneration we searched for families of genes in the RNA-seq data (Supplemental Fig. 5). Of these, the Krt family stands out because most members are regulated by TGF-beta and these members are either activated during regeneration (*krt*_*up*_) or inhibited (*krt*_*down*_) (Fig. 4a). We grouped the *krts* as being in these synexpression groups based upon being significant (*P* < 0.05) in at least one of the comparisons. There are nine *krts* that do not fall into these groupings. Two of these, *krt18a.2* and *krt95*, were not identified in our analysis. Of the remaining seven, six are expressed at low levels at the time of our assay which perhaps affects our detection, and the seventh (*krt222*) is only distantly related to other members of the *krt* family.

The zebrafish *krt* family is smaller than the mammalian family and is less well studied^19,20^. To begin to understand the significance of these two groupings, we first performed phylogenetic analysis to see whether members of Krt_up_ or Krt_down_ are more closely related to each other based upon their predicted protein sequence (Fig. 4b). We have included members of the human Krt (hKRT) family in this analysis for comparison and to enable clear separation of type I and type II Keratins. There are three type II Krts with one Krt_up_ and two Krt_down_ members. These fall next to the human type II Krts in one cluster. The type I branch has greater variation with one of the human Krts (hKRT18) showing more similarity to zebrafish genes than to other human Krts. Within the type I branch there is some correlation between expression and relatedness. Krt_up_ and Krt_down_ appear to separate at some point between Krt15 and Krt94 (green arrowhead in Fig. 4b). Intriguingly, the members that show regulation but have a P value that is greater than 0.5 do still show a correlation in their relatedness. For example, although *krt1-c5* does not show significance in pairwise comparisons, it does show the expected changes in expression for Krt_up_ genes (Fig. 4a) and falls into the Krt_up_ phylogenetic branch. Likewise, *krt1-19d* has the expected changes in expression for Krt_down_ and falls into the Krt_down_ branch. The correlation in the type I group between expression and relatedness may indicate that there are structural differences between these Krt_up_ and Krt_down_ members and that the two groups were formed by one evolutionary event.

To see whether this correlation extends to their chromosomal organisation, we looked at the genomic organisation of *krts* (Fig. 4c). *krts* are found clustered on six chromosomes. The cluster on chromosome 19 contains up to three members of *krt*_*down*_ and the cluster on chromosome 22 has two members of *krt*_*up*_. Three of the other chromosomes are mixed synexpression genes and chromosome 5 has a single *krt* gene. Overall the genomic organisation does not show a strong correlation with the type of synexpression.

### *krts* have distinct domains of expression during regeneration and development

**Fig. 5:**
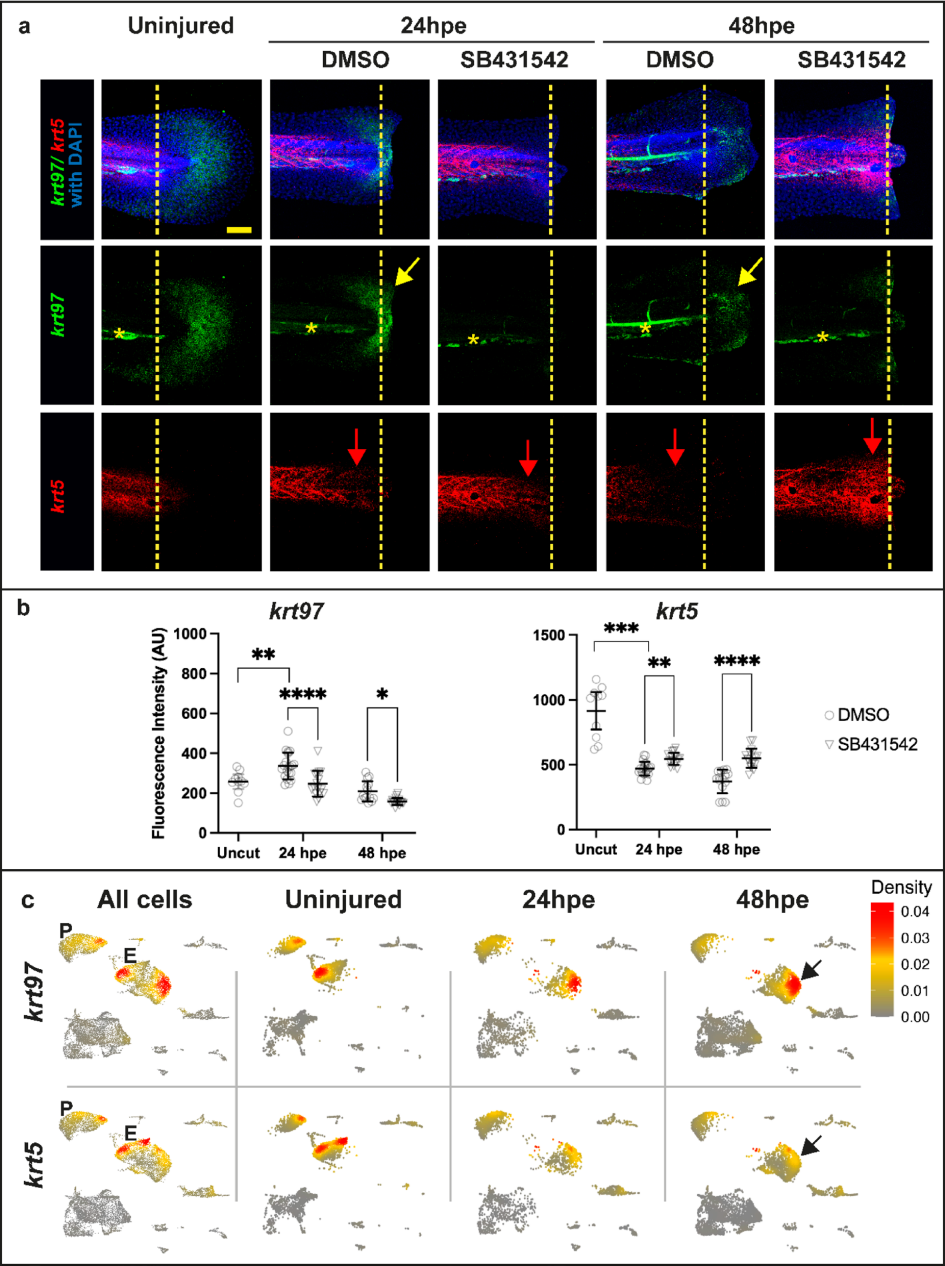
*krt97* and *krt5* are inversely regulated during tail regeneration. (**a**) In situ analysis of *krt97* and *krt5* in uninjured and injured fish treated with SB431542. Larvae were treated with DMSO or 50 M SB431542 for 2 h prior to tail excision and kept in the chemical solution until 24 or 48 hpe. Yellow arrows indicate the re-expression of *krt97* in regenerating epithelia. Red arrows indicate the reduction of *krt5* expression on the trunk of control injured fish and the increase in expression in injured fish treated with SB431542. Yellow dotted lines indicate the excision plane before and after tail excision. Yellow stars indicate non-specific fluorescence in the vasculature. See Supplemental Fig. 6 for orthogonal views. Scale bar is 50um (**b**) Quantification of fluorescent in situs as shown in (**a**). Asterisks indicate significance as in Fig. 2. (**c**) Expression of *krt97* and *krt5* in scRNA-seq cell clusters representing periderm (P) and epidermis (E) during regeneration. Arrows in the 48hpe timepoint indicate epidermal cells that are expressing *krt97* and not *krt5*.

To understand the relevance of *krt* regulation at the tissue level we decided to do HCR in situ analysis. For this we have focused on *krt5* as a representative of *krt*_*down*_ and *krt97* as a representative of *krt*_*up*_. We first tested the response of these at three stages, uninjured (96hpf), 24hpe(96hpf) and 48hpe(120hpf) (Fig. 5). In fish at these stages we found that *krt97* and *krt5* are expressed in distinct domains. *krt97* is primarily in epithelial cells of the finfold in a crescent shape around the end of the tail (Fig. 5a). This region of expression is removed following tail excision. *krt5* on the other hand is found in the epithelia that sits on the trunk. At 24hpe epithelial cells have gathered on the end of the stump to form the wound epithelium. At this time, *krt97* expression is upregulated in wound epithelium and *krt5* expression is reduced in the trunk epithelia nearest to the stump. By 48hpe the fin fold is reforming and *krt97* is expressed in the new area of growth. On the other hand *krt5* is strongly downregulated near to the regrowing tail. Inhibition of TGF-beta with SB431542 during regeneration results in a strong increase in *krt5* and a reduction in *krt97* consistent with the RNA-seq data. To further confirm our results, we probed scRNA-seq data for fish at 24hpe and 48hpe^10^. We see that *krt97*^+^ and *krt5*^+^ cells are in distinct domains with *krt97*^+^ cells increasing after excision and *krt5*^+^ cells decreasing (Fig. 5c). Pseudo bulk RNA-seq analysis of this scRNA-seq data suggests an increase of 1.5 fold for *krt97* at 24hpe and a decrease of *krt5* expression to 0.5 at 24hpe and 0.38 at 48hpe. Together these data suggest that *krts* respond to injury in a TGF-beta dependent fashion and are expressed in distinct compartments of the skin.

**Fig. 6:**
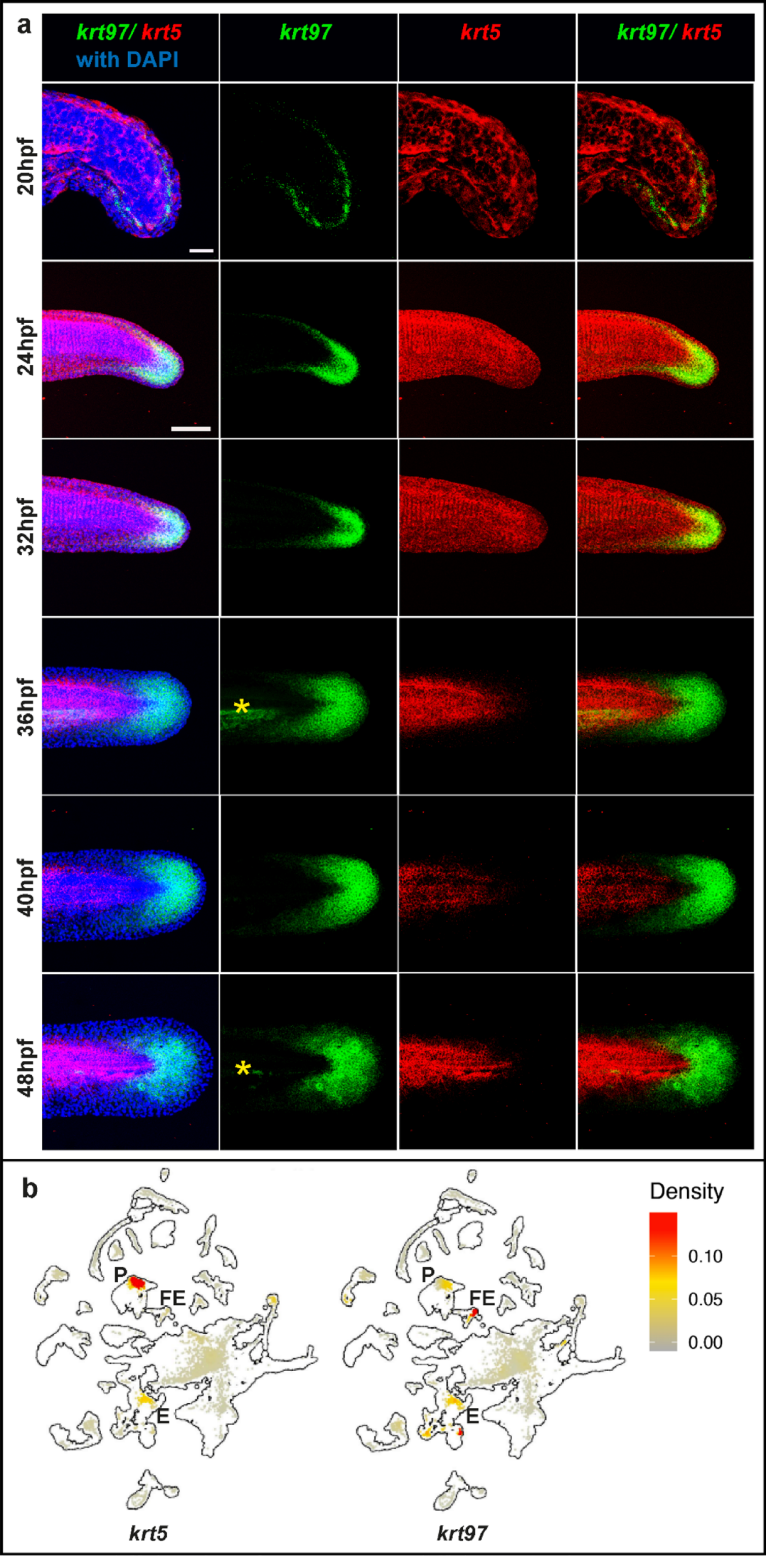
Dynamic expression of *krt97* and *krt5* during finfold development. (**a**) HCR in situ analysis of *krt97* and *krt5* between 20 and 48hpf. Yellow stars indicate non-specific fluorescence in the vasculature. Scale bar for 20hpf is 50um and 100um for 24hpf to 48hpf. (**b**) Expression of *krt97* and *krt5* in scRNA-seq cell clusters. The outline shows all cells in the dataset (3hpf to 120hpf) Cells in the 36-46hpf data set are shown in grey, yellow or red according to their level of expression. Clusters representing periderm (P), fin epithelium (FE) and Epithelium (E) are labelled. Data taken from Daniocell scRNA-seq dataset (Farrell et al. 2018; Sur et al. 2023).

Given that regeneration of the fin fold is likely to recapitulate aspects of fin fold development, we decided to look at tail developmental stages to compare development to regeneration. The fin fold emerges between 20 and 48hpf (Fig. 6a). At 20hpf it can be seen as a ridge of epidermal cells that sits on the midline of the left and right sides of the tail. At this stage *krt5* is in scattered cells throughout the tail epidermis and at 24hpf and 32hpf *krt5* is expressed throughout the tail epidermis. Between 36 and 48hpf the fin fold goes through morphogenesis and *krt5* expression is limited to the trunk of the tail. At 20hpf *krt97* expression is in a layer of cells at the base of the epidermal ridge in a crescent of cells around the end of the tail. This expression expands and as the finfold expands it becomes more restricted to the caudal end of the finfold. Intriguingly *krt97* is not expressed in the most distal layer of cells of the finfold. To summarise, *krt5* and *krt97* are expressed in different areas of the tail epidermis with *krt97* being expressed in the fin fold as it undergoes morphogenesis.

To further understand the compartmentalisation of *krt5* and *krt97* expression we analysed the Daniocell scRNA-seq dataset which contains cells from 3 to 120hpf^21,22^. We found that *krt97* and *krt5* show separate domains of expression primarily between 30 and 58hpf and at other time points the expression domains are not distinct (data not shown). Figure 6b shows pooled cells from 36 to 46hpf and *krt5* is expressed in a cluster annotated as periderm and *krt97* is in fin epithelium and epithelium clusters.

**Fig. 7:**
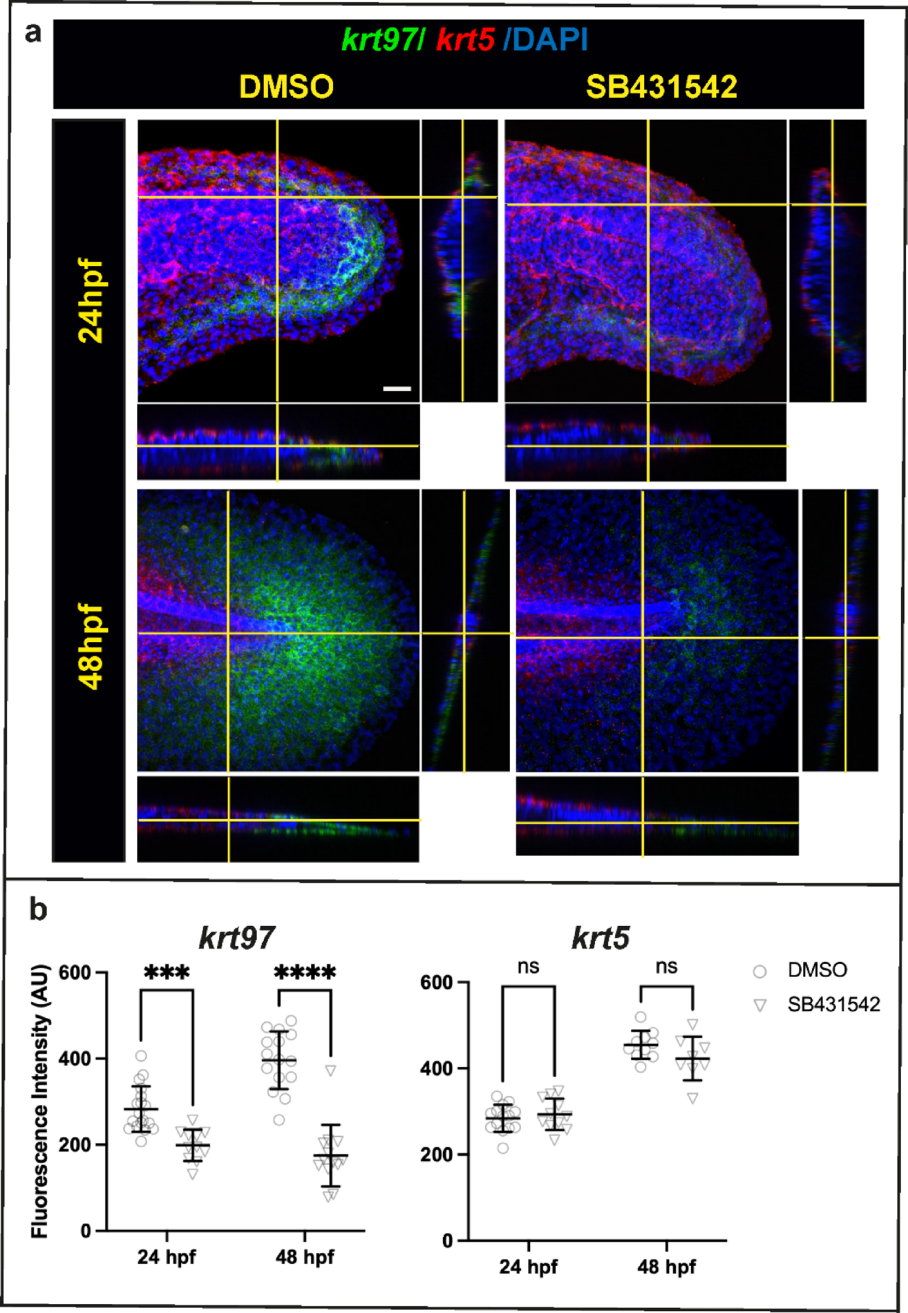
Inhibition of TGF-beta signalling affects *krt97* expression during development. (**a**) HCR in situ staining for *krt97* and *krt5* with and without SB43154. Orthogonal views are shown to the right and below each image. The planes used for the orthogonal views are indicated by the yellow lines. Scale bar is 20um. (**b**) Quantification of fluorescence in (**a**). Asterisks indicate significance as in Fig. 2 resulting from the unpaired t test. For the 24hpf timepoint, embryos at the 15 somite stages (16.5hpf) were treated with DMSO or 100 uM SB431542 continuously. For the 48hpf stage, larvae at 40hpf were treated with DMSO or 100 uM SB431542.

Treatment with the inhibitor SB431542 during finfold development does not confer a morphological phenotype suggesting that TGF-beta signalling does not strongly affect finfold development (data not shown). However, considering our result that most genes which are regulated by TGF-beta during regeneration also show some regulation in uninjured fish, we decided to see whether SB431542 affects *krt* gene expression during development. We found that treatment between 16.5 to 24hpf and 40 to 48hpf results in a strong reduction in *krt97* expression (Fig. 7). On the other hand, *krt5* did not show a change in expression. This suggests that the regulation of *krts* during development may be related to that seen during regeneration, at least for *krt97*.

**Fig. 8:**
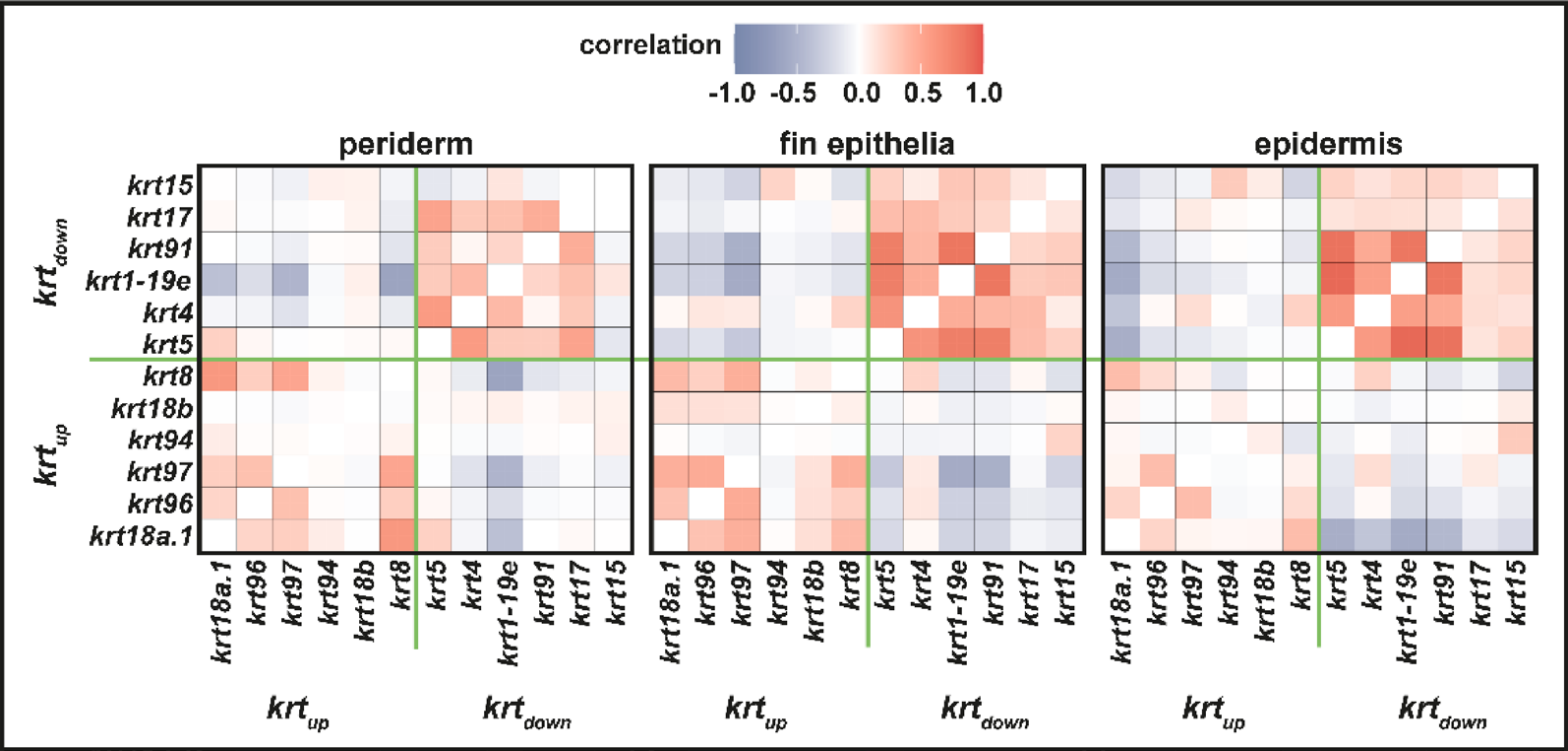
Co-expression of *krts* in skin clusters. scRNA-seq data from the Daniocell analysis (Farrell et al. 2018; Sur et al. 2023) was subsetted based upon periderm, fin epithelium and epidermis identifiers using all available timepoints. Each of these subsets was then tested for correlation (using Pearson Correlation in Seurat) for the *krt*_*down*_ and *krt*_*up*_ genes. Red colour indicates a positive correlation and blue colour indicates a negative correlation.

This compartmentalised expression of *krt5* and *krt97* during finfold development and regeneration raises the possibility that other members of *krt*_*up*_ and *krt*_*down*_ show segregated expression patterns during development. We have tested other *krts* and find that there is limited overlap between *krt*_*up*_ and *krt*_*down*_ gene expression (Supplemental Fig. 7 and data not shown). To see whether this is the case more broadly we pooled periderm, fin epithelium and epithelium clusters from the Daniocell scRNA-seq dataset and assessed the degree of co-expression between *krt*_*up*_ and *krt*_*down*_ using Pearson’s correlation coefficient analysis (Fig. 8). This data shows that there is a strong degree of coexpression within each group. For example, *krt1-19e* expression is correlated to other members of *krt*_*down*_ especially in the epidermis with *krt5* and *krt91* showing the highest association with *krt1-19e*. *krt1-19e* shows a negative correlation to members of *krt*_*up*_ particularly with *krt18a.1*, *krt97* and *krt8* in the periderm. This data shows the co-regulation of *krts* seen during regeneration and with manipulation of TGF-beta signalling is also found more broadly in epithelia during developmental stages.

**Fig. 9:**
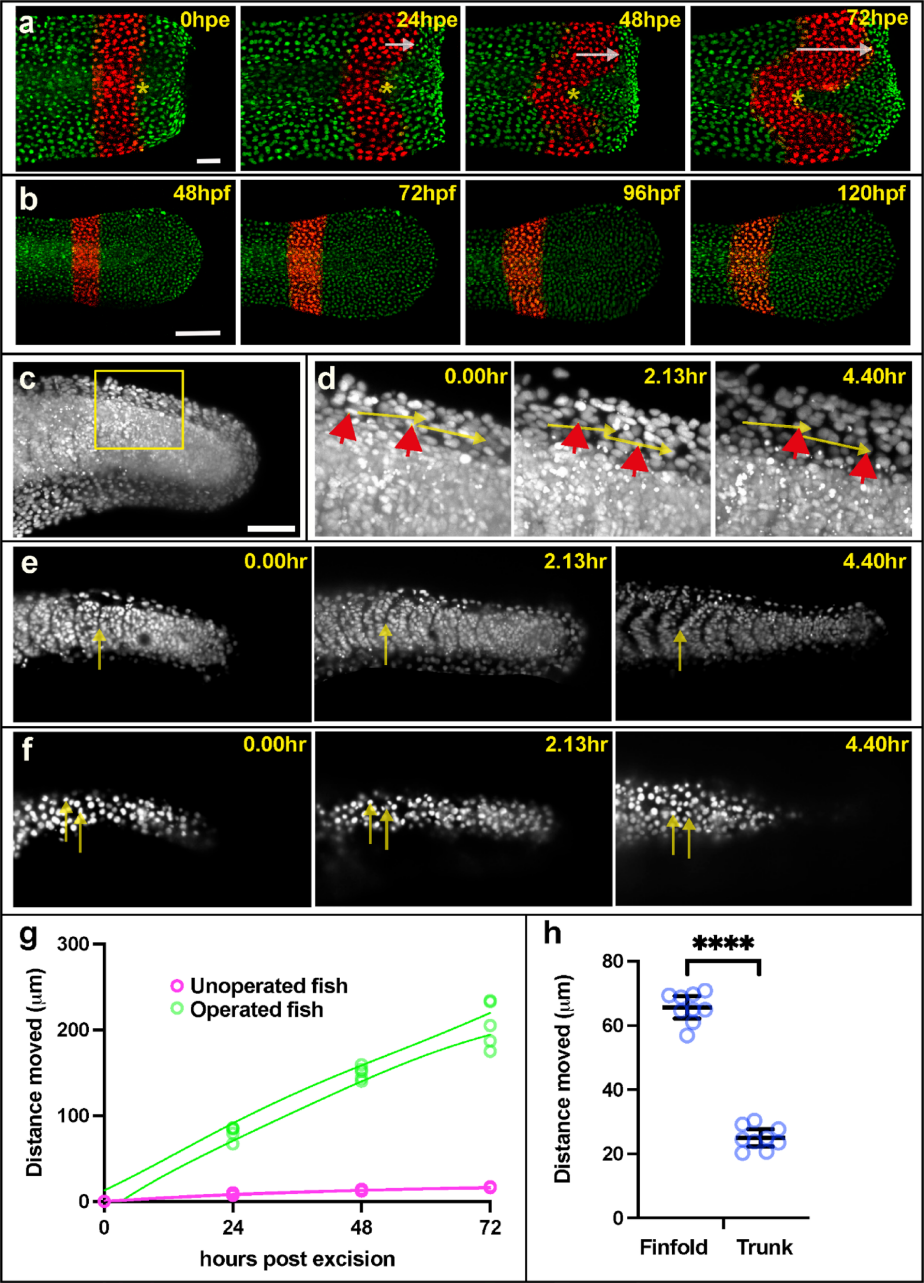
Collective migration of epithelial cells is faster in the finfold than on the trunk. (**a**) Cell nuclei were followed over a period of three days after tail excision using Dendra photoconversion (red nuclei). The asterisks mark the border of converted nuclei on the trunk and the arrows indicate the distance travelled by nuclei on the fin epithelia relative to the trunk. Scale bar = 50um (**b**) Unoperated control fish show very little movement over these four days. Scale bar = 100um (**c**) Overview image (z-projection) showing nuclei labelled red fluorescence* (actb2:h2b-mCherry)* at 24hpf. Scale bar = 100um. (**d**) Close-up of the box in (**c**) showing three time points during a time-lapse recording. The red arrowheads point to two finfold epithelium nuclei at the different time points. The yellow arrows indicate the distance travelled. (**e**) Sections showing somite boundary positions at three timepoints with arrows marking the same position. (**f**) Sections following epithelium nuclei on the trunk at three timepoints with arrows marking their positions. (**g**) Quantification of movement of finfold cells after excision as in panels (**a**) and (**b**). Lines indicate non-linear fit 95% confidence interval. Individual unpaired t test for each timepoint are < 0.0001. (**h**) Quantification of movement of finfold cells during development as in panels (**c**–**f**). Note that the midline somite boundary is used to standardise the epithelial cell movements (arrows in **e**). Asterisks indicate significance as in Fig. 2 resulting from the unpaired t test.

The expression *krt97* during finfold regeneration and development correlates to regions that are undergoing morphogenesis. These epithelia need to engage in collective cell migration and are likely to alter their cytoskeleton to allow this to occur. On the other hand, *krt5* is reduced during finfold morphogenesis and regeneration suggesting that it is inhibitory to epithelial movement. To investigate this hypothesis, we decided to analyse epithelial cell movement during development and regeneration to see whether different epithelial regions have different mobility. Our predictions were that regions that express *krt97* (epithelia of the fin fold) move more quickly than regions that express *krt5* (epithelia on the trunk). To test cell movement during regeneration we used photo-conversion of a nuclear localised dendra transgene (Fig. 9a, b, g and Supplemental Fig. 8). We found that cells in the proximal finfold move the furthest averaging approximately 70um per day relative to cells on the trunk. In unoperated animals there was very little movement. To assess cell movement during development, we made timelapse recordings of a nuclear-localised fluorescent reporter and measured movement relative to the underlying somites (Movie 1). As during regeneration we found that during development cells on the finfold move faster than cells on the trunk (by approximately 15um/hour) (Fig. 9c–f, h). These results show that epithelia move at dramatically different rates during regeneration and development, and that the differences in movement may correlate to differences in *krt* expression.

## Discussion

In this study we focus on how regenerative cells are recruited to the site of injury after tail excision. We present a model whereby HH signalling activates regenerative cell recruitment by transcriptionally activating *tgfb1a* expression in the notochord (Fig. 1h). The TGF-beta pathway then induces more expression of *tgfb1a* whilst inhibiting expression of *ihhb* in the notochord. After establishing that TGF-beta signalling acts downstream of the HH pathway, we then focus on the identification of genes regulated by TGF-beta during regeneration to investigate how cell recruitment takes place. These data reveal sets of genes that are important in the regeneration and indicate that TGF-beta plays a central role in this process. We propose that members of the *krt* gene family as potential effectors of regenerative TGF-beta signalling and that TGF-beta signalling enables epithelial collective cell migration by modulating the expression of different *krts* (Fig. 1i).

TGF-beta has been linked to wound healing by mammalian studies^23^. The initial activation of the pathway during wounding is by serum protease activity which releases TGF-beta1 from platelets. The activated pathway then has many roles including keratinocyte recruitment. Our results suggest that during tail regeneration the role in keratinocyte recruitment is similar, but the initial activation of the TGF-beta pathway involves transcriptional activation of *tgfb1a*. Another similarity is that there is also some evidence for TGF-beta regulation of mammalian *Krts* during wounding. For example, one study has found that *Krt15* is repressed during wound healing and is also repressed by TGF-beta signalling suggesting that it would be in the *krt*_*off*_ synexpression group^24^. Furthermore, links between *Krts* and TGF-beta have been characterised for different tumour types and *Krts* are used as biomarkers for cancer prognosis^25,26^. Our study does not rule out additional roles for TGF-beta such as in activating proliferation^7^ and changing the composition of the extracellular matrix^7,27^ as is seen in zebrafish heart regeneration.

Mammalian studies that primarily use scratch assays have suggested that Krt’s usually have negative roles on cell movement, but can also increase migration^13,28–30^. For example, Keratin 6 and 16 are activated after wounding and are thought to play a role in retraction of intermediate filaments from the cell periphery^31^ and cause a reduction in cell adhesion^32,33^. In addition, Krts may affect cell migration by altering cellular elasticity^30^ and myosin activation^34^. In Xenopus keratins are involved with adhesion and cell contraction during gastrulation^35^. Understanding the precise molecular role of zebrafish Krts during collective epithelial cell migration will take further investigation.

Regulated expression of *krts* during regeneration has been observed in adult zebrafish tail regeneration^36^, Xenopus larval tail regeneration^37^ and newt limbs^38^. This suggests that dynamic expression of *krts* is conserved during organ regeneration. Our finding that *krts* have similar regional expression domains during development and regeneration may indicate that embryonic expression patterns are re-established during regeneration. The significance of regionalised expression domains of *krts* during development has yet to be explored.

*Movie 1 Description.* The movie shows the animal imaged in Fig. 9c–f. Panel A shows the maximum intensity projection with yellow arrows following two finfold epithelium nuclei. Panel B shows sections through the somites with a somite boundary position marked with an arrow. Panel C shows superficial sections of the trunk epithelium with arrows following two nuclei.

## Methods

### General methods

Experimental procedures and fish maintenance were performed using standard methods. All methods were approved by the University of Sheffield and performed in accordance with the relevant guidelines and regulations. This study is reported in accordance with ARRIVE guidelines. All animal husbandry and experimentation was carried out under the supervision and approval of the University of Sheffield Ethics Board. Fish lines were sourced from and maintained by the University of Sheffield Biological Services Aquarium under Home Office project licence 3,627,554. Adult zebrafish were maintained with a 14 h light/10 h dark cycle at 28 °C according to standard protocols and were mated using pair mating in individual cross tanks. Fish were euthanised in 40 μg/ml Tricaine (3-amino benzoic acidethylester) in E3. For more information on how individual experiments were performed, please refer to figure legends and the sections below.

### Bulk RNA-seq analysis

Zebrafish embryos were raised until 72hpf. A scalpel was used to remove the end of the tail from anaesthetised larvae using the pigment gap as a reference^3^. The four conditions were: Unoperated DMSO, Operated DMSO, Unoperated SB431542 and Operated SB431542. There were 3 replicates for each condition and these each contained 100 tails pieces. Each replicate was processed in 1 ml of TRI Reagent (Merck) using a plastic pestle to dissociate the tissue. Following the TRI Reagent procedure, the RNA was further purified by LiCL precipitation. The RNA quality was checked by Qubit (ThermoFisher) and TapeStation (Agilent) protocols. The libraries were made using the Lexogen QuantSeq 3’ FWD library kit and using NextSeq500 (Illumina) to generate approximately 5 million 75SE reads for each library. The resulting sequence data was demultiplexed using the BlueBee tool (Illumina), aligned to the ensemble zebrafish reference genome and analysed in DESeq2 in R^17,39^. Plots were generated using Prism 10 (Graphpad). Data sets available at GEO Series GSE296469.

### RNA in situ hybridization and quantification

Standard RNA in situ hybridisation was done using the Thisse protocol^16^. HCR in situ hybridization was performed using zebrafish whole mount protocol^17^ and reagents purchased from Molecular Instruments (Los Angeles, California). To report in situ expression patterns, a representative animal is shown in the figure panel. For quantification, measurements were made in imageJ and graphs were generated and analysed in Prism 10 software. All images were blinded before quantification. Error bars indicate the 95% confidence interval and the centre bar represents the mean. Individual circles represent individual animals tested.

Figure 2b–d were quantified using the colour threshold method. A rectangle selection tool was used to select the region of interest (ROI) covering the end of the tail, and the images were cropped to the ROI. The same ROI was applied to each image within an experiment. The colour threshold function was used to set the same hue, saturation and brightness which was used for every image from the same experiment. The area above the set threshold was selected and the pixel number was counted. The p values were generated using an unpaired t-test and are as follows: 2b < 0.0001; 2c 0.0010; 2d (bead) 0.1013 and (surrounding) 0.0253.

Figures 5b and 7b were quantified by the corrected total fluorescence method. An ROI was made using the rectangle tool and this was used to measure fluorescence on the sample and off the sample (background measurement). The size of the ROI was the same for all the compared images. The corrected fluorescence intensity was calculated by subtracting the background reading from the sample fluorescence.

For Fig. 5b the number for uncut = 10, 24hpe = 20, 48hpe = 16. The statistical test used is 2 way ANOVA with Fisher’s multiple comparison. The p values are as follows: *krt97* (Uncut vs 24hpe DMSO) 0.0045; *krt97* (24hpe DMSO vs SB431542) < 0.0001; *krt97* (48hpe DMSO vs SB431542) 0.0105; *krt5* (Uncut vs 24hpe DMSO) 0.0001; *krt5* (24hpe DMSO vs SB431542) 0.0013; *krt5* (48hpe DMSO vs SB431542) < 0.0001.

For Fig. 7b the numbers for *krt97* expression, 24 hpf DMSO n = 18; SB431542 n = 11; 48hpf DMSO n = 15; SB431542 n = 13. for *krt5* expression, 24hpf DMSO n = 14; SB431542 n = 11; 48hpf DMSO n = 10; SB431542 n = 8. The statistical test used is 2 way ANOVA with Sidak’s multiple comparison test. The p values are as follows: *krt97* (24hpf DMSO vs SB431542) 0.0010; *krt97* (48hpf DMSO vs SB431542) < 0.0001; *krt5* (24hpf DMSO vs SB431542) 0.8089; *krt5* (48hpf DMSO vs SB431542) 0.1533.

### PhotoConversion

*Ubi:h2b-dendra*^40^ transgenic fish were used for photoconversion experiments. Larvae at 48hpf were placed in a holding chamber in a glass bottom dish. Rectangular ROI was used to draw the region on the tail and photoconverted exposing to 405 nm with 100% laser power for 2 min. Unoperated and Operated fish were monitored over a period of time, and images were captured at 543 nm and 488 nm wavelength using Nikon A1 confocal microscope. For quantification cells at the centre of the trunk were used as a reference point, and the maximum distance travelled by fin fold cells was measured. The analysis was done on six operated animals and three unoperated animals.

### Time-lapse

*actb2:h2b-mCherry* transgenic fish were used for these experiments. Zebrafish larvae were anaesthetised and imaged by light sheet microscopy (Bruker TrueLive3D) for 280 min. For quantification, three fish were analysed, (two started at 22hpf and one at 24hpf). Three epithelial cell nuclei were measured in the proximal fin fold and three epithelial cell nuclei in the centre of the trunk. The boundary between two somites was used as a reference point to standardise movement of the epithelial cells.

### scRNA-seq analysis

Data sets GSE145497 and GSE223922 were downloaded from the NCBI/Geo server. Analysis was done using Seurat version 5.1.0, R (version 4.4.1 2024–06-14) and R Studio (version 2024.04.2 + 764) and is displayed in the Uniform Manifold Approximation and Projection (UMAP) format. A complete list of installed R packages and versions is available in Supplemental Table 1.

For Fig. 5c, raw data from GSE145497 was read into Seurat as H5 files, merged and QC filtered for nUMI > 500, nGene > 250, Novelty > 0.8 and mito.ratio < 0.2. PCA and UMAP were run on the merged dataset and gene expression was visualised by kernel density estimation using the Nebulosa function^41^.

For Fig. 6b, expression matrix data from GSE223922 was read into Seurat processed as above. The UMAP of total cells was used to generate an outline for the full set. The data was then subsetted to include only 36-46hpf using the stage.group identifier included in the dataset. Gene densities for 36-46hpf are shown using the Nebulosa function. The tissue figure identifier was used to identify periderm, fin epithelia and epithelia.

For Fig. 8, expression matrix data from GSE223922 was read into Seurat processed as above. The tissue figure identifier was used to make subsets for periderm, fin epithelia and epithelia. These were then plotted using the pearson parametric correlation test in Seurat.

## Supplementary Information

Supplementary Information 1.

Supplementary Information 2.

## Data Availability

Data sets from our RNAseq experiment are available from GEO Series GSE296469.
