# Supplementary material for: TGF-beta coordinates changes in Keratin gene expression during complex tissue regeneration
Source: Sci Rep. 2025 Nov 4;15:38645. doi: 10.1038/s41598-025-22590-2 (PMC12586446; doi:10.1038/s41598-025-22590-2)
Supplement: Supplementary file 1 — Supplementary Information 1. [file 41598_2025_22590_MOESM1_ESM.pdf]

Supplemental Data

Supplemental Figures 1-8

Fold change
- >5.0 = red
- >3.0 = light red
- >1.2 = pale red
- <0.85 = pale blue
- <0.65 = light blue
- <0.5 = blue

Gene counts
- >1
- >10
- >100
- >1000

p<0.05 p<0.1

Gene Symbol	Gene Title	ensemble	operated vs unoperated DMSO	operated vs unoperated SB43	SB43 vs DMSO operated	SB43 vs DMSO unoperated	DMSO unoperated COUNT	DMSO operated COUNT	SB43 unoperated COUNT	SB43 operated COUNT
lama3	laminin subunit alpha-3	ENSDARG00000022615	7.41	1.23	0.18	1.11				
manf	mesencephalic astrocyte-derived neurotrophic	ENSDARG00000063177	3.22	1.87	0.59	1.02				
tram1	translocation associated membrane protein 1	ENSDARG00000019137	2.99	1.36	0.49	1.08				
efemp2a	EGF containing fibulin-like extracellular matrix	ENSDARG00000094324	2.92	1.98	0.61	0.90				
krt96	keratin 96	ENSDARG00000095147	2.86	0.59	0.19	0.92				
si:dkey	protein coding gene	ENSDARG00000090552	2.83	1.82	0.67	1.05				
hpcal4	hippocalcin like 4	ENSDARG00000070491	2.75	0.85	0.33	1.09				
slc39a7	solute carrier family 39 (zinc transporter),	ENSDARG00000104451	2.46	1.53	0.59	0.95				
si:dkey	protein coding gene	ENSDARG00000096678	2.13	0.77	0.39	1.07				
BX511082.1	protein coding gene	ENSDARG00000113678	1.82	0.99	0.52	0.97				
serpinh1a	serpin peptidase inhibitor, clade H (heat shock	ENSDARG00000075954	1.79	1.00	0.51	0.91				
arhgdig	Rho GDP dissociation inhibitor (GDI) gamma	ENSDARG00000004034	1.75	1.17	0.72	1.07				
cpm	carboxypeptidase M	ENSDARG00000011769	1.66	1.01	0.56	0.91				
sox4b	SRY (sex determining region Y)-box 4b	ENSDARG00000098834	1.56	0.82	0.51	0.96				
sec23a	Sec23 homolog A, COPII coat complex	ENSDARG00000104230	1.56	1.09	0.65	0.94				
virma	si:ch211-79l20.4	ENSDARG00000075824	1.50	0.92	0.57	0.94				
dlx5a	distal-less homeobox 5a	ENSDARG00000042296	1.47	0.81	0.53	0.96				
bhlha9	basic helix-loop-helix family, member a9	ENSDARG00000103981	1.47	0.77	0.50	0.95				
psat1	phosphoserine aminotransferase 1	ENSDARG00000016733	1.41	0.75	0.50	0.94				
cct5	chaperonin containing TCP1, subunit 5	ENSDARG00000045399	1.40	0.97	0.63	0.91				
acot7		ENSDARG00000018653	1.39	0.77	0.51	0.92				
psmd6	proteasome 26S subunit, non-ATPase 6	ENSDARG00000070674	1.37	0.94	0.62	0.90				
fstl1b	follistatin-like 1b	ENSDARG00000039576	1.28	0.87	0.66	0.97				
ppp1r3b	protein phosphatase 1, regulatory subunit 3B	ENSDARG00000044691	1.27	0.57	0.44	0.98				
gps1	G protein pathway suppressor 1	ENSDARG00000040650	1.26	0.98	0.73	0.94				
ccdc6a	coiled-coil domain containing 6a	ENSDARG00000043334	1.21	0.87	0.70	0.97				
zgc:92744	zgc:92744	ENSDARG00000076223	1.06	0.76	0.69	0.96				
immt	inner membrane protein, mitochondrial	ENSDARG00000102874	1.03	0.79	0.69	0.90				
ACADSB		ENSDARG00000098174	1.03	0.71	0.61	0.88				
btr12	bloodthirsty-related gene family, member 12	ENSDARG00000051809	0.97	3.43	3.88	1.10				
mfn2	mitofusin 2	ENSDARG00000079504	0.94	0.53	0.63	1.11				
tmem131	transmembrane protein 131	ENSDARG00000056259	0.93	1.40	1.53	1.02				
got1	glutamic-oxaloacetic transaminase 1, soluble	ENSDARG00000039093	0.85	1.46	1.58	0.93				
galnt6	UDP-N-acetyl-alpha-D-galactosamine:	ENSDARG00000014386	0.73	2.05	2.51	0.90				
rfx2	regulatory factor X, 2 (influences HLA class II	ENSDARG00000013575	0.72	1.33	1.91	1.03				
pmp22a	peripheral myelin protein 22a	ENSDARG00000105223	0.58	0.88	1.57	1.03				
matn3b		ENSDARG00000069265	0.57	0.39	0.66	0.97				
evpla	envoplakin a	ENSDARG00000019808	0.53	1.04	1.91	0.97				
fh	fumarate hydratase	ENSDARG00000075132	0.52	0.84	1.63	1.01				
lipg	lipase, endothelial	ENSDARG00000031044	0.42	0.74	1.89	1.07				

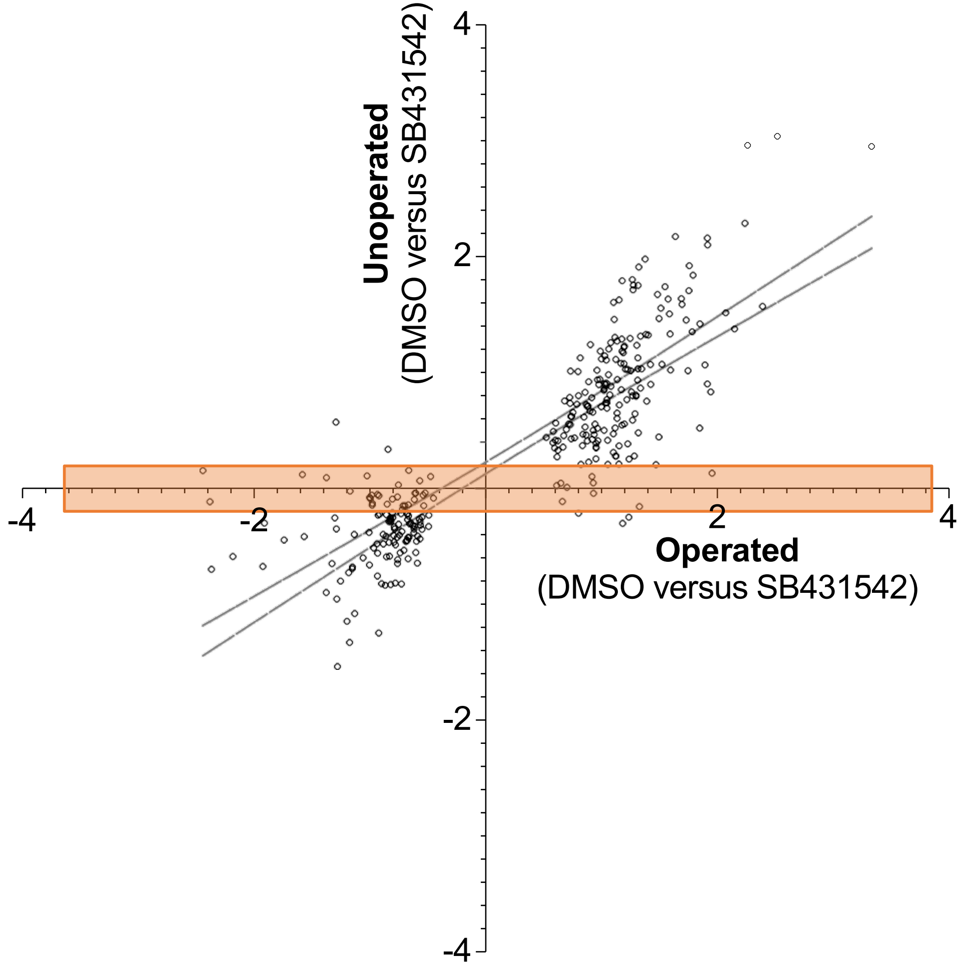

Supplemental Figure 1. Potential injury-specific TGF-beta target DEGs. DEGs with SB431542 operated versus DMSO operated (with P_{adj} value < 0.05) and a lack of differential expression in SB431542 unoperated versus DMSO unoperated are presented in this table. The columns show the ensemble identifiers, the fold level of change and the adjusted counts for each sample. These are the DEGs that lie in the boxed area of the graph (as in Fig 3c) and have a Log_2 score of between 0.2 and -0.2 in SB431542 unoperated versus DMSO unoperated comparison (i.e. genes that are between 0.87 and 1.15 fold change). Note that the graph is in Log_2 scale and the list shows fold change.

Fold change
>5.0 = red
>3.0 = light red
>1.2 = pale red
<0.85 = pale blue
<0.65 = light blue
<0.5 = blue

Gene counts
>1
>10
>100
>1000

p<0.05 p<0.1

Gene Symbol	Gene Title	ensemble	operated vs unoperated DMSO	operated vs unoperated SB43	SB43 vs DMSO operated	SB43 vs DMSO unoperated	DMSO unoperated COUNT	DMSO operated COUNT	SB43 unoperated COUNT	SB43 operated COUNT
mmp9	matrix metallopeptidase 9	ENSDARG00000042816	10.35	7.44	2.42	3.37				
lama3	laminin subunit alpha-3	ENSDARG00000022615	7.41	1.23	0.18	1.11				
ctss2.1	cathepsin Sb, tandem duplicate 1	ENSDARG00000074656	6.91	6.61	2.06	2.15				
f13a1b	coagulation factor XIII, A1 polypeptide b	ENSDARG00000036893	6.28	1.73	0.41	1.49				
sp6	*Sp6 transcription factor*	ENSDARG00000099880	5.89	1.95	0.22	0.67				
si:ch211	Protein coding	ENSDARG00000060927	5.61	2.53	0.34	0.75				
lcp1	*lymphocyte cytosolic protein 1 (L-plastin)*	ENSDARG00000023188	4.46	3.25	0.48	0.66				
f5	coagulation factor V	ENSDARG00000055705	4.40	2.30	0.41	0.79				
apoeb	apolipoprotein Eb	ENSDARG00000040295	4.15	1.79	0.56	1.26				
c1qtnf5	C1q and tumor necrosis factor related protein 5	ENSDARG00000056134	3.88	2.42	0.40	0.64				
snorc	secondary ossification center associated	ENSDARG00000092383	3.71	1.17	0.19	0.62				
txn	thioredoxin	ENSDARG00000044125	3.36	4.22	1.81	1.44				
manf	mesencephalic astrocyte-derived neurotrophic	ENSDARG00000063177	3.22	1.87	0.59	1.02				
loxl5b	*lysyl oxidase-like 5b*	ENSDARG00000076904	3.16	1.33	0.26	0.63				
tram1	translocation associated membrane protein 1	ENSDARG00000019137	2.99	1.36	0.49	1.08				
efemp2a	EGF containing fibulin-like extracellular matrix	ENSDARG00000094324	2.92	1.98	0.61	0.90				
cmpk	cytidylate kinase	ENSDARG00000019924	2.90	2.11	0.52	0.72				
si:dkey	protein coding gene	ENSDARG00000090552	2.83	1.82	0.67	1.05				
hpcal4	hippocalcin like 4	ENSDARG00000070491	2.75	0.85	0.33	1.09				
krt97	keratin 97	ENSDARG00000000212	2.66	1.08	0.30	0.74				
slc39a7	solute carrier family 39 (zinc transporter),	ENSDARG00000104451	2.46	1.53	0.59	0.95				
mcm10	minichromosome maintenance 10 replication	ENSDARG00000045815	2.38	1.71	0.39	0.54				
s100a10b	S100 calcium binding protein A10b	ENSDARG00000025254	2.08	2.58	1.59	1.28				
prps1b	phosphoribosyl pyrophosphate synthetase 1B	ENSDARG00000037506	1.97	1.35	0.56	0.82				
rpa3	replication protein A3	ENSDARG00000002613	1.86	1.47	0.60	0.76				
ywhaqb	tyrosine 3-monooxygenase/tryptophan 5-	ENSDARG00000023323	1.85	2.27	1.54	1.26				
BX511082.1	protein coding gene	ENSDARG00000113678	1.82	0.99	0.52	0.97				
arhgdig	Rho GDP dissociation inhibitor (GDI) gamma	ENSDARG00000004034	1.75	1.17	0.72	1.07				
ap1m1		ENSDARG00000096249	1.67	1.21	0.63	0.86				
cpm	carboxypeptidase M	ENSDARG00000011769	1.66	1.01	0.56	0.91				
ccn1l2		ENSDARG00000099985	1.65	1.15	0.60	0.86				
clta	clathrin, light chain A	ENSDARG00000045618	1.64	1.27	0.56	0.73				
arpp19b	cAMP-regulated phosphoprotein 19b /// cAMP-	ENSDARG00000039880	1.59	1.16	0.58	0.80				
pqbp1	polyglutamine binding protein 1	ENSDARG00000029724	1.58	1.20	0.54	0.72				
nars1	asparaginyl-tRNA synthetase	ENSDARG00000061100	1.57	1.06	0.57	0.84				
sec23a	Sec23 homolog A, COPII coat complex	ENSDARG00000104230	1.56	1.09	0.65	0.94				
dnajc9	DnaJ (Hsp40) homolog, subfamily C, member	ENSDARG00000031293	1.56	1.33	0.62	0.73				
cd81a	CD81 molecule a	ENSDARG00000036080	1.40	1.17	0.71	0.85				
abcb5	ATP-binding cassette, sub-family B	ENSDARG00000021787	0.61	0.84	1.84	1.33				
pmp22a	peripheral myelin protein 22a	ENSDARG00000105223	0.58	0.88	1.57	1.03				
sdhaf4	succinate dehydrogenase complex assembly	ENSDARG00000039390	0.58	0.94	1.94	1.19				
PPM1K		ENSDARG00000076011	0.58	0.66	1.52	1.33				
matn3b		ENSDARG00000069265	0.57	0.39	0.66	0.97				
tmem41ab	transmembrane protein 41ab	ENSDARG00000026771	0.53	0.67	1.70	1.35				
evpla	envoplakin a	ENSDARG00000019808	0.53	1.04	1.91	0.97				
fh	fumarate hydratase	ENSDARG00000075132	0.52	0.84	1.63	1.01				
ucp1	uncoupling protein 1	ENSDARG00000023151	0.49	0.77	1.85	1.17				
si:dkey	protein coding gene	ENSDARG00000101128	0.48	0.66	2.33	1.70				
nmrk2	nicotinamide riboside kinase 2	ENSDARG00000067848	0.45	0.61	1.96	1.43				
lipg	lipase, endothelial	ENSDARG00000031044	0.42	0.74	1.89	1.07				
ankrd9	ankyrin repeat domain 9	ENSDARG00000028804	0.36	0.53	4.21	2.86				
col7a1	collagen, type VII, alpha 1	ENSDARG00000021720	0.21	0.60	2.36	0.84				

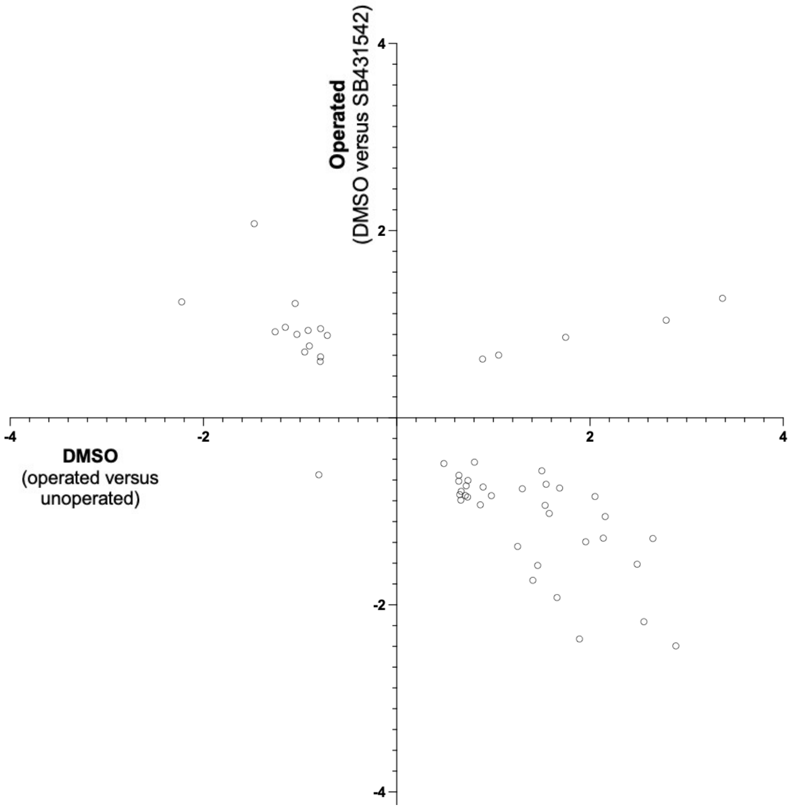

Supplemental Figure 2. Potential TGF-beta dependent DEGs. These are DEGs that have a P_{adj} value < 0.05 for both DMSO operated versus DMSO unoperated and SB431542 operated versus DMSO operated. The columns show the ensemble identifiers, the fold level of change and the adjusted counts for each sample. The graph is the same as in Fig. 3d but only showings DEGs that are significant for both comparisons. Note that the graph is in Log_2 scale and the list shows fold change.

Fold change
>5.0 = red
>3.0 = light red
>1.2 = pale red
<0.85 = pale blue
<0.65 = light blue
<0.5 = blue

Gene counts
>1
>10
>100
>1000

p<0.05 p<0.1

Gene Symbol	Gene Title	ensemble	operated vs unoperated DMSO	operated vs unoperated SB43	SB43 vs DMSO operated	SB43 vs DMSO unoperated
fn1b	fibronectin 1b	ENSDARG00000006526	32.47	21.34	0.67	1.02
cart3		ENSDARG00000035852	18.77	12.90	0.71	1.03
timp2b	TIMP metallopeptidase inhibitor 2b	ENSDARG00000075261	16.15	13.34	0.74	0.90
grn1	granulin 1	ENSDARG00000089362	16.13	10.23	0.80	1.26
si:ch211		ENSDARG00000109648	9.76	5.54	0.75	1.32
ms4a17a.4	membrane-spanning 4-domains	ENSDARG00000014024	9.71	6.57	0.70	1.04
slc7a7	solute carrier family 7 (amino acid transporter	ENSDARG00000055226	7.70	5.15	0.67	1.00
tgm2l	transglutaminase 2, like	ENSDARG00000093381	7.27	2.70	0.78	2.09
adam8a		ENSDARG00000001452	6.52	4.10	0.83	1.32
clu	clusterin	ENSDARG00000010434	6.43	5.29	0.75	0.91
ms4a17a.5	membrane-spanning 4-domains	ENSDARG00000092204	6.24	3.21	0.67	1.30
cbx7a		ENSDARG00000038025	6.16	3.66	0.69	1.16
c7a		ENSDARG00000042172	5.95	4.73	0.60	0.75
ms4a17a.7	membrane-spanning 4-domains	ENSDARG00000043796	5.36	2.64	0.81	1.65
krt18a.1	keratin 18	ENSDARG00000018404	5.05	3.20	0.75	1.18
FO704661.1		ENSDARG00000102758	4.98	3.40	0.81	1.18
ctsk	cathepsin K	ENSDARG00000040251	4.58	2.58	0.82	1.46
lcp1	*lymphocyte cytosolic protein 1 (L-plastin)*	ENSDARG00000023188	4.46	3.25	0.48	0.66
mpeg1.1		ENSDARG00000055290	4.46	2.80	0.70	1.11
kif23	kinesin family member 23	ENSDARG00000014943	4.37	2.50	0.71	1.24
snap23.2		ENSDARG00000055252	4.35	3.77	0.70	0.81
apoeb	apolipoprotein Eb	ENSDARG00000040295	4.15	1.79	0.56	1.26
si:rp71	dispanin subfamily A member 2b-like	ENSDARG00000097746	4.09	2.90	0.75	1.06
ccdc88b		ENSDARG00000076189	3.98	3.15	0.86	1.08
tagln	transgelin	ENSDARG00000045408	3.96	2.87	0.66	0.92
tnnt2c	troponin T2c, cardiac	ENSDARG00000032242	3.90	2.82	0.73	1.01
c1qtnf5	C1q and tumor necrosis factor related protein 5	ENSDARG00000056134	3.88	2.42	0.40	0.64
fcer1gl		ENSDARG00000104077	3.68	2.33	0.68	1.07
spi1b	Spi-1 proto-oncogene b	ENSDARG00000000767	3.65	2.49	0.72	1.06
manf	mesencephalic astrocyte-derived neurotrophic	ENSDARG00000063177	3.22	1.87	0.59	1.02
si:ch211	si:ch211-1a19.3	ENSDARG00000100968	3.10	2.39	0.76	0.99
cdca8	cell division cycle associated 8	ENSDARG00000043137	3.00	2.18	0.73	1.01
efemp2a	EGF containing fibulin-like extracellular matrix	ENSDARG00000094324	2.92	1.98	0.61	0.90
cmpk	cytidylate kinase	ENSDARG00000019924	2.90	2.11	0.52	0.72
si:dkey	protein coding gene	ENSDARG00000090552	2.83	1.82	0.67	1.05
hyou1	hypoxia up-regulated 1	ENSDARG00000013670	2.78	1.84	0.83	1.26
ms4a17a.12	membrane-spanning 4-domains	ENSDARG00000053563	2.74	2.27	0.86	1.04
clic2		ENSDARG00000010625	2.62	1.92	0.82	1.12
pitpnaa	phosphatidylinositol transfer protein, alpha a	ENSDARG00000039490	2.61	2.26	0.76	0.88
il6st	interleukin 6 signal transducer ///	ENSDARG00000104693	2.47	2.04	0.77	0.93
capgb	capping protein (actin filament), gelsolin-like b	ENSDARG00000099672	2.41	1.80	0.83	1.12
ptges3b	prostaglandin E synthase 3b (cytosolic)	ENSDARG00000089626	2.21	1.78	0.85	1.06
rrbp1a	ribosome binding protein 1 homolog a (dog)	ENSDARG00000013763	2.19	1.72	0.73	0.94
dad1	defender against cell death 1	ENSDARG00000102105	2.01	1.54	0.69	0.90
thop1	zgc:92139	ENSDARG00000013776	1.96	1.66	0.79	0.93
tmsb4x	thymosin, beta 4 x	ENSDARG00000077777	1.72	1.47	0.83	0.96
atp2a1l	ATPase, Ca++ transporting, cardiac muscle,	ENSDARG00000035458	0.52	0.69	1.36	1.03
cs	citrate synthase	ENSDARG00000103364	0.50	0.62	1.15	0.93
krt4	keratin 4	ENSDARG00000017624	0.49	0.66	1.33	0.99
casq1a		ENSDARG00000038716	0.48	0.57	1.48	1.24
dnajc18	DnaJ (Hsp40) homolog, subfamily C, member	ENSDARG00000056005	0.47	0.58	1.19	0.97
si:ch211		ENSDARG00000096616	0.46	0.62	1.33	0.98
tuba8l2	tubulin, alpha 8 like 2	ENSDARG00000031164	0.45	0.56	1.17	0.94
fndc1		ENSDARG00000002847	0.40	0.50	1.28	1.03
nrap		ENSDARG00000009341	0.35	0.41	1.27	1.08
col2a1a	collagen, type II, alpha 1a	ENSDARG00000069093	0.32	0.37	1.77	1.51
col11a2	collagen, type XI, alpha 2	ENSDARG00000012422	0.30	0.38	1.16	0.92
epyc		ENSDARG00000056950	0.27	0.42	1.21	0.77

DMSO unoperated COUNT, DMSO operated COUNT, SB43 unoperated COUNT, SB43 operated COUNT

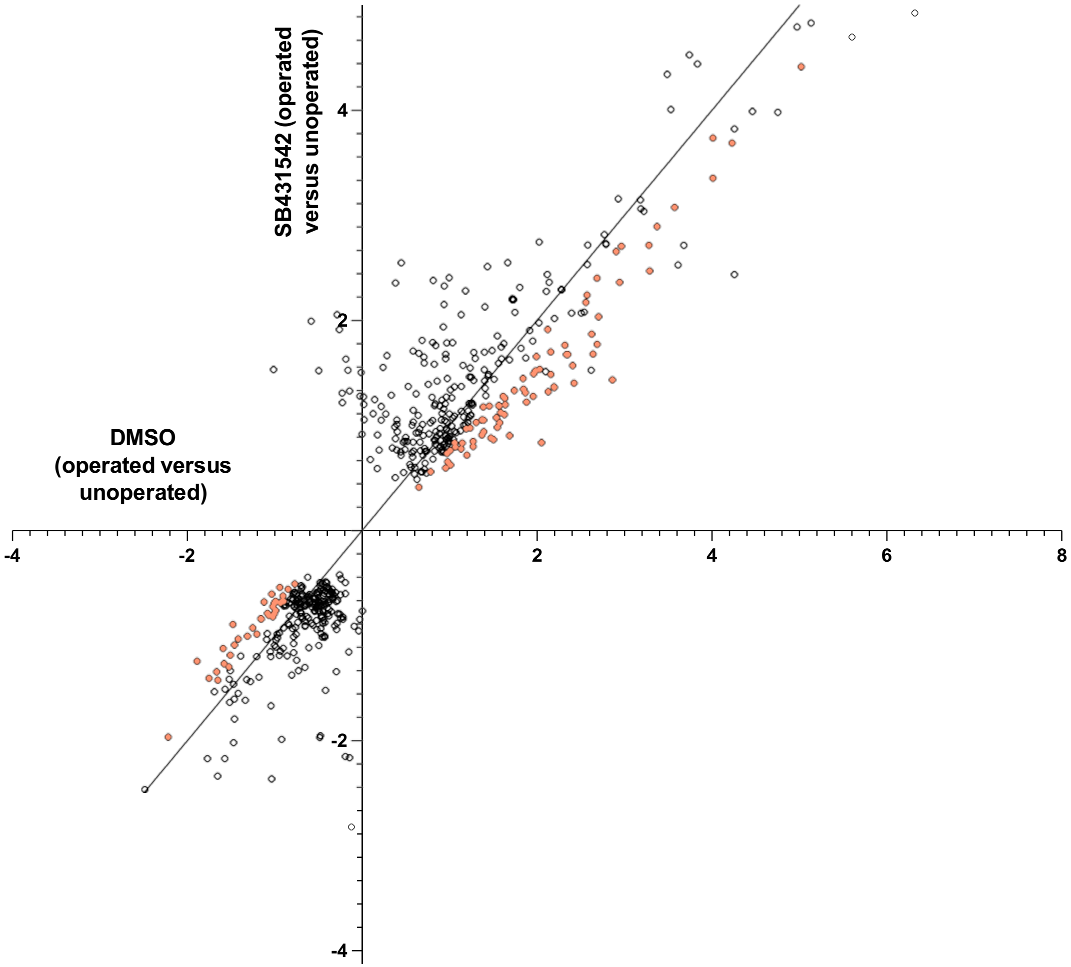

Supplemental Figure 3. Potential regeneration DEGs. To generate this table DEGs must meet all of the following criteria (1) a gene count of >10 for all conditions (2) a P_{adj} value <0.05 for DMSO operated versus unoperated and for SB431542 operated versus unoperated. (3) Either a Log_2 score above 0 for SB431542 operated versus unoperated and a ratio below 0.87 for SB431542 operated versus unoperated/ DMSO operated versus unoperated (coloured DEGs in the top right quadrant of graph) OR a Log_2 score below 0 for SB431542 operated versus unoperated and a ratio above 1.15 for SB431542 operated versus unoperated/ DMSO operated versus unoperated (coloured DEGs in the lower left quadrant of graph). The columns show the ensemble identifiers, the fold level of change and the adjusted counts for each sample. Note that the graph is the same data as in Fig 3e with the line indicating a slope of one. The graph is in Log_2 scale and the list shows fold change.

Fold change
>5.0 = red
>3.0 = light red
>1.2 = pale red
<0.85 = pale blue
<0.65 = light blue
<0.5 = blue

Gene counts
>1
>10
>100
>1000

p<0.05 p<0.1

Gene Symbol	Gene Title	ensemble	operated vs unoperated DMSO	operated vs unoperated SB43	SB43 vs DMSO operated	SB43 vs DMSO unoperated	DMSO unoperated COUNT	DMSO operated COUNT	SB43 unoperated COUNT	SB43 operated COUNT
si:ch211	protein coding gene	ENSDARG00000074322	9.32	8.22	1.14	1.29				
rrad	Ras-related associated with diabetes	ENSDARG00000052011	5.98	6.58	1.05	0.95				
zgc:172053	protein coding gene	ENSDARG00000038321	4.60	4.06	1.04	1.18				
lmna	lamin A	ENSDARG00000013415	3.52	3.33	1.11	1.18				
irg1l	immunoresponsive gene 1, like	ENSDARG00000062788	3.21	3.09	1.12	1.17				
fanci	Fanconi anemia, complementation group I	ENSDARG00000026224	3.07	3.43	0.93	0.83				
parp4		ENSDARG00000069934	3.02	3.36	1.05	0.94				
ms4a17a.11	membrane-spanning 4-domains	ENSDARG00000094809	2.71	2.77	0.88	0.86				
lrpap1	low density lipoprotein receptor-related protein	ENSDARG00000033604	2.42	2.69	0.90	0.81				
lect2l		ENSDARG00000033227	2.39	2.32	0.97	1.00				
ube2c	ubiquitin-conjugating enzyme E2C	ENSDARG00000114670	2.33	2.11	0.88	0.97				
slc38a8b	putative sodium-coupled neutral amino acid	ENSDARG00000054196	2.31	2.04	1.11	1.26				
ctsc	cathepsin C	ENSDARG00000101334	2.27	2.01	0.90	1.01				
tcirg1b	T-cell, immune regulator 1, ATPase, H+	ENSDARG00000105142	2.24	2.01	1.07	1.19				
hsp90b1	heat shock protein 90, beta (grp94), member 1	ENSDARG00000003570	2.00	1.86	0.95	1.02				
adamtsl5		ENSDARG00000052118	1.99	2.16	1.03	0.95				
thbs1a	thrombospondin-1-like	ENSDARG00000103775	1.99	1.81	1.07	1.18				
actn4	actinin, alpha 4	ENSDARG00000099786	1.95	2.05	1.02	0.97				
tgfb1a	transforming growth factor, beta 1a	ENSDARG00000041502	1.95	1.83	0.91	0.97				
si:ch1073		ENSDARG00000102599	1.88	1.70	0.96	1.06				
rnaseka	ribonuclease, RNase K a	ENSDARG00000069461	1.87	1.68	0.94	1.06				
ssrp1a	structure specific recognition protein 1a	ENSDARG00000037397	1.86	1.96	1.03	0.97				
prkcsh	protein kinase C substrate 80K-H	ENSDARG00000004470	1.85	1.90	1.12	1.09				
mbd2		ENSDARG00000075952	1.81	1.86	0.96	0.93				
anxa2a	annexin A2a	ENSDARG00000003216	1.77	1.82	1.06	1.04				
sat1a.2		ENSDARG00000095908	1.70	1.89	0.95	0.85				
ctsd	cathepsin D	ENSDARG00000057698	1.67	1.46	1.09	1.24				
cirbpa	cold inducible RNA binding protein a	ENSDARG00000103672	1.65	1.69	0.90	0.89				
arpc3	actin related protein 2/3 complex, subunit 3	ENSDARG00000057882	1.64	1.50	0.95	1.04				
eif5a2	eukaryotic translation initiation factor 5A2	ENSDARG00000056186	1.62	1.64	0.90	0.89				
col6a3		ENSDARG00000077139	1.58	1.79	1.02	0.90				
mkrn1	makorin, ring finger protein, 1	ENSDARG00000041665	1.52	1.52	0.91	0.92				
flna		ENSDARG00000074201	1.47	1.52	0.93	0.90				
rab1ab	RAB1A, member RAS oncogene family b	ENSDARG00000029663	1.46	1.55	1.12	1.05				
hhatlb	hedgehog acyltransferase-like, b	ENSDARG00000005139	0.73	0.68	0.91	0.99				
atp5mea	ATP synthase, H+ transporting, mitochondrial	ENSDARG00000078113	0.73	0.71	1.02	1.06				
col1a1a	collagen, type I, alpha 1a	ENSDARG00000012405	0.72	0.68	1.01	1.07				
stxbp1a		ENSDARG00000001994	0.72	0.71	0.94	0.94				
bckdha	branched chain keto acid dehydrogenase E1,	ENSDARG00000040555	0.71	0.63	0.92	1.05				
si:ch211		ENSDARG00000092035	0.70	0.65	1.11	1.19				
mt-cyb		ENSDARG00000063924	0.70	0.66	1.04	1.09				
adss1	adenylosuccinate synthase like 1	ENSDARG00000099517	0.69	0.64	0.94	1.00				
pbx3b	pre-B-cell leukemia homeobox 3b	ENSDARG00000013615	0.68	0.64	0.91	0.98				
mt-nd4		ENSDARG00000063917	0.67	0.62	0.95	1.04				
aldoab	aldolase a, fructose-bisphosphate, b	ENSDARG00000034470	0.66	0.63	0.97	1.02				
gys1		ENSDARG00000016875	0.66	0.64	0.93	0.96				
pvalb4	parvalbumin 4	ENSDARG00000024433	0.66	0.66	0.99	0.99				
synpo2la		ENSDARG00000077293	0.66	0.62	0.98	1.04				
mt-co2		ENSDARG00000063908	0.65	0.65	1.01	1.01				
tnni4b.2	troponin I4b, tandem duplicate 2	ENSDARG00000036671	0.65	0.74	1.14	1.01				
slc38a4	solute carrier family 38, member 4	ENSDARG00000018149	0.65	0.64	0.94	0.94				
ldb3a	LIM domain binding 3a	ENSDARG00000056322	0.64	0.62	0.91	0.95				
pdha1a	pyruvate dehydrogenase (lipoamide) alpha 1a	ENSDARG00000012387	0.63	0.61	0.93	0.97				
mybpc3	myosin binding protein C, cardiac	ENSDARG00000011615	0.63	0.61	0.98	1.02				
cish	cytokine inducible SH2-containing protein	ENSDARG00000060316	0.62	0.56	0.88	0.98				
enah	enabled homolog (Drosophila)	ENSDARG00000032049	0.62	0.65	0.94	0.91				
tnmd		ENSDARG00000052615	0.62	0.69	1.14	1.03				
pabpc4	poly(A) binding protein, cytoplasmic 4	ENSDARG00000059259	0.62	0.66	0.98	0.93				
camk2b1	calcium/calmodulin-dependent protein kinase II	ENSDARG00000011065	0.62	0.58	1.01	1.08				
mbpa	myelin basic protein a	ENSDARG00000036186	0.62	0.65	0.98	0.94				
uqcrb	ubiquinol-cytochrome c reductase binding	ENSDARG00000011146	0.62	0.70	1.04	0.92				
tnni1d	troponin I, skeletal, slow d	ENSDARG00000073766	0.62	0.57	0.89	0.97				
actn3a	actinin alpha 3a	ENSDARG00000013755	0.61	0.58	1.03	1.09				
vimr2	vimentin-related gene	ENSDARG00000105188	0.61	0.60	0.94	0.95				
mylz3	myosin, light polypeptide 3, skeletal muscle	ENSDARG00000017441	0.61	0.64	0.99	0.94				
hsc70	heat shock cognate 70	ENSDARG00000012381	0.61	0.62	1.03	1.00				
cfl2		ENSDARG00000014106	0.60	0.61	1.00	0.97				
rbfox1l	RNA binding protein, fox-1 homolog (C.	ENSDARG00000021184	0.59	0.58	1.03	1.06				
chrnd		ENSDARG00000019342	0.59	0.60	0.89	0.88				
atp5f1b	ATP synthase, H+ transporting, mitochondrial	ENSDARG00000070083	0.59	0.67	0.96	0.84				
myom2a	si:dkey-78p20.4	ENSDARG00000075433	0.58	0.60	0.90	0.87				
zgc:110843	zgc:110843	ENSDARG00000073845	0.58	0.61	0.97	0.93				
atp1b3b	ATPase, Na+/K+ transporting, beta 3b	ENSDARG00000042837	0.58	0.66	0.98	0.86				
proza	protein Z, vitamin K-dependent plasma	ENSDARG00000037783	0.58	0.63	1.09	0.99				
myhz2	myosin, heavy polypeptide 2, fast muscle	ENSDARG00000012944	0.58	0.64	0.91	0.82				
ndst1a		ENSDARG00000062397	0.56	0.57	0.94	0.93				
tnnt3b	troponin T type 3b (skeletal, fast)	ENSDARG00000068457	0.55	0.61	1.02	0.92				
mt-nd2		ENSDARG00000063899	0.55	0.62	1.00	0.87				
slc25a4	solute carrier family 25 (mitochondrial carrier;	ENSDARG00000027355	0.54	0.61	0.97	0.85				
pvalb3	parvalbumin 3	ENSDARG00000022817	0.54	0.61	1.10	0.97				
krtt1c19e	keratin type 1 c19e	ENSDARG00000090268	0.47	0.51	0.96	0.90				
sncga		ENSDARG00000034423	0.37	0.34	1.07	1.17				
sparcl1		ENSDARG00000074989	0.36	0.33	1.03	1.14				
chad		ENSDARG00000045071	0.36	0.36	0.98	0.97				
col9a1b	collagen, type IX, alpha 1b	ENSDARG00000031483	0.35	0.40	1.13	1.00				
palmda	palmdelphin a	ENSDARG00000001913	0.35	0.32	1.01	1.10				
si:dkey	protein coding gene	ENSDARG00000057504	0.18	0.18	1.03	1.01				

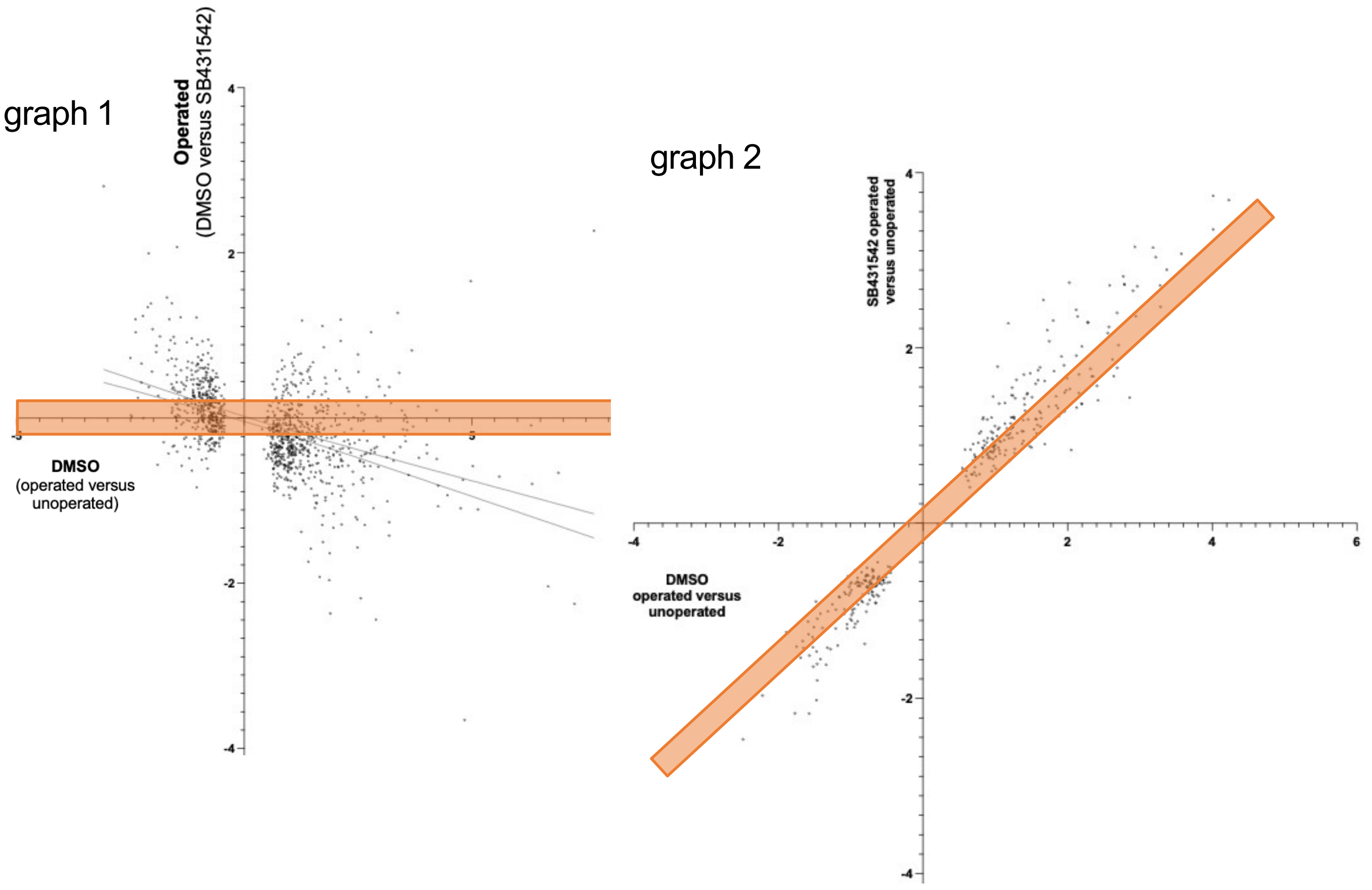

Supplemental Figure 4. Potential regeneration DEGs that are not strongly regulated by TGF-beta. To generate this table DEGs must meet all of the following criteria (1) a gene count of >10 for all conditions (2) a P_{adj} value < 0.05 for DMSO operated versus DMSO unoperated and a Log_2 score of between 0.2 and -0.2 in the SB431542 operated versus DMSO operated comparison (box in graph 1) (3) a P_{adj} value < 0.05 for SB431542 operated versus unoperated (4) a similar expression change between DMSO operated versus DMSO unoperated and SB431542 operated versus SB431542 unoperated (ratio between 0.87 and 1.15) (box in graph 2). The columns show the ensemble identifiers, the fold level of change and the adjusted counts for each sample. Note that the graph is in Log_2 scale and the list shows fold change. Graph 1 is data from Figure 3d and graph 2 is Figure 3e.

Supplemental Figure 5. Comparison of families of genes within RNA-seq data. This table shows families of genes with five or more members that were detected in our RNA-seq experiments. The columns show the ensemble identifiers, the fold level of change and the adjusted counts for each sample.

Fold change
- >5.0 = red
- >3.0 = light red
- >1.2 = pale red
- <0.85 = pale blue
- <0.65 = light blue
- <0.5 = blue

Gene counts
- >1
- >10
- >100
- >1000

p<0.05 p<0.1

Gene Symbol	Gene Title	ensemble	(1) DMSO (operated vs unoperated)	(2) Operated (SB431542 vs DMSO)	(3) Unoperated (SB431542 vs DMSO)	(4) SB431542 (operated vs unoperated)	DMSO unoperated COUNT	DMSO operated COUNT	SB43 unoperated COUNT	SB43 operated COUNT
abcb4	ATP-binding cassette, sub-family B	ENSDARG00000010936	7.14	0.47	1.15	2.92				
abcc10	ATP-binding cassette, sub-family C	ENSDARG00000077988	2.16	1.90	2.18	1.88				
abcf2a	ATP-binding cassette, sub-family F (GCN20),	ENSDARG00000038785	1.73	0.93	1.10	1.47				
abca1a	ATP-binding cassette, sub-family A (ABC1),	ENSDARG00000074635	1.52	0.85	1.15	1.12				
abce1	ATP-binding cassette, sub-family E (OABP),	ENSDARG00000007216	1.48	0.69	0.85	1.20				
abca1b	ATP-binding cassette, sub-family A (ABC1),	ENSDARG00000079009	1.27	0.90	0.66	1.73				
abcf1	ATP-binding cassette, sub-family F (GCN20),	ENSDARG00000031795	1.20	0.82	0.73	1.34				
abca5	ATP-binding cassette, sub-family A (ABC1),	ENSDARG00000074041	1.03	1.03	0.78	1.35				
abca3b	ATP-binding cassette, sub-family A (ABC1),	ENSDARG00000100524	1.02	1.13	0.94	1.23				
abcb7	ATP-binding cassette, sub-family B	ENSDARG00000062795	0.96	0.87	1.07	0.78				
abcd3a	ATP-binding cassette, sub-family D (ALD),	ENSDARG00000104085	0.93	0.74	0.83	0.83				
abcc5	ATP-binding cassette, sub-family C	ENSDARG00000061233	0.88	2.13	0.60	3.10				
abcb8	ATP-binding cassette, sub-family B	ENSDARG00000056672	0.64	2.31	1.88	0.78				
abcb5	ATP-binding cassette, sub-family B	ENSDARG00000021787	0.61	1.84	1.33	0.84				
abcd4	ATP-binding cassette, sub-family D (ALD),	ENSDARG00000061770	0.57	4.75	2.48	1.10				
actr2a	ARP2 actin-related protein 2a homolog (yeast)	ENSDARG00000052438	2.98	1.08	1.58	2.04				
actb1	actin, beta 1	ENSDARG00000037746	1.72	0.99	0.83	2.04				
actb2	actin, beta 2	ENSDARG00000037870	1.24	1.02	1.10	1.15				
actl6a	actin-like 6A	ENSDARG00000070828	1.06	1.21	0.72	1.77				
actr6	ARP6 actin-related protein 6 homolog (yeast)	ENSDARG00000021370	1.05	2.37	1.84	1.36				
actr1	ARP1 actin-related protein 1, centractin (yeast)	ENSDARG00000011611	1.05	1.21	1.10	1.16				
actr8	ARP8 actin-related protein 8 homolog (yeast)	ENSDARG00000103610	1.05	0.64	1.01	0.66				
actr10	actin-related protein 10 homolog (S. cerevisiae)	ENSDARG00000038432	0.94	0.84	0.72	1.10				
actc1b	actin, alpha, cardiac muscle 1b	ENSDARG00000099197	0.69	0.83	0.97	0.59				
acta2	actin, alpha 2, smooth muscle, aorta	ENSDARG00000045180	0.64	0.84	0.69	0.78				
actc1	actin, alpha, cardiac muscle 2	ENSDARG00000057911	0.64	0.79	1.23	0.41				
actc1a	actin alpha cardiac muscle 1a	ENSDARG00000042535	0.59	0.83	0.93	0.53				
acta1b	actin alpha 1, skeletal muscle b	ENSDARG00000055618	0.50	0.98	0.84	0.58				
acta1a	actin, alpha 1a, skeletal muscle	ENSDARG00000036371	0.35	1.01	0.81	0.44				
actc1c	actin alpha cardiac muscle 1c	ENSDARG00000079111	0.21	1.00	0.84	0.26				
actn1	actinin, alpha 1	ENSDARG00000007219	2.58	3.11	4.51	1.78				
actn4	actinin, alpha 4	ENSDARG00000099786	1.95	1.02	0.97	2.05				
actn3b	actinin alpha 3b	ENSDARG00000001431	0.78	1.15	1.35	0.67				
actn2b	actinin, alpha 2b	ENSDARG00000071090	0.73	0.70	0.74	0.69				
actn3a	actinin alpha 3a	ENSDARG00000013755	0.61	1.03	1.09	0.58				
ankrd10a	ankyrin repeat domain 10a	ENSDARG00000037100	1.81	0.79	0.96	1.50				
ankrd49	ankyrin repeat domain 49	ENSDARG00000023508	1.53	0.90	1.33	1.04				
ankmy2a	ankyrin repeat and MYND domain containing	ENSDARG00000005948	1.48	0.50	0.96	0.77				
ankfy1	ankyrin repeat and FYVE domain containing 1	ENSDARG00000061013	1.22	0.78	0.89	1.07				
ankrd28b	ankyrin repeat domain 28b	ENSDARG00000009023	1.12	1.13	1.27	1.00				
ankar	ankyrin and armadillo repeat containing	ENSDARG00000000516	1.00	2.31	1.00	1.70				
ankhd1	ankyrin repeat and KH domain containing 1	ENSDARG00000077860	0.97	1.05	0.86	1.18				
ankra2	ankyrin repeat, family A (RFXANK-like), 2	ENSDARG00000035399	0.95	0.97	1.33	0.69				
ankrd24	ankyrin repeat domain 24	ENSDARG00000062103	0.95	0.40	0.69	0.55				
ankmy1	ankyrin repeat and MYND domain containing 1	ENSDARG00000062702	0.94	2.15	0.78	2.61				
ankrd13c	ankyrin repeat domain 13C	ENSDARG00000103831	0.93	0.89	0.91	0.91				
anks1b	ankyrin repeat and sterile alpha motif domain	ENSDARG00000003512	0.79	0.79	0.90	0.69				
ankrd22	ankyrin repeat domain 22	ENSDARG00000002298	0.76	1.48	1.41	0.79				
ankrd46b	ankyrin repeat domain 46b	ENSDARG00000015780	0.71	1.38	1.56	0.63				

Fold change
>5.0 = red
>3.0 = light red
>1.2 = pale red
<0.85 = pale blue
<0.65 = light blue
<0.5 = blue

Gene counts
>1
>10
>100
>1000

p<0.05 p<0.1

Gene Symbol	Gene Title	ensemble	(1) DMSO (operated vs unoperated)	(2) Operated (SB431542 vs DMSO)	(3) Unoperated (SB431542 vs DMSO)	(4) SB431542 (operated vs unoperated)
ankrd12	ankyrin repeat domain 12	ENSDARG00000052419	0.68	1.00	0.67	1.02
ank2b	ankyrin 2b, neuronal	ENSDARG00000043313	0.59	1.65	0.89	1.10
ankrd1b	ankyrin repeat domain 1b (cardiac muscle)	ENSDARG00000076192	0.53	1.23	1.43	0.46
ankrd11	ankyrin repeat domain 11	ENSDARG00000051886	0.52	1.86	0.83	1.16
ank1a	ankyrin 1, erythrocytic a	ENSDARG00000092143	0.40	1.18	0.75	0.63
ankrd9	ankyrin repeat domain 9	ENSDARG00000028804	0.36	4.21	2.86	0.53
ankrd1a	ankyrin repeat domain 1a (cardiac muscle)	ENSDARG00000075263	0.28	1.99	1.15	0.48

Gene Symbol	Gene Title	ensemble	(1)	(2)	(3)	(4)
apoda.2	apolipoprotein Da, duplicate 2	ENSDARG00000060350	14.99	0.38	7.41	0.77
apoba	apolipoprotein Ba	ENSDARG00000042780	11.88	0.07	3.53	0.24
apobb.1	apolipoprotein Bb, tandem duplicate 1	ENSDARG00000022767	8.78	0.12	3.74	0.28
apoea	apolipoprotein Ea	ENSDARG00000102004	7.70	0.38	4.57	0.63
apoa4b.2	apolipoprotein A-IV b, tandem duplicate 2	ENSDARG00000020866	7.45	0.06	2.89	0.15
apoa1a	apolipoprotein A-Ia	ENSDARG00000012076	7.29	0.75	3.44	1.59
apoc1	apolipoprotein C-I like	ENSDARG00000092170	5.77	0.83	2.29	2.10
apobb.2	apolipoprotein B-100	ENSDARG00000075016	5.56	0.15	1.00	1.00
apoa2	apolipoprotein A-II	ENSDARG00000015866	4.40	0.43	1.55	1.23
apoeb	apolipoprotein Eb	ENSDARG00000040295	4.15	0.56	1.26	1.79
apoa4b.1	apolipoprotein A-IV b, tandem duplicate 1	ENSDARG00000040298	3.26	0.46	0.93	1.62
apoa1b	apolipoprotein A-Ib	ENSDARG00000101324	1.61	1.06	1.54	1.11
apodb	apolipoprotein Db	ENSDARG00000057437	1.34	1.74	2.01	1.16
apoa4a	apolipoprotein A-IV a	ENSDARG00000101160	1.00	6.86	3.04	1.63
apoob	apolipoprotein O, b	ENSDARG00000026444	0.99	0.76	0.90	0.84
apol1	apolipoprotein L, 1	ENSDARG00000007425	0.96	1.24	1.68	0.71
apool	apolipoprotein O-like	ENSDARG00000039374	0.94	1.05	1.42	0.69
apoc2	apolipoprotein C-II	ENSDARG00000092155	0.84	0.83	0.33	2.07
apooa	apolipoprotein O, a	ENSDARG00000104696	0.74	1.15	1.11	0.76
apof	apolipoprotein F	ENSDARG00000090980	0.68	2.83	3.01	0.64

Gene Symbol	Gene Title	ensemble	(1)	(2)	(3)	(4)
arpc5a	actin related protein 2/3 complex, subunit 5A	ENSDARG00000039142	2.95	0.78	0.76	3.01
arpc5b	actin related protein 2/3 complex, subunit 5B	ENSDARG00000019062	2.61	0.95	2.39	1.04
arpc1b	actin related protein 2/3 complex, subunit 1B	ENSDARG00000027063	2.24	1.01	1.31	1.73
arpc3	actin related protein 2/3 complex, subunit 3	ENSDARG00000057882	1.64	0.95	1.04	1.50
arpc4	actin related protein 2/3 complex, subunit 4	ENSDARG00000054063	1.61	0.94	1.17	1.29
arpc2	actin related protein 2/3 complex, subunit 2	ENSDARG00000075989	1.61	0.78	0.93	1.35
arpc4l	actin related protein 2/3 complex, subunit 4,	ENSDARG00000058225	1.32	1.41	1.34	1.39
arpc1a	actin related protein 2/3 complex, subunit 1A	ENSDARG00000008383	1.18	0.83	0.76	1.29

Gene Symbol	Gene Title	ensemble	(1)	(2)	(3)	(4)
atp9b	ATPase, class II, type 9B	ENSDARG00000062521	20.41	0.47	16.43	0.58
atp2a2b	ATPase, Ca++ transporting, cardiac muscle,	ENSDARG00000005122	2.82	0.89	1.61	1.57
atp6v0b	ATPase H+ transporting V0 subunit b	ENSDARG00000031681	2.75	1.38	2.14	1.77
atp6v1ba	ATPase, H+ transporting, lysosomal, V1	ENSDARG00000013443	2.07	0.75	1.10	1.41
atp6ap1b	ATPase, H+ transporting, lysosomal accessory	ENSDARG00000037153	2.07	0.73	1.17	1.30
atp6v0ca	ATPase, H+ transporting, lysosomal, V0	ENSDARG00000057853	1.96	0.65	0.80	1.58
atp6v1ab	ATPase, H+ transporting, lysosomal, V1	ENSDARG00000076318	1.75	0.86	1.33	1.13
atp6v1f	ATPase, H+ transporting, lysosomal, V1	ENSDARG00000045543	1.61	0.63	0.97	1.05
atp6v1h	ATPase, H+ transporting, lysosomal V1 subunit	ENSDARG00000006370	1.59	0.75	1.04	1.15
atp6v1aa	ATPase H+ transporting V1 subunit Aa	ENSDARG00000034534	1.43	0.80	0.64	1.80
atp6v1d	ATPase, H+ transporting, lysosomal V1 subunit	ENSDARG00000011175	1.36	0.69	0.84	1.11
atp6v1e1b	ATPase, H+ transporting, lysosomal, V1	ENSDARG00000030694	1.33	0.85	0.94	1.20
atp1a1a.1	ATPase, Na+/K+ transporting, alpha 1a	ENSDARG00000002791	1.30	0.96	0.88	1.42
atp6v1g1	ATPase, H+ transporting, lysosomal, V1	ENSDARG00000022315	1.29	0.95	1.13	1.08
atp6ap2	ATPase, H+ transporting, lysosomal accessory	ENSDARG00000008735	1.29	0.80	1.04	0.99

Fold change
>5.0 = red
>3.0 = light red
>1.2 = pale red
<0.85 = pale blue
<0.65 = light blue
<0.5 = blue

p<0.05 p<0.1

Gene counts
>1
>10
>100
>1000

Gene Symbol	Gene Title	ensemble	(1) DMSO (operated vs unoperated)	(2) Operated (SB431542 vs DMSO)	(3) Unoperated (SB431542 vs DMSO)	(4) SB431542 (operated vs unoperated)	DMSO unoperated COUNT	DMSO operated COUNT	SB43 unoperated COUNT	SB43 operated COUNT
atp6v1c1b	ATPase, H+ transporting, lysosomal, V1	ENSDARG00000035880	1.28	0.79	0.74	1.36				
atp1b1b	ATPase, Na+/K+ transporting, beta 1b	ENSDARG00000076833	1.24	1.06	1.49	0.88				
atp6v0d1	ATPase, H+ transporting, lysosomal V0 subunit	ENSDARG00000069090	1.23	1.03	1.21	1.05				
atp6v1c1a	ATPase, H+ transporting, lysosomal, V1	ENSDARG00000023967	1.13	1.40	1.46	1.09				
atp13a1	ATPase type 13A1	ENSDARG00000029931	1.13	1.00	0.60	1.88				
atp1b2b	ATPase, Na+/K+ transporting, beta 2b	ENSDARG00000034424	1.12	0.59	0.52	1.26				
atp1b1a	ATPase, Na+/K+ transporting, beta 1a	ENSDARG00000013144	1.12	0.79	0.81	1.09				
atp1b2a	ATPase, Na+/K+ transporting, beta 2a	ENSDARG00000099203	1.08	0.96	1.36	0.77				
atp1a1a.2	ATPase, Na+/K+ transporting, alpha 1a	ENSDARG00000007739	1.03	0.88	1.15	0.79				
atp5if1b	ATPase inhibitory factor 1b	ENSDARG00000044092	0.97	1.15	1.50	0.75				
atp1a1b	ATPase, Na+/K+ transporting, alpha 1b	ENSDARG00000019856	0.96	0.83	0.79	1.01				
atpv0e2	ATPase, H+ transporting V0 subunit e2	ENSDARG00000059057	0.91	1.00	1.64	0.56				
atp10b	ATPase, class V, type 10B	ENSDARG00000076230	0.86	1.12	1.10	0.88				
atp1b3a	ATPase, Na+/K+ transporting, beta 3a	ENSDARG00000015790	0.84	1.08	1.07	0.84				
atp6v1b2	ATPase, H+ transporting, lysosomal, V1	ENSDARG00000043465	0.82	1.17	1.00	0.96				
atp2b4	ATPase, Ca++ transporting, plasma membrane	ENSDARG00000044902	0.80	1.01	0.76	1.07				
atp2a1	ATPase, Ca++ transporting, cardiac muscle,	ENSDARG00000020574	0.80	1.12	1.13	0.79				
atp2b3b	ATPase plasma membrane Ca2+ transporting	ENSDARG00000023445	0.76	0.94	1.16	0.62				
atp2b2	ATPase, Ca++ transporting, plasma membrane	ENSDARG00000063433	0.76	0.96	0.82	0.89				
atp1a3b	ATPase, Na+/K+ transporting, alpha 3b	ENSDARG00000104139	0.76	1.03	0.77	1.01				
atp2b3a	ATPase, Ca++ transporting, plasma membrane	ENSDARG00000043474	0.76	1.06	0.75	1.08				
atp1a2a	ATPase, Na+/K+ transporting, alpha 2a	ENSDARG00000010472	0.74	1.72	1.63	0.79				
atp1a3a	ATPase, Na+/K+ transporting, alpha 3a	ENSDARG00000018259	0.74	1.13	0.93	0.89				
atp2b1a	ATPase, Ca++ transporting, plasma membrane	ENSDARG00000012684	0.71	0.83	0.75	0.79				
atp5if1a	ATPase inhibitory factor 1a	ENSDARG00000067975	0.70	0.76	0.70	0.76				
atp6ap1la	ATPase, H+ transporting, lysosomal accessory	ENSDARG00000091509	0.63	0.76	0.61	0.79				
atp1b3b	ATPase, Na+/K+ transporting, beta 3b	ENSDARG00000042837	0.58	0.98	0.86	0.66				
atp2a2a	ATPase, Ca++ transporting, cardiac muscle,	ENSDARG00000029439	0.56	0.98	0.73	0.75				
atp6v0cb	ATPase, H+ transporting, lysosomal, V0	ENSDARG00000036577	0.54	1.07	0.80	0.73				
atp2a1l	ATPase, Ca++ transporting, cardiac muscle,	ENSDARG00000035458	0.52	1.36	1.03	0.69				
atp1a1a.4	ATPase, Na+/K+ transporting, alpha 1a	ENSDARG00000001870	0.52	1.70	0.85	1.04				
chd4a	chromodomain helicase DNA binding protein	ENSDARG00000063535	1.32	0.98	0.76	1.70				
chd1	chromodomain helicase DNA binding protein 1	ENSDARG00000103787	1.24	0.93	0.94	1.23				
chd1l	chromodomain helicase DNA binding protein 1-	ENSDARG00000015471	1.18	0.85	1.00	1.01				
chd4b	chromodomain helicase DNA binding protein	ENSDARG00000025789	1.05	1.05	0.94	1.17				
chd7	chromodomain helicase DNA binding protein 7	ENSDARG00000075211	0.88	0.98	0.78	1.11				
chd2	chromodomain helicase DNA binding protein 2	ENSDARG00000060687	0.81	1.25	0.91	1.11				
chd9	chromodomain helicase DNA binding protein 9	ENSDARG00000074498	0.77	1.03	0.86	0.92				
chd6	chromodomain helicase DNA binding protein 6	ENSDARG00000017244	0.75	0.87	0.65	1.00				
chd5	chromodomain helicase DNA binding protein 5	ENSDARG00000105083	0.57	0.74	0.45	0.93				
chd3	chromodomain helicase DNA binding protein 3	ENSDARG00000021405	0.53	0.98	0.72	0.72				
cldn5a	claudin 5a	ENSDARG00000043716	1.52	2.35	0.93	3.85				
cldni	claudin i	ENSDARG00000054616	1.42	1.01	1.04	1.38				
cldne	claudin e	ENSDARG00000043128	1.23	1.37	1.54	1.10				
cldn7b	claudin 7b	ENSDARG00000014047	1.19	1.45	1.52	1.13				
cldn15la	claudin 15-like a	ENSDARG00000016081	1.15	1.63	2.68	0.70				
cldn12	claudin 12	ENSDARG00000003927	1.10	0.77	0.88	0.96				
cldna	claudin a	ENSDARG00000069888	0.91	3.36	1.21	2.51				
cldnb	claudin b	ENSDARG00000009544	0.88	1.84	1.57	1.03				
cldnh	claudin h	ENSDARG00000069503	0.79	1.42	1.11	1.02				
cldn11b	claudin 11b	ENSDARG00000030723	0.78	0.91	0.92	0.76				

Fold change
>5.0 = red
>3.0 = light red
>1.2 = pale red
<0.85 = pale blue
<0.65 = light blue
<0.5 = blue

Gene counts
>1
>10
>100
>1000

p<0.05 p<0.1

Gene Symbol	Gene Title	ensemble	(1) DMSO (operated vs unoperated)	(2) Operated (SB431542 vs DMSO)	(3) Unoperated (SB431542 vs DMSO)	(4) SB431542 (operated vs unoperated)	DMSO unoperated COUNT	DMSO operated COUNT	SB43 unoperated COUNT	SB43 operated COUNT
cldng	claudin g	ENSDARG00000003701	0.74	0.87	0.92	0.71				
cldnk	claudin k	ENSDARG00000042357	0.74	1.05	1.33	0.58				
cldn8.3	claudin 8.3	ENSDARG00000099950	0.72	1.60	4.43	0.26				
cldnd1a	claudin domain containing 1a	ENSDARG00000104439	0.71	0.74	0.45	1.16				
cldnc	claudin c	ENSDARG00000015955	0.70	2.99	1.86	1.13				
cldn19	claudin 19	ENSDARG00000044569	0.61	1.87	0.86	1.34				
cldnd	claudin d	ENSDARG00000006580	0.59	0.85	1.00	0.50				
cldn11a	claudin 11a	ENSDARG00000020031	0.50	0.91	0.86	0.53				
col17a1b	collagen, type XVII, alpha 1b	ENSDARG00000079011	1.46	0.87	1.04	1.23				
col12a1a	collagen, type XII, alpha 1a	ENSDARG00000078322	1.45	0.81	0.94	1.26				
col5a3a	collagen type V alpha-3a	ENSDARG00000098294	1.33	0.58	0.20	3.92				
col5a2a	collagen, type V, alpha 2a	ENSDARG00000031678	1.13	0.96	1.09	1.00				
col8a1a	collagen, type VIII, alpha 1a	ENSDARG00000077403	1.00	19.40	1.00	14.29				
col4a1	collagen, type IV, alpha 1	ENSDARG00000055009	1.00	1.04	0.83	1.25				
col5a1	procollagen, type V, alpha 1	ENSDARG00000012593	0.91	0.84	0.85	0.90				
col28a2a	collagen type XXVIII alpha 1 a	ENSDARG00000076321	0.88	0.77	1.04	0.65				
col15a1b	collagen, type XV, alpha 1b	ENSDARG00000061848	0.84	0.58	1.29	0.38				
col1a2	collagen, type I, alpha 2	ENSDARG00000020007	0.77	0.89	0.90	0.76				
col1a1b	collagen, type I, alpha 1b	ENSDARG00000035809	0.76	1.17	1.22	0.73				
col14a1a	collagen, type XIV, alpha 1a	ENSDARG00000005762	0.74	1.17	1.08	0.80				
col4a6	collagen, type IV, alpha 6	ENSDARG00000052061	0.72	1.49	1.35	0.80				
col1a1a	collagen, type I, alpha 1a	ENSDARG00000012405	0.72	1.01	1.07	0.68				
col4a5	collagen, type IV, alpha 5 (Alport syndrome)	ENSDARG00000052063	0.68	1.29	1.06	0.83				
col10a1a	collagen, type X, alpha 1a	ENSDARG00000054753	0.50	0.57	0.54	0.53				
col9a2	procollagen, type IX, alpha 2	ENSDARG00000024492	0.40	0.84	0.90	0.37				
col9a3	collagen, type IX, alpha 3	ENSDARG00000037845	0.40	0.97	1.17	0.33				
col9a1b	collagen, type IX, alpha 1b	ENSDARG00000031483	0.35	1.13	1.00	0.40				
col11a1a	collagen, type XI, alpha 1a	ENSDARG00000026165	0.33	1.05	0.76	0.46				
col2a1a	collagen, type II, alpha 1a	ENSDARG00000069093	0.32	1.77	1.51	0.37				
col11a2	collagen, type XI, alpha 2	ENSDARG00000012422	0.30	1.16	0.92	0.38				
col7a1	collagen, type VII, alpha 1	ENSDARG00000021720	0.21	2.36	0.84	0.60				
ctsl.1	cathepsin L.1	ENSDARG00000003902	11.91	0.91	1.28	8.44				
ctss2.1	cathepsin Sb, tandem duplicate 1	ENSDARG00000074656	6.91	2.06	2.15	6.61				
ctsk	cathepsin K	ENSDARG00000040251	4.58	0.82	1.46	2.58				
ctss2.2	cathepsin Sb, tandem duplicate 2	ENSDARG00000013771	3.58	1.46	3.70	1.42				
ctsh	cathepsin H	ENSDARG00000041108	2.99	0.86	1.41	1.83				
ctsba	cathepsin Ba	ENSDARG00000055120	2.29	1.16	2.44	1.09				
ctsc	cathepsin C	ENSDARG00000101334	2.27	0.90	1.01	2.01				
ctsz	cathepsin Z	ENSDARG00000043081	2.02	0.79	1.08	1.48				
ctsla	cathepsin La	ENSDARG00000007836	1.94	1.12	1.43	1.51				
ctsd	cathepsin D	ENSDARG00000057698	1.67	1.09	1.24	1.46				
ctss1	cathepsin Sa	ENSDARG00000036940	1.07	1.04	1.24	0.89				
ctsll	cathepsin L, like	ENSDARG00000011701	0.73	1.17	5.20	0.16				
ctsf	cathepsin F	ENSDARG00000063095	0.70	1.06	1.05	0.72				
cyba	cytochrome b-245, alpha polypeptide	ENSDARG00000018283	3.59	1.17	1.66	2.54				
cybb	cytochrome b-245, beta polypeptide (chronic	ENSDARG00000056615	3.51	0.58	1.17	1.74				
cyb5b	cytochrome b5 type B	ENSDARG00000099774	2.36	0.75	0.83	2.14				
cyb5d1	cytochrome b5 domain containing 1	ENSDARG00000056007	1.66	1.03	0.37	4.66				
cyb5r3	cytochrome b5 reductase 3	ENSDARG00000005891	1.65	1.11	1.74	1.05				
cyb5a	cytochrome b5 type A (microsomal)	ENSDARG00000098589	1.51	1.30	1.59	1.23				

Fold change
- >5.0 = red
- >3.0 = light red
- >1.2 = pale red
- <0.85 = pale blue
- <0.65 = light blue
- <0.5 = blue

Gene counts
- >1
- >10
- >100
- >1000

p<0.05 p<0.1

Gene Symbol	Gene Title	ensemble	(1) DMSO (operated vs unoperated)	(2) Operated (SB431542 vs DMSO)	(3) Unoperated (SB431542 vs DMSO)	(4) SB431542 (operated vs unoperated)	DMSO unoperated COUNT	DMSO operated COUNT	SB43 unoperated COUNT	SB43 operated COUNT
cyb5r1	cytochrome b5 reductase 1	ENSDARG00000018966	1.06	1.11	1.10	1.07				
dnajb9b		ENSDARG00000016886	3.27	1.16	0.50	7.50				
dnajc25		ENSDARG00000067613	2.75	4.37	1.00	12.25				
dnajc3a	DnaJ (Hsp40) homolog, subfamily C, member	ENSDARG00000041110	2.05	0.80	0.98	1.67				
dnajb11	DnaJ heat shock protein family (Hsp40)	ENSDARG00000015088	1.98	0.74	0.79	1.85				
dnajc12	DnaJ (Hsp40) homolog, subfamily C, member	ENSDARG00000086691	1.72	1.44	2.93	0.84				
dnajc5b		ENSDARG00000058147	1.65	1.14	1.64	1.14				
dnajb6b	DnaJ (Hsp40) homolog, subfamily B, member	ENSDARG00000020953	1.61	0.86	1.17	1.19				
dnajc9	DnaJ (Hsp40) homolog, subfamily C, member	ENSDARG00000031293	1.56	0.62	0.73	1.33				
dnajc16		ENSDARG00000059699	1.40	0.68	0.17	5.79				
dnaja1	DnaJ (Hsp40) homolog, subfamily A, member 1	ENSDARG00000030972	1.34	0.93	0.98	1.28				
dnajb12a	DnaJ (Hsp40) homolog, subfamily B, member	ENSDARG00000039363	1.33	1.03	1.73	0.80				
dnajc3b	DnaJ (Hsp40) homolog, subfamily C, member	ENSDARG00000017874	1.30	1.00	0.96	1.35				
dnajb1a	DnaJ (Hsp40) homolog, subfamily B, member	ENSDARG00000099383	1.28	0.96	1.03	1.19				
dnajc10	DnaJ (Hsp40) homolog, subfamily C, member	ENSDARG00000074727	1.26	0.76	0.76	1.26				
dnaja2b	DnaJ (Hsp40) homolog, subfamily A, member	ENSDARG00000010745	1.26	0.76	1.02	0.94				
dnajc8	DnaJ (Hsp40) homolog, subfamily C, member	ENSDARG00000059373	1.26	0.60	0.68	1.11				
dnajb1b	DnaJ (Hsp40) homolog, subfamily B, member	ENSDARG00000041394	1.24	6.57	4.66	1.75				
dnajc5gb	DnaJ (Hsp40) homolog, subfamily C, member	ENSDARG00000017687	1.22	2.53	2.48	1.25				
dnajc17	DnaJ (Hsp40) homolog, subfamily C, member	ENSDARG00000104959	1.16	0.55	0.63	1.01				
dnajb12b		ENSDARG00000087473	1.15	1.26	1.09	1.33				
dnajc21	DnaJ (Hsp40) homolog, subfamily C, member	ENSDARG00000105195	1.14	0.72	0.77	1.06				
dnajc16l		ENSDARG00000060725	1.12	1.12	1.47	0.86				
dnaja3a	DnaJ (Hsp40) homolog, subfamily A, member	ENSDARG00000058494	1.11	0.90	1.15	0.86				
dnaja2a	DnaJ (Hsp40) homolog, subfamily A, member 2	ENSDARG00000104066	1.09	0.82	0.86	1.04				
dnajc11a	DnaJ (Hsp40) homolog, subfamily C, member	ENSDARG00000011196	1.04	0.94	0.97	1.00				
dnajc11b		ENSDARG00000102696	1.02	1.08	1.01	1.09				
dnajb14		ENSDARG00000069996	1.00	0.88	1.31	0.68				
dnajc1	DnaJ (Hsp40) homolog, subfamily C, member	ENSDARG00000001940	0.99	0.94	0.91	1.02				
dnajb2		ENSDARG00000058644	0.96	1.69	2.52	0.65				
dnajc15		ENSDARG00000038309	0.92	1.38	1.39	0.92				
dnajc7	DnaJ (Hsp40) homolog, subfamily C, member	ENSDARG00000058148	0.89	0.93	0.89	0.93				
dnajc5ga	DnaJ (Hsp40) homolog, subfamily C, member	ENSDARG00000041896	0.89	0.71	0.87	0.72				
dnajc6		ENSDARG00000079891	0.88	1.02	1.14	0.79				
dnajc27	DnaJ (Hsp40) homolog, subfamily C, member	ENSDARG00000070916	0.84	0.95	1.23	0.65				
dnajc19	DnaJ (Hsp40) homolog, subfamily C, member	ENSDARG00000044420	0.83	0.78	0.89	0.73				
dnajc5ab	DnaJ (Hsp40) homolog, subfamily C, member	ENSDARG00000004836	0.83	0.82	0.80	0.85				
dnajc5aa		ENSDARG00000042948	0.82	1.15	1.14	0.82				
dnajb4		ENSDARG00000038978	0.80	1.35	1.16	0.93				
dnajc24	DnaJ (Hsp40) homolog, subfamily C, member	ENSDARG00000023927	0.79	0.68	0.59	0.89				
dnajc28		ENSDARG00000018181	0.78	1.30	1.19	0.86				
dnajc4		ENSDARG00000024090	0.78	1.44	1.38	0.81				
dnaja3b	DnaJ (Hsp40) homolog, subfamily A, member	ENSDARG00000102295	0.78	2.28	2.25	0.79				
dnajc2		ENSDARG00000070477	0.71	0.94	0.74	0.90				
dnajc14		ENSDARG00000105398	0.67	2.08	0.35	4.02				
dnajb6a	DnaJ (Hsp40) homolog, subfamily B, member	ENSDARG00000004680	0.67	2.48	2.72	0.61				
dnajb13		ENSDARG00000043157	0.66	1.38	0.81	1.14				
dnajc22		ENSDARG00000037067	0.57	2.10	0.49	2.44				
dnajc30b	DnaJ (Hsp40) homolog, subfamily C, member	ENSDARG00000079415	0.56	1.17	2.49	0.26				
dnajc18	DnaJ (Hsp40) homolog, subfamily C, member	ENSDARG00000056005	0.47	1.19	0.97	0.58				
dnajb1	DnaJ (Hsp40) homolog, subfamily B, member 1	ENSDARG00000015831								

Fold change
>5.0 = red
>3.0 = light red
>1.2 = pale red
<0.85 = pale blue
<0.65 = light blue
<0.5 = blue

Gene counts
>1
>10
>100
>1000

p<0.05 p<0.1

Gene Symbol	Gene Title	ensemble	(1) DMSO (operated vs unoperated)	(2) Operated (SB431542 vs DMSO)	(3) Unoperated (SB431542 vs DMSO)	(4) SB431542 (operated vs unoperated)
hoxc5a	homeobox C5a	ENSDARG00000070340	8.15	1.47	1.00	12.17
hoxa1a	homeobox A1a	ENSDARG00000104307	7.96	1.06	1.00	8.62
hoxa13b	homeobox A13b	ENSDARG00000036254	1.80	0.99	1.35	1.32
hoxd13a	homeobox D13a	ENSDARG00000059256	1.70	0.84	1.72	0.82
hoxa11a	homeobox A11a	ENSDARG00000104162	1.58	1.69	1.99	1.34
hoxb7a	homeobox B7a	ENSDARG00000056030	1.53	1.24	2.61	0.73
hoxd12a	homeobox D12a	ENSDARG00000059263	1.52	1.57	2.63	0.91
hoxc13a	homeobox C13a	ENSDARG00000070353	1.37	0.88	0.73	1.66
hoxb8b	homeobox B8b	ENSDARG00000054025	1.31	0.72	2.58	0.37
hoxb2a	homeobox B2a	ENSDARG00000000175	1.31	1.02	1.37	0.97
hoxb2a	homeobox B2a	ENSDARG00000000175	1.31	1.02	1.37	0.97
hoxb6b	homeobox B6b	ENSDARG00000026513	1.26	1.13	1.28	1.11
hoxa9b	homeobox A9b	ENSDARG00000056819	1.15	1.04	1.59	0.76
hoxa2b	homeobox A2b	ENSDARG00000023031	1.13	1.14	1.14	1.13
hoxa9a	homeobox A9a	ENSDARG00000105013	1.09	1.15	1.51	0.83
hoxb4a	homeobox B4a	ENSDARG00000013533	1.00	2.31	1.00	1.70
hoxc4a	homeobox C4a	ENSDARG00000070338	1.00	1.00	1.91	0.45
hoxa10b	homeobox A10b	ENSDARG00000031337	1.00	0.87	1.07	0.81
hoxd10a	homeobox D10a	ENSDARG00000057859	0.94	1.04	1.08	0.90
hoxc12b	homeobox C12b	ENSDARG00000103133	0.93	0.89	0.88	0.93
hoxc9a	homeobox C9a	ENSDARG00000092809	0.91	1.40	0.70	1.81
hoxb8a	homeobox B8a	ENSDARG00000056027	0.90	0.83	0.98	0.77
hoxb5b	homeobox B5b	ENSDARG00000054030	0.88	0.71	0.63	0.99
hoxb5a	homeobox B5a	ENSDARG00000013057	0.87	0.97	0.97	0.86
hoxc11b	homeobox C11b	ENSDARG00000102631	0.86	1.14	1.19	0.82
hoxd9a	homeobox D9a	ENSDARG00000059274	0.84	0.63	0.75	0.72
hoxb3a	homeobox B3a	ENSDARG00000029263	0.82	0.86	1.01	0.70
hoxd3a	homeobox D3a	ENSDARG00000059280	0.82	1.56	0.86	1.47
hoxb6a	homeobox B6a	ENSDARG00000010630	0.79	0.73	0.59	0.98
hoxa11b	homeobox A11b	ENSDARG00000007009	0.77	0.97	0.86	0.86
hoxa4a	homeobox A3a /// homeobox A4a	ENSDARG00000103862	0.72	0.94	0.88	0.76
hoxd11a	homeobox D11a	ENSDARG00000059267	0.70	0.95	1.28	0.52
hoxc6b	homeobox C6b	ENSDARG00000101954	0.67	1.23	0.86	0.97
hoxd4a	homeobox D4a	ENSDARG00000059276	0.67	1.18	0.94	0.84
hoxc10a	homeobox C10a	ENSDARG00000070348	0.65	0.94	0.83	0.74
hoxc6a	homeobox C6a	ENSDARG00000070343	0.59	1.20	0.59	1.19
hoxc8a	homeobox C8a	ENSDARG00000070346	0.49	1.16	0.46	1.24
hsp90b1	heat shock protein 90, beta (grp94), member 1	ENSDARG00000114206	11.57	0.87	1.04	9.60
hsp90b1	heat shock protein 90, beta (grp94), member 1	ENSDARG00000003570	2.00	0.95	1.02	1.86
hspa4a	heat shock protein 4a	ENSDARG00000004754	1.69	0.87	0.93	1.57
hspd1	heat shock 60 protein 1	ENSDARG00000056160	1.55	1.06	1.24	1.33
hspa4b	heat shock protein 4b	ENSDARG00000018989	1.50	0.69	0.87	1.18
hspa5	heat shock protein 5	ENSDARG00000103846	1.45	0.86	0.99	1.25
hspa5	heat shock protein 5	ENSDARG00000103846	1.45	0.86	0.99	1.25
hspb1	heat shock protein, alpha-crystallin-related, 1	ENSDARG00000041065	1.34	0.78	0.90	1.16
hsp90aa1.2	heat shock protein 90, alpha (cytosolic), class	ENSDARG00000024746	1.33	0.87	0.84	1.38
hspa14	heat shock protein 14	ENSDARG00000058030	1.23	0.75	0.88	1.05
hspe1	heat shock 10 protein 1	ENSDARG00000056167	1.17	0.91	0.99	1.08
hspa9	heat shock protein 9	ENSDARG00000003035	1.14	1.29	1.60	0.92
hspa8	heat shock protein 8	ENSDARG00000068992	1.11	0.94	0.93	1.13
hsp90ab1	heat shock protein 90, alpha (cytosolic), class	ENSDARG00000029150	0.91	0.72	0.66	0.99

DMSO unoperated COUNT
DMSO operated COUNT
SB43 unoperated COUNT
SB43 operated COUNT

Fold change
- >5.0 = red
- >3.0 = light red
- >1.2 = pale red
- <0.85 = pale blue
- <0.65 = light blue
- <0.5 = blue

Gene counts
- >1
- >10
- >100
- >1000

p<0.05 p<0.1

Gene Symbol	Gene Title	ensemble	(1) DMSO (operated vs unoperated)	(2) Operated (SB431542 vs DMSO)	(3) Unoperated (SB431542 vs DMSO)	(4) SB431542 (operated vs unoperated)	DMSO unoperated COUNT	DMSO operated COUNT	SB43 unoperated COUNT	SB43 operated COUNT
hspb6	heat shock protein, alpha-crystallin-related, b6	ENSDARG00000077236	0.91	1.55	1.44	0.97				
hspbp1	HSPA (heat shock 70kDa) binding protein,	ENSDARG00000102937	0.84	1.02	1.28	0.67				
hspb8	heat shock protein b8	ENSDARG00000058365	0.78	1.14	1.09	0.82				
hsp90aa1.1	heat shock protein 90, alpha (cytosolic), class	ENSDARG00000010478	0.74	0.91	1.02	0.66				
hspa4l	heat shock protein 4 like	ENSDARG00000053544	0.70	1.11	0.90	0.86				
hspa12a	heat shock protein 12A	ENSDARG00000070603	0.65	0.98	1.06	0.60				
hspb15	heat shock protein, alpha-crystallin-related, b15	ENSDARG00000078411	0.57	1.20	0.87	0.79				
igfbp5a		ENSDARG00000039264	2.75	0.12	1.52	0.22				
igfbp1a	insulin-like growth factor binding protein 1a	ENSDARG00000099351	2.01	1.59	1.27	2.53				
igfbp1b	insulin-like growth factor binding protein 1b	ENSDARG00000038666	1.36	5.17	1.21	5.85				
igfbp6a		ENSDARG00000070941	1.15	0.41	1.60	0.29				
igfbp7		ENSDARG00000104138	1.00	2.31	1.91	0.87				
igfbp6b	insulin-like growth factor binding protein 6b	ENSDARG00000090833	0.90	0.50	0.63	0.71				
igfbp5b	insulin-like growth factor binding protein 5b	ENSDARG00000025348	0.87	1.89	1.72	0.96				
igfbp2b		ENSDARG00000031422	0.85	0.68	0.13	4.62				
igfbp3	insulin-like growth factor binding protein 3	ENSDARG00000099144	0.82	3.38	3.79	0.73				
igfbp2a	insulin-like growth factor binding protein 2a	ENSDARG00000052470	0.75	0.80	1.09	0.55				
krt93	xkeratin 93	ENSDARG00000044976	5.95	0.14	1.00	1.00				
krt18a.1	keratin 18	ENSDARG00000018404	5.05	0.75	1.18	3.20				
krt96	keratin 96	ENSDARG00000095147	2.86	0.19	0.92	0.59				
krt97	keratin 97	ENSDARG00000000212	2.66	0.30	0.74	1.08				
krt94	keratin 94	ENSDARG00000044975	1.93	0.64	0.54	2.31				
krt18b	keratin zgc:77517 krtt1c6	ENSDARG00000028618	1.91	0.86	0.93	1.76				
krt8	keratin 8	ENSDARG00000058358	1.67	0.75	0.95	1.32				
krt1-c5	keratin 1	ENSDARG00000026979	1.22	0.71	0.74	1.17				
krt222	keratin 222	ENSDARG00000071518	1.18	0.90	1.25	0.85				
krt99	keratin 99 zgc:110712	ENSDARG00000019365	0.94	0.58	1.41	0.39				
krt98	keratin 98	ENSDARG00000044973	0.71	0.31	0.66	0.33				
krt5	keratin 5	ENSDARG00000058371	0.56	1.44	1.30	0.62				
krt92	keratin 96?	ENSDARG00000036834	0.53	2.05	1.01	1.08				
krt4	keratin 4	ENSDARG00000017624	0.49	1.33	0.99	0.66				
krt1-19e	keratin type 1 19e	ENSDARG00000090268	0.47	0.96	0.90	0.51				
krt91	keratin 91	ENSDARG00000036830	0.47	1.19	0.83	0.67				
krt17	keratin 17	ENSDARG00000094041	0.46	1.24	0.85	0.68				
krt15	keratin 15	ENSDARG00000036840	0.36	1.35	0.65	0.74				
krt1-19d	keratin, type 1, gene 19d	ENSDARG00000023082	0.23	1.97	0.82	0.55				
map1lc3c	microtubule-associated protein 1 light chain 3	ENSDARG00000100528	6.03	0.14	0.25	3.45				
mapre1b	microtubule-associated protein, RP/EB family,	ENSDARG00000002659	1.67	0.67	0.91	1.24				
map1lc3a	microtubule-associated protein 1 light chain 3	ENSDARG00000033609	1.37	1.30	1.89	0.94				
mapre1a	microtubule-associated protein, RP/EB family,	ENSDARG00000042927	1.14	1.11	1.11	1.14				
map1lc3b	microtubule-associated protein 1 light chain 3	ENSDARG00000101127	1.02	1.01	1.00	1.03				
map9	microtubule-associated protein 9	ENSDARG00000037276	1.02	1.08	0.65	1.68				
map1sa	microtubule-associated protein 1Sa	ENSDARG00000060805	0.79	0.86	0.84	0.81				
maptb	microtubule-associated protein tau b	ENSDARG00000087616	0.73	0.58	0.73	0.58				
map1ab	microtubule-associated protein 1Ab	ENSDARG00000022045	0.62	0.94	0.84	0.69				
mapre3b	microtubule-associated protein, RP/EB family,	ENSDARG00000102878	0.58	1.01	0.93	0.64				
map1lc3cl	microtubule-associated protein 1 light chain 3	ENSDARG00000075727	0.29	0.82	1.08	0.22				
mmp13b	matrix metallopeptidase 13b	ENSDARG00000100794	78.68	0.94	6.93	10.68				
mmp9	matrix metallopeptidase 9	ENSDARG00000042816	10.35	2.42	3.37	7.44				

Fold change
- >5.0 = red
- >3.0 = light red
- >1.2 = pale red
- <0.85 = pale blue
- <0.65 = light blue
- <0.5 = blue

Gene counts
- >1
- >10
- >100
- >1000

p<0.05 p<0.1

Gene Symbol	Gene Title	ensemble	(1) DMSO (operated vs unoperated)	(2) Operated (SB431542 vs DMSO)	(3) Unoperated (SB431542 vs DMSO)	(4) SB431542 (operated vs unoperated)	DMSO unoperated COUNT	DMSO operated COUNT	SB43 unoperated COUNT	SB43 operated COUNT
mmp13a	matrix metallopeptidase 13a	ENSDARG00000012395	7.49 (p<0.05)	1.01	1.20	6.30 (p<0.05)	>10	>100	>10	>100
mmp13a	matrix metallopeptidase 13a	ENSDARG00000114451	4.34 (p<0.05)	1.91 (p<0.1)	1.53	5.42 (p<0.05)	>10	>10	>10	>100
mmp14b	matrix metallopeptidase 14b (membrane-	ENSDARG00000008388	2.01 (p<0.05)	0.94	1.88 (p<0.05)	1.01	>100	>100	>100	>100
mmp2	matrix metallopeptidase 2	ENSDARG00000017676	1.64	1.61	3.05 (p<0.05)	0.87	>10	>100	>100	>100
mmp14a	matrix metallopeptidase 14a (membrane-	ENSDARG00000002235	1.27	0.96	1.17	1.04	>100	>100	>100	>100
MMP23B	matrix metallopeptidase 23B	ENSDARG00000043079	1.00	3.92	1.00	2.89	–	–	–	–
mmp17b	matrix metallopeptidase 17b	ENSDARG00000102956	0.88	1.28	1.18	0.96	>10	>10	>100	>10
mmp30	matrix metallopeptidase 30	ENSDARG00000045887	0.62 (p<0.1)	2.44 (p<0.05)	1.22	1.25	>100	>100	>100	>100
ms4a17a.4	membrane-spanning 4-domains	ENSDARG00000014024	9.71 (p<0.05)	0.70	1.04	6.57 (p<0.05)	>10	>100	>10	>100
ms4a17a.5	membrane-spanning 4-domains	ENSDARG00000092204	6.24 (p<0.05)	0.67	1.30	3.21 (p<0.05)	>10	>10	>10	>10
ms4a17a.2	membrane-spanning 4-domains	ENSDARG00000105674	5.82 (p<0.05)	0.70	0.96	4.23 (p<0.05)	>1	>10	>1	>10
ms4a17a.7	membrane-spanning 4-domains	ENSDARG00000043796	5.36 (p<0.05)	0.81	1.65	2.64 (p<0.05)	>10	>100	>10	>100
ms4a17a.1	membrane-spanning 4-domains, subfamily A,	ENSDARG00000043798	4.40 (p<0.05)	0.57	0.48	5.14 (p<0.05)	>10	>10	>10	>10
ms4a17a.12	membrane-spanning 4-domains	ENSDARG00000053563	2.74 (p<0.05)	0.86	1.04	2.27 (p<0.05)	>10	>100	>10	>10
ms4a17a.11	membrane-spanning 4-domains	ENSDARG00000094809	2.71 (p<0.05)	0.88	0.86	2.77 (p<0.05)	>10	>100	>10	>100
ms4a17a.9	membrane-spanning 4-domains	ENSDARG00000094854	2.32 (p<0.05)	0.87	1.23	1.64	>100	>100	>100	>100
mybphb	myosin binding protein Hb	ENSDARG00000003081	0.99	2.04 (p<0.05)	2.21 (p<0.05)	0.92	>1000	>1000	>1000	>1000
mybpc2b	myosin binding protein C, fast type b	ENSDARG00000021265	0.88	0.66	0.77	0.75	>100	>100	>100	>100
mybpc3	myosin binding protein C, cardiac	ENSDARG00000011615	0.63 (p<0.05)	0.98	1.02	0.61 (p<0.05)	>1000	>1000	>1000	>1000
mybpc1	myosin binding protein C1	ENSDARG00000045560	0.52 (p<0.05)	0.71	0.82	0.45 (p<0.05)	>100	>100	>100	>100
mybpha	myosin binding protein Ha	ENSDARG00000058799	0.33 (p<0.05)	1.33	0.99	0.44 (p<0.1)	>10	>10	>10	>10
myl12.1	myosin, light chain 12, genome duplicate 1	ENSDARG00000099766	1.56 (p<0.1)	1.18	1.55	1.19	>1000	>1000	>1000	>1000
mylipa	myosin regulatory light chain interacting protein	ENSDARG00000008859	1.01	1.45	1.45	1.01	>10	>10	>10	>10
myl7	myosin, light chain 7, regulatory	ENSDARG00000019096	1.00	2.31	1.91	0.87	–	–	–	–
myl12.2	myosin, light chain 12, genome duplicate 2	ENSDARG00000025326	0.99	0.84	0.95	0.87	>100	>100	>100	>100
myl6	myosin, light chain 6, alkali, smooth muscle	ENSDARG00000008494	0.86	0.92	0.87	0.91	>100	>100	>100	>100
myl10	myosin, light chain 10, regulatory	ENSDARG00000062592	0.77	0.75	0.91	0.64 (p<0.05)	>1000	>1000	>1000	>1000
myl1	myosin, light chain 1, alkali; skeletal, fast	ENSDARG00000014196	0.73	0.80	0.99	0.59 (p<0.05)	>1000	>1000	>1000	>1000
mylpfb	myosin light chain, phosphorylatable, fast	ENSDARG00000002589	0.72	1.06	1.25	0.61 (p<0.05)	>1000	>1000	>1000	>1000
myl13	myosin, light chain 13	ENSDARG00000042245	0.69 (p<0.05)	1.07	1.02	0.72	>1000	>1000	>1000	>1000
mylpfa	myosin light chain, phosphorylatable, fast	ENSDARG00000053254	0.62 (p<0.05)	1.22	1.06	0.72	>1000	>1000	>1000	>1000
mylz3	myosin, light polypeptide 3, skeletal muscle	ENSDARG00000017441	0.61 (p<0.05)	0.99	0.94	0.64 (p<0.05)	>1000	>1000	>1000	>1000
mylk4a	myosin light chain kinase 3-like	ENSDARG00000091260	0.54	2.73	0.76	1.93	>10	>10	>10	>10
ndufs1	NADH dehydrogenase (ubiquinone) Fe-S	ENSDARG00000028546	4.06	0.21	1.00	1.00	–	–	–	–
ndufa4	NADH dehydrogenase (ubiquinone) 1 alpha	ENSDARG00000056108	1.03	2.49 (p<0.05)	3.37 (p<0.05)	0.76	>100	>100	>100	>100
ndufaf4	NADH dehydrogenase (ubiquinone) complex I,	ENSDARG00000077859	0.97	1.16	1.29	0.88	>100	>100	>100	>100
ndufs8a	NADH dehydrogenase (ubiquinone) Fe-S	ENSDARG00000051986	0.93	1.03	1.14	0.84	>1000	>1000	>1000	>1000
ndufs5	NADH dehydrogenase (ubiquinone) Fe-S	ENSDARG00000006290	0.90	0.80	0.88	0.82	>1000	>1000	>1000	>1000
ndufb4	NADH dehydrogenase (ubiquinone) 1 beta	ENSDARG00000019332	0.86	1.36	1.36	0.87	>1000	>1000	>1000	>1000
ndufb8	NADH dehydrogenase (ubiquinone) 1 beta	ENSDARG00000010113	0.86	0.81	0.98	0.71	>1000	>1000	>1000	>1000
ndufb9	NADH dehydrogenase (ubiquinone) 1 beta	ENSDARG00000041314	0.85	0.82	0.91	0.76	>1000	>1000	>1000	>1000
ndufv2	NADH dehydrogenase (ubiquinone)	ENSDARG00000013044	0.84	0.90	1.31	0.58 (p<0.05)	>100	>100	>100	>100
ndufc2	NADH dehydrogenase (ubiquinone) 1,	ENSDARG00000102115	0.84	0.94	1.05	0.75 (p<0.05)	>1000	>1000	>1000	>1000
ndufa6	NADH dehydrogenase (ubiquinone) 1 alpha	ENSDARG00000038028	0.82	0.73	0.75	0.80	>1000	>1000	>1000	>1000
ndufb10	NADH dehydrogenase (ubiquinone) 1 beta	ENSDARG00000028889	0.82	0.96	0.99	0.79	>1000	>1000	>1000	>1000
ndufaf1	NADH dehydrogenase (ubiquinone) complex I,	ENSDARG00000025549	0.80	1.66 (p<0.05)	1.47	0.90	>100	>100	>100	>100
ndufab1a	NADH dehydrogenase (ubiquinone) 1,	ENSDARG00000058463	0.79	1.19	1.10	0.85	>1000	>1000	>1000	>1000
ndufv1	NADH dehydrogenase (ubiquinone)	ENSDARG00000036438	0.78	1.12	1.07	0.82	>1000	>1000	>1000	>1000
ndufs2	NADH dehydrogenase (ubiquinone) Fe-S	ENSDARG00000007526	0.78	1.03	1.04	0.77	>1000	>1000	>1000	>1000

Fold change
>5.0 = red
>3.0 = light red
>1.2 = pale red
<0.85 = pale blue
<0.65 = light blue
<0.5 = blue

Gene counts
>1
>10
>100
>1000

p<0.05 p<0.1

Gene Symbol	Gene Title	ensemble	(1) DMSO (operated vs unoperated)	(2) Operated (SB431542 vs DMSO)	(3) Unoperated (SB431542 vs DMSO)	(4) SB431542 (operated vs unoperated)	DMSO unoperated COUNT	DMSO operated COUNT	SB43 unoperated COUNT	SB43 operated COUNT
ndufb5	NADH dehydrogenase (ubiquinone) 1 beta	ENSDARG00000070824	0.76	1.13	1.26	0.69				
ndufaf6	NADH dehydrogenase (ubiquinone) complex I,	ENSDARG00000053652	0.75	0.85	0.69	0.92				
ndufb6	NADH dehydrogenase (ubiquinone) 1 beta	ENSDARG00000037259	0.75	1.12	1.10	0.76				
ndufs6	NADH dehydrogenase (ubiquinone) Fe-S	ENSDARG00000056583	0.75	1.46	2.00	0.54				
ndufa8	NADH dehydrogenase (ubiquinone) 1 alpha	ENSDARG00000058041	0.73	1.19	1.22	0.71				
ndufb3	NADH dehydrogenase (ubiquinone) 1 beta	ENSDARG00000075709	0.72	0.86	0.89	0.70				
ndufb7	NADH dehydrogenase (ubiquinone) 1 beta	ENSDARG00000033789	0.71	0.85	0.88	0.69				
ndufs4	NADH dehydrogenase (ubiquinone) Fe-S	ENSDARG00000052840	0.71	0.89	0.85	0.74				
ndufa10	NADH dehydrogenase (ubiquinone) 1 alpha	ENSDARG00000013333	0.69	0.94	0.82	0.80				
ndufa4l	NADH dehydrogenase (ubiquinone) 1 alpha	ENSDARG00000099499	0.68	1.13	1.03	0.74				
NDUFB1	NADH dehydrogenase (ubiquinone) 1 beta	ENSDARG00000087456	0.67	1.05	0.87	0.81				
ndufab1b	NADH dehydrogenase (ubiquinone) 1,	ENSDARG00000014915	0.66	0.99	0.95	0.69				
ndufa5	NADH dehydrogenase (ubiquinone) 1 alpha	ENSDARG00000039346	0.65	1.16	0.96	0.79				
ndufb2	NADH dehydrogenase (ubiquinone) 1 beta	ENSDARG00000045490	0.65	1.01	0.89	0.74				
ndufv3	NADH:ubiquinone oxidoreductase subunit V3	ENSDARG00000090389	0.64	0.89	0.84	0.68				
ndufaf5	NADH dehydrogenase (ubiquinone) complex I,	ENSDARG00000061629	0.64	0.90	0.77	0.75				
ndufa2	NADH dehydrogenase (ubiquinone) 1 alpha	ENSDARG00000021984	0.62	0.90	0.75	0.75				
ndufa11	NADH dehydrogenase (ubiquinone) 1 alpha	ENSDARG00000042777	0.62	1.01	0.89	0.70				
ndufs7	NADH dehydrogenase (ubiquinone) Fe-S	ENSDARG00000074552	0.54	1.06	0.77	0.75				
ndufa1	NADH dehydrogenase (ubiquinone) 1 alpha	ENSDARG00000036329	0.52	1.10	0.80	0.71				
nr5a5	nuclear receptor subfamily 5, group A, member	ENSDARG00000039116	1.60	6.65	2.67	3.98				
nr5a1b	nuclear receptor subfamily 5, group A, member	ENSDARG00000023362	1.41	2.32	4.27	0.76				
nr1h3	nuclear receptor subfamily 1, group H, member	ENSDARG00000098439	1.34	1.06	1.26	1.13				
nrip1b	nuclear receptor interacting protein 1b	ENSDARG00000068894	1.30	1.47	2.00	0.95				
nrf1	nuclear respiratory factor 1	ENSDARG00000000018	1.22	0.89	1.18	0.93				
nr2f6a	nuclear receptor subfamily 2, group F, member	ENSDARG00000003607	1.19	0.69	0.96	0.85				
nr2f1a	nuclear receptor subfamily 2, group F, member	ENSDARG00000052695	1.11	0.76	1.03	0.83				
nrbf2b	nuclear receptor binding factor 2b	ENSDARG00000023591	1.05	1.10	1.14	1.01				
nr2f5	nuclear receptor subfamily 2, group F, member	ENSDARG00000033172	1.01	1.61	1.34	1.22				
nr2e1	nuclear receptor subfamily 2, group E, member	ENSDARG00000017107	1.00	1.00	3.04	0.28				
nr2f6b	nuclear receptor subfamily 2, group F, member	ENSDARG00000003165	0.98	1.04	0.98	1.04				
nr1h5	nuclear receptor subfamily 1, group H, member	ENSDARG00000031046	0.91	2.98	3.21	0.84				
nr3c1	nuclear receptor subfamily 3, group C, member	ENSDARG00000025032	0.85	0.87	0.85	0.86				
nr2f2	nuclear receptor subfamily 2, group F, member	ENSDARG00000040926	0.78	1.08	1.08	0.78				
nr4a2b	nuclear receptor subfamily 4, group A, member	ENSDARG00000044532	0.67	1.63	1.13	0.97				
nr2f1b	nuclear receptor subfamily 2, group F, member	ENSDARG00000017168	0.67	1.11	0.67	1.10				
nr1d2b	nuclear receptor subfamily 1, group D, member	ENSDARG00000009594	0.64	1.23	1.94	0.41				
nr1d2a	nuclear receptor subfamily 1, group D, member	ENSDARG00000003820	0.62	1.39	1.15	0.75				
nr0b2a	nuclear receptor subfamily 0, group B, member	ENSDARG00000044685	0.61	2.92	2.29	0.77				
nr2c2	nuclear receptor subfamily 2, group C, member	ENSDARG00000042477	0.53	0.97	0.93	0.55				
nr4a1	nuclear receptor subfamily 4, group A, member	ENSDARG00000000796	0.52	3.44	2.55	0.70				
nr1d1	nuclear receptor subfamily 1, group d, member	ENSDARG00000033160	0.49	0.92	1.41	0.31				
nr6a1a	nuclear receptor subfamily 6, group A, member	ENSDARG00000101508	0.47	0.61	0.33	1.00				
nr5a2	nuclear receptor subfamily 5, group A, member	ENSDARG00000100940	0.42	1.11	0.50	0.95				
nr5a1a	nuclear receptor subfamily 5, group A, member	ENSDARG00000103176	0.24	3.76	0.77	1.16				
ppp1r18	protein phosphatase 1, regulatory subunit 18	ENSDARG00000071251	4.57	0.55	1.16	2.15				
ppp1r11	protein phosphatase 1, regulatory (inhibitor)	ENSDARG00000036063	4.50	0.72	4.57	0.71				
ppp2r2ab	protein phosphatase 2, regulatory subunit B,	ENSDARG00000006624	2.48	1.81	1.56	2.88				
ppp2r2ab	protein phosphatase 2, regulatory subunit B,	ENSDARG00000006624	2.48	1.81	1.56	2.88				
ppp4r2b	protein phosphatase 4, regulatory subunit 2b	ENSDARG00000053447	2.15	0.79	1.56	1.08				
ppp1r3ca	protein phosphatase 1, regulatory subunit 3Ca	ENSDARG00000071005	2.05	1.35	0.80	3.46				

Fold change
- >5.0 = red
- >3.0 = light red
- >1.2 = pale red
- <0.85 = pale blue
- <0.65 = light blue
- <0.5 = blue

Gene counts
- >1
- >10
- >100
- >1000

p<0.05 p<0.1

Gene Symbol	Gene Title	ensemble	(1) DMSO (operated vs unoperated)	(2) Operated (SB431542 vs DMSO)	(3) Unoperated (SB431542 vs DMSO)	(4) SB431542 (operated vs unoperated)	DMSO unoperated COUNT	DMSO operated COUNT	SB43 unoperated COUNT	SB43 operated COUNT
ppp1r3da	protein phosphatase 1, regulatory subunit 3Da	ENSDARG00000077513	2.00	0.95	1.69	1.12				
ppp4ca	protein phosphatase 4, catalytic subunit a	ENSDARG00000070570	1.86	0.91	1.54	1.10				
ppp4r2a	protein phosphatase 4, regulatory subunit 2a	ENSDARG00000026540	1.75	0.77	1.23	1.10				
ppp1caa	protein phosphatase 1, catalytic subunit, alpha	ENSDARG00000003486	1.48	1.43	1.94	1.09				
ppp1r15a	protein phosphatase 1, regulatory subunit 15A	ENSDARG00000069135	1.42	2.59	1.58	2.33				
ppp1cab	protein phosphatase 1, catalytic subunit, alpha	ENSDARG00000071566	1.41	0.82	1.02	1.13				
ppp1r8b	protein phosphatase 1, regulatory subunit 8b	ENSDARG00000022430	1.37	0.80	0.87	1.25				
ppp1r14bb	protein phosphatase 1, regulatory (inhibitor)	ENSDARG00000030161	1.36	1.06	1.67	0.87				
ppp1r3b	protein phosphatase 1, regulatory subunit 3B	ENSDARG00000044691	1.27	0.44	0.98	0.57				
ppp2cb	protein phosphatase 2, catalytic subunit, beta	ENSDARG00000099241	1.18	0.85	0.80	1.25				
ppp2r1ba	protein phosphatase 2, regulatory subunit A,	ENSDARG00000007791	1.16	0.70	0.77	1.05				
ppp4r3b	protein phosphatase 4, regulatory subunit 3B	ENSDARG00000103415	1.16	0.63	0.50	1.45				
ppp1r37	protein phosphatase 1, regulatory subunit 37	ENSDARG00000078458	1.13	1.57	1.34	1.32				
ppp1r13ba	protein phosphatase 1, regulatory subunit 13Ba	ENSDARG00000004377	1.11	0.99	1.33	0.82				
ppp1r13l	protein phosphatase 1, regulatory subunit 13	ENSDARG00000013777	1.09	0.82	0.74	1.21				
ppp6r3	protein phosphatase 6, regulatory subunit 3	ENSDARG00000013379	1.06	1.06	1.02	1.11				
ppp1r9bb	neurabin-2-like /// protein phosphatase 1,	ENSDARG00000071709	1.04	0.65	0.99	0.69				
ppp2r3c	protein phosphatase 2, regulatory subunit B'',	ENSDARG00000043972	1.04	1.38	1.53	0.93				
ppp6r2a	protein phosphatase 6, regulatory subunit 2a	ENSDARG00000045540	1.03	1.14	1.12	1.04				
ppp2r5d	protein phosphatase 2, regulatory subunit B',	ENSDARG00000014428	1.03	0.96	0.98	1.01				
ppp5c	protein phosphatase 5, catalytic subunit	ENSDARG00000034313	1.02	0.76	0.80	0.96				
ppp1r2	protein phosphatase 1, regulatory (inhibitor)	ENSDARG00000054007	0.98	1.71	1.40	1.20				
ppp1cb	protein phosphatase 1, catalytic subunit, beta	ENSDARG00000044153	0.98	1.05	1.16	0.88				
ppp1r12a	protein phosphatase 1, regulatory subunit 12A	ENSDARG00000010784	0.98	1.46	1.27	1.13				
ppp2r1bb	protein phosphatase 2, regulatory subunit A,	ENSDARG00000032430	0.97	0.87	0.85	0.99				
ppp2r5eb	protein phosphatase 2, regulatory subunit B',	ENSDARG00000069118	0.96	1.45	1.78	0.78				
ppp2r3b	protein phosphatase 2, regulatory subunit B'',	ENSDARG00000076004	0.89	1.27	1.09	1.04				
ppp1r7	protein phosphatase 1, regulatory (inhibitor)	ENSDARG00000009740	0.89	0.84	0.84	0.89				
ppp1r10	protein phosphatase 1, regulatory subunit 10	ENSDARG00000032651	0.89	1.20	1.01	1.06				
ppp3r1b	protein phosphatase 3 (formerly 2B), regulatory	ENSDARG00000069360	0.88	0.81	1.00	0.71				
ppp1r14ab	protein phosphatase 1, regulatory (inhibitor)	ENSDARG00000017710	0.86	1.02	0.96	0.91				
ppp1r14aa	protein phosphatase 1, regulatory (inhibitor)	ENSDARG00000011239	0.82	0.94	1.17	0.66				
ppp1r42	protein phosphatase 1, regulatory subunit 42	ENSDARG00000057632	0.80	0.70	1.03	0.54				
ppp3cb	protein phosphatase 3, catalytic subunit, beta	ENSDARG00000025106	0.79	1.00	1.09	0.72				
ppp6c	protein phosphatase 6, catalytic subunit	ENSDARG00000002949	0.78	2.39	1.78	1.05				
ppp2r5ea	protein phosphatase 2, regulatory subunit B',	ENSDARG00000015474	0.77	1.13	0.98	0.88				
ppp4r4	protein phosphatase 4, regulatory subunit 4	ENSDARG00000010407	0.74	2.05	1.46	1.04				
ppp1r13bb	protein phosphatase 1, regulatory subunit 13Bb	ENSDARG00000009142	0.71	0.92	0.70	0.94				
ppp2r2ca	protein phosphatase 2, regulatory subunit B,	ENSDARG00000056797	0.71	0.87	0.55	1.12				
ppp1r15b	protein phosphatase 1, regulatory subunit 15B	ENSDARG00000068128	0.69	2.17	0.90	1.67				
ppp3cca	protein phosphatase 3, catalytic subunit,	ENSDARG00000014962	0.66	0.90	0.64	0.94				
ppp2r5b	protein phosphatase 2, regulatory subunit B',	ENSDARG00000054931	0.61	1.79	0.98	1.11				
ppp3ca	protein phosphatase 3, catalytic subunit, alpha	ENSDARG00000004988	0.55	1.40	1.01	0.76				
ppp1r3ab	protein phosphatase 1, regulatory subunit 3Ab	ENSDARG00000088813	0.54	1.52	0.86	0.96				
ppp2r5ca	protein phosphatase 2, regulatory subunit B',	ENSDARG00000059083	0.50	2.93	0.50	2.90				
ppp1r1b	protein phosphatase 1, regulatory (inhibitor)	ENSDARG00000076280	0.46	0.93	0.58	0.73				
ppp1r3cb	protein phosphatase 1, regulatory subunit 3Cb	ENSDARG00000014554	0.42	1.18	0.72	0.69				
pvalb8	parvalbumin 8	ENSDARG00000037790	0.94	0.82	1.00	0.78				
pvalb2	parvalbumin 2	ENSDARG00000002768	0.69	1.41	1.40	0.69				
pvalb4	parvalbumin 4	ENSDARG00000024433	0.66	0.99	0.99	0.66				
pvalb5	parvalbumin 5	ENSDARG00000032836	0.66	1.01	0.91	0.73				
pvalb9	parvalbumin 9	ENSDARG00000071601	0.59	0.54	0.82	0.39				

Fold change
>5.0 = red
>3.0 = light red
>1.2 = pale red
<0.85 = pale blue
<0.65 = light blue
<0.5 = blue

p<0.05 p<0.1

Gene counts
>1
>10
>100
>1000

Gene Symbol	Gene Title	ensemble	(1) DMSO (operated vs unoperated)	(2) Operated (SB431542 vs DMSO)	(3) Unoperated (SB431542 vs DMSO)	(4) SB431542 (operated vs unoperated)	DMSO unoperated COUNT	DMSO operated COUNT	SB43 unoperated COUNT	SB43 operated COUNT
pvalb6	parvalbumin 6	ENSDARG00000009311	0.59	1.12	0.99	0.67				
pvalb3	parvalbumin 3	ENSDARG00000022817	0.54	1.10	0.97	0.61				
pvalb7	parvalbumin 7	ENSDARG00000034705	0.52	0.86	0.88	0.51				
pvalb1	parvalbumin 1	ENSDARG00000037789	0.49	1.08	0.79	0.67				
slc7a7	solute carrier family 7 (amino acid transporter	ENSDARG00000055226	7.70	0.67	1.00	5.15				
slc46a1	solute carrier family 46 (folate transporter),	ENSDARG00000026149	6.06	0.87	7.83	0.67				
slc22a7b.1	solute carrier family 22 (organic anion	ENSDARG00000056643	2.85	0.15	1.57	0.28				
slc39a7	solute carrier family 39 (zinc transporter),	ENSDARG00000104451	2.46	0.59	0.95	1.53				
slc12a8	solute carrier family 12, member 8	ENSDARG00000074384	2.16	1.15	1.58	1.57				
slc30a1a	solute carrier family 30 (zinc transporter),	ENSDARG00000005463	2.13	0.83	1.17	1.52				
slc51a	solute carrier family 51 member A	ENSDARG00000045306	2.05	0.82	0.80	2.10				
slc13a2	solute carrier family 13 (sodium-dependent	ENSDARG00000053853	1.98	2.55	2.32	2.17				
slc52a3	solute carrier family 52 (riboflavin transporter),	ENSDARG00000042737	1.97	1.51	1.39	2.15				
slc46a3	solute carrier family 46, member 3	ENSDARG00000077313	1.96	1.48	2.10	1.39				
slco1d1	solute carrier organic anion transporter family,	ENSDARG00000104108	1.95	0.68	1.50	0.88				
slco2b1	solute carrier organic anion transporter family,	ENSDARG00000054609	1.92	1.29	1.20	2.07				
slc25a19	solute carrier family 25 (mitochondrial thiamine	ENSDARG00000100385	1.89	1.76	4.65	0.71				
slc10a2	solute carrier family 10 (sodium/bile acid	ENSDARG00000014916	1.87	1.86	2.63	1.32				
slc2a10	solute carrier family 2 (facilitated glucose	ENSDARG00000090820	1.82	0.54	0.69	1.43				
slc24a2	solute carrier family 24	ENSDARG00000042988	1.79	1.71	1.58	1.95				
slc29a3	solute carrier family 29 (equilibrative nucleoside	ENSDARG00000077828	1.77	0.92	1.05	1.55				
slc10a7	solute carrier family 10, member 7	ENSDARG00000104508	1.74	0.69	0.95	1.27				
slc12a9	solute carrier family 12, member 9	ENSDARG00000060366	1.74	0.90	1.48	1.05				
slc20a1a	solute carrier family 20, member 1a	ENSDARG00000020114	1.73	1.10	1.80	1.06				
slc14a2	solute carrier family 14 member 2	ENSDARG00000051914	1.70	2.45	5.38	0.78				
slc9a3r1a	solute carrier family 9, subfamily A (NHE3,	ENSDARG00000000068	1.67	0.57	1.04	0.92				
slc35f6	solute carrier family 35, member F6	ENSDARG00000016745	1.66	0.85	0.94	1.52				
slc26a1	solute carrier family 26 (anion exchanger),	ENSDARG00000029832	1.65	0.47	1.07	0.73				
slc12a4	solute carrier family 12 (potassium/chloride	ENSDARG00000014378	1.64	1.52	1.94	1.29				
slc43a3b	solute carrier family 43 member 3b	ENSDARG00000057949	1.61	1.40	1.12	2.02				
slc52a2	solute carrier family 52 (riboflavin transporter),	ENSDARG00000102035	1.61	0.07	0.76	0.14				
slc4a1ap	solute carrier family 4 (anion exchanger),	ENSDARG00000034160	1.60	0.81	1.02	1.26				
slc2a11b	solute carrier family 2 (facilitated glucose	ENSDARG00000063288	1.59	0.79	1.59	0.79				
slc4a4b	solute carrier family 4 (sodium bicarbonate	ENSDARG00000044808	1.54	0.33	0.33	1.53				
slc26a11	solute carrier family 26 (anion exchanger),	ENSDARG00000043021	1.48	0.71	0.78	1.34				
slc12a3	solute carrier family 12 (sodium/chloride	ENSDARG00000013855	1.46	1.83	1.45	1.84				
slc39a9	solute carrier family 39, member 9	ENSDARG00000070447	1.46	0.87	0.59	2.14				
slc35f2	solute carrier family 35, member F2	ENSDARG00000069745	1.45	0.79	1.00	1.14				
slc31a1	solute carrier family 31 (copper transporter),	ENSDARG00000013961	1.41	0.65	0.78	1.18				
slc35b2	solute carrier family 35 (adenosine 3'-phospho	ENSDARG00000007886	1.41	1.47	2.30	0.90				
slc22a31	solute carrier family 22, member 31	ENSDARG00000078882	1.40	0.77	1.12	0.97				
slc30a7	solute carrier family 30 (zinc transporter),	ENSDARG00000019998	1.39	0.80	0.97	1.14				
slc35d1a	solute carrier family 35 (UDP-GlcA/UDP-	ENSDARG00000011973	1.36	0.89	1.44	0.84				
slc38a2	solute carrier family 38, member 2	ENSDARG00000045886	1.34	0.57	0.87	0.89				
slc17a9b	solute carrier family 17 (vesicular nucleotide	ENSDARG00000011049	1.33	0.85	0.61	1.84				
slc38a7	solute carrier family 38, member 7	ENSDARG00000012002	1.26	0.87	0.86	1.27				
slc25a33	solute carrier family 25 (pyrimidine nucleotide	ENSDARG00000039931	1.23	1.34	2.08	0.79				
slc16a9a	solute carrier family 16, member 9a	ENSDARG00000013926	1.22	2.42	0.93	3.17				
slc39a6	solute carrier family 39 (zinc transporter),	ENSDARG00000068143	1.20	0.76	0.73	1.25				
slc15a4	solute carrier family 15 (oligopeptide	ENSDARG00000102612	1.19	0.78	1.06	0.87				
slc13a5a	info solute carrier family 13 (sodium-dependent	ENSDARG00000077691	1.17	0.16	1.02	0.18				
slc40a1	solute carrier family 40 (iron-regulated	ENSDARG00000000241	1.16	1.10	1.34	0.95				

Fold change
>5.0 = red
>3.0 = light red
>1.2 = pale red
<0.85 = pale blue
<0.65 = light blue
<0.5 = blue

Gene counts
>1
>10
>100
>1000

p<0.05 p<0.1

Gene Symbol	Gene Title	ensemble	(1) DMSO (operated vs unoperated)	(2) Operated (SB431542 vs DMSO)	(3) Unoperated (SB431542 vs DMSO)	(4) SB431542 (operated vs unoperated)
slc20a1b	solute carrier family 20 (phosphate transporter),	ENSDARG00000010641	1.16	1.37	1.29	1.24
slc9a7	solute carrier family 9, subfamily A (NHE7,	ENSDARG00000076754	1.15	0.57	0.82	0.80
slc25a40	solute carrier family 25 member 40	ENSDARG00000015856	1.15	2.26	2.27	1.14
slc4a1a	solute carrier family 4 (anion exchanger),	ENSDARG00000012881	1.14	0.75	1.05	0.81
slc12a7a	solute carrier family 12 (potassium/chloride	ENSDARG00000073756	1.13	1.21	1.18	1.15
slc48a1b	solute carrier family 48 (heme transporter),	ENSDARG00000026109	1.11	1.29	2.08	0.69
slc16a9b	solute carrier family 16, member 9b	ENSDARG00000104687	1.11	1.64	2.21	0.82
slc25a20	solute carrier family 25 (carnitine/acylcarnitine	ENSDARG00000040401	1.09	0.74	0.84	0.97
slc12a7b	solute carrier family 12 (potassium/chloride	ENSDARG00000062058	1.09	0.97	1.19	0.89
slc16a6b	solute carrier family 16, member 6b	ENSDARG00000060246	1.07	1.48	1.30	1.22
slc5a6b	solute carrier family 5 (sodium/multivitamin and	ENSDARG00000014599	1.06	1.24	1.37	0.96
slc6a9	solute carrier family 6 (neurotransmitter	ENSDARG00000018534	1.06	1.03	1.12	0.97
slc43a2b	solute carrier family 43 (amino acid system L	ENSDARG00000061120	1.04	1.04	1.28	0.84
slc30a5	solute carrier family 30 (zinc transporter),	ENSDARG00000051921	1.03	1.20	1.31	0.95
slc2a15b	solute carrier family 2 (facilitated glucose	ENSDARG00000053269	1.03	1.60	3.19	0.52
slc35b4	solute carrier family 35, member B4	ENSDARG00000071087	1.03	1.00	0.93	1.12
slc16a1b	solute carrier family 16 (monocarboxylate	ENSDARG00000068572	1.02	0.56	0.68	0.84
slc35c2	solute carrier family 35 (GDP-fucose	ENSDARG00000037517	1.02	1.14	1.27	0.92
slc25a48	solute carrier family 25, member 48	ENSDARG00000021250	1.01	0.56	0.83	0.68
slc27a1a	solute carrier family 27 (fatty acid transporter),	ENSDARG00000006240	1.01	1.15	1.58	0.73
slc25a43	solute carrier family 25, member 43	ENSDARG00000102048	1.00	1.62	1.76	0.92
slc39a5	solute carrier family 39 (zinc transporter),	ENSDARG00000079525	1.00	2.31	1.00	1.70
slc25a26	solute carrier family 25 (S-adenosylmethionine	ENSDARG00000058208	1.00	0.92	0.93	0.99
slc25a55a	solute carrier family 25 (mitochondrial carrier:	ENSDARG00000020893	0.99	0.86	0.88	0.97
slc4a2a	solute carrier family 4 (anion exchanger),	ENSDARG00000028173	0.99	0.74	1.05	0.69
slc25a17	solute carrier family 25 (mitochondrial carrier;	ENSDARG00000061684	0.98	0.82	0.61	1.31
slc3a2a	solute carrier family 3 (amino acid transporter	ENSDARG00000036427	0.97	1.10	1.26	0.85
slc30a9	solute carrier family 30 (zinc transporter),	ENSDARG00000057272	0.96	1.20	1.28	0.90
slc37a4a	solute carrier family 37 (glucose-6-phosphate	ENSDARG00000038106	0.96	0.82	0.96	0.82
slc26a5	solute carrier family 26 (anion exchanger),	ENSDARG00000022424	0.95	1.21	1.62	0.71
slc16a10	solute carrier family 16 member 10	ENSDARG00000020984	0.95	0.63	0.85	0.70
slc25a44b	solute carrier family 25, member 44 b	ENSDARG00000035905	0.94	0.95	0.92	0.98
slc25a1a	solute carrier family 25 (mitochondrial carrier;	ENSDARG00000057110	0.94	1.10	1.23	0.84
slc6a1b	solute carrier family 6 (neurotransmitter	ENSDARG00000039647	0.93	0.78	0.74	0.98
slc1a4	solute carrier family 1 (glutamate/neutral amino	ENSDARG00000000551	0.90	0.68	0.98	0.63
slc22a2	solute carrier family 22 (organic cation	ENSDARG00000030530	0.89	1.56	1.66	0.84
slc25a38b	solute carrier family 25, member 38b	ENSDARG00000074533	0.88	0.59	0.73	0.71
slc6a19b	solute carrier family 6 (neutral amino acid	ENSDARG00000056719	0.87	3.33	0.32	9.20
slc1a2b	solute carrier family 1 (glial high affinity	ENSDARG00000102453	0.87	0.80	0.94	0.75
slc43a1a	solute carrier family 43 (amino acid system L	ENSDARG00000037393	0.85	0.70	0.76	0.79
slc35e1	solute carrier family 35, member E1	ENSDARG00000011945	0.85	0.99	0.86	0.98
slc29a2	solute carrier family 29 (equilibrative nucleoside	ENSDARG00000001767	0.85	1.24	1.21	0.87
slc7a6os	solute carrier family 7, member 6 opposite	ENSDARG00000010596	0.83	1.06	0.81	1.09
slc25a32a	solute carrier family 25 (mitochondrial folate	ENSDARG00000089791	0.82	1.09	1.01	0.88
slc24a4a	solute carrier family 24	ENSDARG00000015425	0.82	1.55	1.14	1.11
slc25a14	solute carrier family 25 (mitochondrial carrier,	ENSDARG00000026680	0.81	1.13	0.95	0.97
slc25a28	solute carrier family 25 (mitochondrial iron	ENSDARG00000052994	0.80	1.22	1.09	0.90
slc25a39	solute carrier family 25, member 39	ENSDARG00000007449	0.80	1.03	1.45	0.57
slc39a14	solute carrier family 39 (zinc transporter),	ENSDARG00000102387	0.79	1.03	0.35	2.36
slc6a17	solute carrier family 6 (neutral amino acid	ENSDARG00000068787	0.79	1.01	0.90	0.88
slc37a2	solute carrier family 37 (glucose-6-phosphate	ENSDARG00000023394	0.79	0.97	0.76	1.01
slc32a1	solute carrier family 32 (GABA vesicular	ENSDARG00000059775	0.79	1.00	0.81	0.97
slc20a2	solute carrier family 20 (phosphate transporter),	ENSDARG00000060796	0.79	1.27	1.02	0.98

DMSO unoperated COUNT | DMSO operated COUNT | SB43 unoperated COUNT | SB43 operated COUNT

Fold change
>5.0 = red
>3.0 = light red
>1.2 = pale red
<0.85 = pale blue
<0.65 = light blue
<0.5 = blue

p<0.05 p<0.1

Gene counts
>1
>10
>100
>1000

Gene Symbol	Gene Title	ensemble	(1) DMSO (operated vs unoperated)	(2) Operated (SB431542 vs DMSO)	(3) Unoperated (SB431542 vs DMSO)	(4) SB431542 (operated vs unoperated)	DMSO unoperated COUNT	DMSO operated COUNT	SB43 unoperated COUNT	SB43 operated COUNT
slc22a7b.2	solute carrier family 22 member 7-like	ENSDARG00000091252	0.78	0.68	0.24	2.27				
slc27a2a	solute carrier family 27 (fatty acid transporter),	ENSDARG00000036237	0.78	0.53	1.11	0.37				
slc7a6	solute carrier family 7 member 6	ENSDARG00000054423	0.78	1.20	1.60	0.59				
slc7a8a	solute carrier family 7 (cationic amino acid	ENSDARG00000075831	0.78	1.33	1.45	0.71				
slc25a12	solute carrier family 25 (aspartate/glutamate	ENSDARG00000102362	0.77	0.97	1.26	0.59				
slc25a11	solute carrier family 25 (mitochondrial carrier;	ENSDARG00000035741	0.77	0.86	0.90	0.73				
slc48a1a	solute carrier family 48 (heme transporter),	ENSDARG00000026907	0.75	2.03	1.54	1.00				
slc25a27	solute carrier family 25, member 27	ENSDARG00000042873	0.75	1.28	1.32	0.73				
slc25a5	solute carrier family 25 (mitochondrial carrier;	ENSDARG00000092553	0.75	1.30	1.16	0.84				
slc7a3a	solute carrier family 7 (cationic amino acid	ENSDARG00000020645	0.75	2.39	2.02	0.88				
slc2a3a	solute carrier family 2 (facilitated glucose	ENSDARG00000013295	0.75	1.39	1.56	0.67				
slc2a2	solute carrier family 2 (facilitated glucose	ENSDARG00000056196	0.72	1.19	0.98	0.87				
slc6a11a	solute carrier family 6 (neurotransmitter	ENSDARG00000074002	0.72	9.84	7.75	0.92				
slc1a3a	solute carrier family 1 (glial high affinity	ENSDARG00000104431	0.71	1.09	1.06	0.73				
slc38a5b	solute carrier family 38, member 5b	ENSDARG00000014587	0.71	0.81	0.78	0.74				
slc16a3	solute carrier family 16 (monocarboxylate	ENSDARG00000045051	0.71	1.15	1.15	0.71				
slc25a3b	solute carrier family 25 (mitochondrial carrier;	ENSDARG00000025566	0.70	0.86	0.79	0.76				
slc25a25a	solute carrier family 25 (mitochondrial carrier;	ENSDARG00000010572	0.70	2.26	1.46	1.08				
slc18a3a	solute carrier family 18 (vesicular acetylcholine	ENSDARG00000006356	0.69	0.92	0.83	0.76				
slc25a55b	solute carrier family 25 member 55b	ENSDARG00000013749	0.68	0.74	0.83	0.60				
slc39a13	solute carrier family 39 (zinc transporter),	ENSDARG00000000442	0.67	0.75	1.01	0.50				
slc16a12b	solute carrier family 16, member 12b	ENSDARG00000089885	0.65	1.14	0.94	0.78				
slc38a4	solute carrier family 38, member 4	ENSDARG00000018149	0.65	0.94	0.94	0.64				
slc43a2a	solute carrier family 43 (amino acid system L	ENSDARG00000036848	0.64	1.97	0.55	2.30				
slc35a4	solute carrier family 35, member A4	ENSDARG00000062379	0.63	1.17	0.85	0.87				
slc38a6	solute carrier family 38, member 6	ENSDARG00000054312	0.63	3.23	1.87	1.08				
slc3a2b	solute carrier family 3 (amino acid transporter	ENSDARG00000037012	0.62	1.75	0.71	1.53				
slc9a8	solute carrier family 9, subfamily A (NHE8,	ENSDARG00000020699	0.62	1.19	0.98	0.76				
slc37a4b	solute carrier family 37 (glucose-6-phosphate	ENSDARG00000077180	0.59	0.99	0.80	0.74				
slc6a19a.1	solute carrier family 6 (neutral amino acid	ENSDARG00000018621	0.59	2.23	1.79	0.74				
slc44a5b	solute carrier family 44 member 5b	ENSDARG00000057419	0.58	1.89	0.72	1.53				
slc25a51b	solute carrier family 25 member 51-like	ENSDARG00000040463	0.58	1.05	0.82	0.75				
slc25a36b	solute carrier family 25 member 36b	ENSDARG00000015915	0.57	1.18	0.86	0.78				
slc25a4	solute carrier family 25 (mitochondrial carrier;	ENSDARG00000027355	0.54	0.97	0.85	0.61				
slc16a4	solute carrier family 16 member 4	ENSDARG00000042807	0.52	1.29	0.75	0.89				
slc8a4b	solute carrier family 8 (sodium/calcium	ENSDARG00000037145	0.49	1.49	0.63	1.16				
slc2a11l	solute carrier family 2 (facilitated glucose	ENSDARG00000062873	0.44	0.21	0.33	0.28				
slc13a1	solute carrier family 13 (sodium/sulphate	ENSDARG00000045638	0.40	1.53	0.36	1.68				
slc5a12	solute carrier family 5	ENSDARG00000005004	0.40	6.00	1.29	1.85				
slc25a3a	solute carrier family 25 (mitochondrial carrier;	ENSDARG00000027424	0.35	2.76	0.69	1.38				
slc25a44a	solute carrier family 25, member 44 a	ENSDARG00000045927	0.16	3.92	0.62	1.03				
slc25a37	solute carrier family 25 (mitochondrial iron	ENSDARG00000073743	0.12	2.31	0.50	0.54				
tgm2l	transglutaminase 2, like	ENSDARG00000093381	7.27	0.78	2.09	2.70				
tgm2b	transglutaminase 2b	ENSDARG00000074094	4.20	0.66	3.23	0.86				
tgm2a	transglutaminase 2, C polypeptide A	ENSDARG00000070157	0.34	0.79	1.19	0.22				
tgm1l4	transglutaminase 1 like 4	ENSDARG00000101407	0.27	0.68	0.25	0.74				
tnnt2a	troponin T type 2a (cardiac)	ENSDARG00000020610	4.17	0.21	1.00	1.00				
tnnt2c	troponin T2c, cardiac	ENSDARG00000032242	3.90	0.73	1.01	2.82				
tnni2b.1	troponin I type 2b (skeletal, fast), tandem	ENSDARG00000035958	2.34	0.97	1.35	1.68				
tnni1b	troponin I type 1b (skeletal, slow)	ENSDARG00000052708	1.94	0.59	0.62	1.86				
tnnc1a	troponin C type 1a (slow)	ENSDARG00000011400	0.70	1.16	0.12	6.67				

Fold change
>5.0 = red
>3.0 = light red
>1.2 = pale red
<0.85 = pale blue
<0.65 = light blue
<0.5 = blue

Gene counts
>1
>10
>100
>1000

p<0.05 p<0.1

Gene Symbol	Gene Title	ensemble	(1) DMSO (operated vs unoperated)	(2) Operated (SB431542 vs DMSO)	(3) Unoperated (SB431542 vs DMSO)	(4) SB431542 (operated vs unoperated)	DMSO unoperated COUNT	DMSO operated COUNT	SB43 unoperated COUNT	SB43 operated COUNT
tnnc1b	troponin C type 1b (slow)	ENSDARG00000037539	0.65	0.90	0.84	0.69				
tnni4b.2	troponin I4b, tandem duplicate 2	ENSDARG00000036671	0.65	1.14	1.01	0.74				
tnni1d	troponin I, skeletal, slow d	ENSDARG00000073766	0.62	0.89	0.97	0.57				
tnnt3a	troponin T type 3a (skeletal, fast)	ENSDARG00000030270	0.60	0.91	0.77	0.71				
tnnc2	troponin C type 2 (fast)	ENSDARG00000070835	0.56	1.22	0.99	0.69				
tnni2a.4	troponin I type 2a (skeletal, fast), tandem	ENSDARG00000029069	0.56	1.12	0.91	0.68				
tnnt3b	troponin T type 3b (skeletal, fast)	ENSDARG00000068457	0.55	1.02	0.92	0.61				
tnni2a.3	troponin I type 2a (skeletal, fast), tandem	ENSDARG00000013752	0.48	1.29	0.70	0.89				
tnnt2d	troponin T2d, cardiac	ENSDARG00000002988	0.43	0.98	0.84	0.51				
tnni1c	troponin I, skeletal, slow c	ENSDARG00000042559	0.32	0.74	0.60	0.39				
tnni2b.2	troponin I type 2b (skeletal, fast), tandem	ENSDARG00000029995	0.31	1.63	0.89	0.58				
tnni4b.1	troponin I4b, tandem duplicate 1	ENSDARG00000092999	0.25	2.11	0.60	0.87				
tubb6	tubulin, beta 6 class V	ENSDARG00000104801	5.22	0.64	1.20	2.78				
tuba1b	tubulin, alpha 1b	ENSDARG00000045367	2.64	0.73	0.71	2.68				
tubd1	tubulin, delta 1	ENSDARG00000058219	2.30	0.68	1.07	1.45				
tuba8l	tubulin, alpha 8 like	ENSDARG00000042708	1.86	0.67	0.69	1.81				
tuba8l4	tubulin, alpha 8 like 4	ENSDARG00000006260	1.58	0.75	0.83	1.43				
tubb4b	tubulin, beta 4B class IVb	ENSDARG00000002344	1.37	0.97	1.24	1.08				
tubb4b	tubulin, beta 4B class IVb	ENSDARG00000002344	1.37	0.97	1.24	1.08				
tubb2b	tubulin, beta 2b	ENSDARG00000098591	1.37	0.81	0.90	1.23				
tubg1	tubulin, gamma 1	ENSDARG00000015610	1.31	0.61	0.97	0.82				
tubgcp4	tubulin, gamma complex associated protein 4	ENSDARG00000005374	1.30	0.82	0.92	1.15				
tubgcp5	tubulin, gamma complex associated protein 5	ENSDARG00000077442	1.28	0.88	1.21	0.93				
tubgcp2	tubulin, gamma complex associated protein 2	ENSDARG00000013079	1.25	1.14	1.43	1.00				
tuba4l	tubulin, alpha 4 like	ENSDARG00000074289	1.22	1.09	0.93	1.43				
tuba7l	tubulin, alpha 7 like	ENSDARG00000104643	1.00	1.00	3.04	0.28				
tuba8l3	tubulin, alpha 8 like 3	ENSDARG00000070155	0.93	0.78	0.83	0.87				
tubb5	tubulin, beta 5	ENSDARG00000037997	0.91	1.67	1.31	1.15				
tubgcp3	tubulin, gamma complex associated protein 3	ENSDARG00000029133	0.89	2.13	2.08	0.91				
tuba2	tubulin, alpha 2	ENSDARG00000045014	0.79	1.11	1.29	0.68				
tubb2	tubulin, beta 2A class IIa	ENSDARG00000039522	0.70	1.20	1.09	0.77				
tuba1a	tubulin, alpha 1a /// zgc:123298	ENSDARG00000001889	0.67	0.89	0.79	0.75				
tuba1c	tubulin, alpha 1c	ENSDARG00000055216	0.59	1.00	0.92	0.64				
tuba8l2	tubulin, alpha 8 like 2	ENSDARG00000031164	0.45	1.17	0.94	0.56				
ube2c	ubiquitin-conjugating enzyme E2C	ENSDARG00000114670	2.33	0.88	0.97	2.11				
ube2d1b	ubiquitin-conjugating enzyme E2D 1b	ENSDARG00000038576	2.10	1.45	1.93	1.58				
ube2d2l	ubiquitin-conjugating enzyme E2D 2 (UBC4/5	ENSDARG00000099749	1.72	0.70	0.95	1.27				
ube3d	ubiquitin protein ligase E3D	ENSDARG00000026178	1.69	0.88	0.94	1.58				
ube2q2	ubiquitin-conjugating enzyme E2Q family	ENSDARG00000013990	1.60	2.18	2.16	1.62				
ube2d1a	ubiquitin-conjugating enzyme E2D 1a	ENSDARG00000029107	1.56	1.86	2.85	1.02				
ube2s	ubiquitin-conjugating enzyme E2S	ENSDARG00000031775	1.46	0.93	0.98	1.38				
ube2v2	ubiquitin-conjugating enzyme E2 variant 2	ENSDARG00000028198	1.36	1.02	0.84	1.64				
ube2l3b	ubiquitin-conjugating enzyme E2L 3b	ENSDARG00000027141	1.36	0.86	1.01	1.15				
ube4b	ubiquitination factor E4B, UFD2 homolog (S.	ENSDARG00000037017	1.29	0.76	0.90	1.09				
ube2na	ubiquitin-conjugating enzyme E2Na	ENSDARG00000008748	1.28	1.12	1.16	1.23				
ube2ia	ubiquitin-conjugating enzyme E2Ia	ENSDARG00000052649	1.25	1.17	1.47	0.99				
ube2r2	ubiquitin-conjugating enzyme E2R 2	ENSDARG00000058740	1.24	1.19	1.28	1.16				
ube2d2	ubiquitin-conjugating enzyme E2D 2 (UBC4/5	ENSDARG00000043484	1.24	0.96	0.91	1.30				
ube2g2	ubiquitin-conjugating enzyme E2G 2 (UBC7	ENSDARG00000025404	1.18	1.68	2.18	0.91				
ube2ib	ubiquitin-conjugating enzyme E2Ib	ENSDARG00000007438	1.15	0.72	0.78	1.06				
ube2kb	ubiquitin-conjugating enzyme E2Kb (UBC1	ENSDARG00000013505	1.11	0.87	0.76	1.28				

Fold change
- >5.0 = red
- >3.0 = light red
- >1.2 = pale red
- <0.85 = pale blue
- <0.65 = light blue
- <0.5 = blue

Gene counts
- >1
- >10
- >100
- >1000

p<0.05 p<0.1

Gene Symbol	Gene Title	ensemble	(1) DMSO (operated vs unoperated)	(2) Operated (SB431542 vs DMSO)	(3) Unoperated (SB431542 vs DMSO)	(4) SB431542 (operated vs unoperated)	DMSO unoperated COUNT	DMSO operated COUNT	SB43 unoperated COUNT	SB43 operated COUNT
ube2g1a	ubiquitin-conjugating enzyme E2G 1a (UBC7	ENSDARG00000015292	1.09	1.01	1.23	0.89				
ube2e3	ubiquitin-conjugating enzyme E2E 3 (UBC4/5	ENSDARG00000012244	1.07	1.02	1.13	0.97				
ube2ql1	ubiquitin-conjugating enzyme E2Q family-like 1	ENSDARG00000079276	1.05	1.56	0.82	2.00				
ube2e2	ubiquitin-conjugating enzyme E2E 2	ENSDARG00000034670	1.04	1.11	1.27	0.91				
ube2q1	ubiquitin-conjugating enzyme E2Q family	ENSDARG00000100766	0.98	0.83	0.75	1.08				
ube2a	ubiquitin-conjugating enzyme E2A (RAD6	ENSDARG00000098466	0.95	0.97	1.15	0.80				
ube2nb	ubiquitin-conjugating enzyme E2Nb	ENSDARG00000045877	0.87	0.91	0.91	0.87				
ube2h	ubiquitin-conjugating enzyme E2H (UBC8	ENSDARG00000000019	0.80	0.86	0.98	0.71				
ube2d3	ubiquitin-conjugating enzyme E2D 3	ENSDARG00000038473	0.79	1.18	0.92	1.00				
ube2g1b	ubiquitin-conjugating enzyme E2G 1b (UBC7	ENSDARG00000069527	0.69	1.15	0.95	0.83				
ube2d4	ubiquitin-conjugating enzyme E2D 4	ENSDARG00000015057	0.38	0.95	0.37	0.98				
ywhabb	tyrosine 3-monooxygenase/tryptophan 5-	ENSDARG00000075758	2.08	1.13	1.36	1.72				
ywhaqb	tyrosine 3-monooxygenase/tryptophan 5-	ENSDARG00000023323	1.85	1.54	1.26	2.27				
ywhaba	tyrosine 3-monooxygenase/tryptophan 5-	ENSDARG00000013078	1.57	0.98	1.15	1.33				
ywhaqa	tyrosine 3-monooxygenase/tryptophan 5-	ENSDARG00000042539	1.50	0.81	0.94	1.30				
ywhaz	tyrosine 3-monooxygenase/tryptophan 5-	ENSDARG00000032575	1.49	0.95	0.71	2.00				
ywhae2	tyrosine 3-monooxygenase/tryptophan 5-	ENSDARG00000017014	1.25	0.92	0.93	1.24				
ywhah	tyrosine 3-monooxygenase/tryptophan 5-	ENSDARG00000005560	0.93	0.75	0.81	0.87				
ywhae1	tyrosine 3-monooxygenase/tryptophan 5-	ENSDARG00000006399	0.82	0.87	0.67	1.07				
ywhabl	tyrosine 3-monooxygenase/tryptophan 5-	ENSDARG00000040287	0.81	1.07	0.80	1.09				

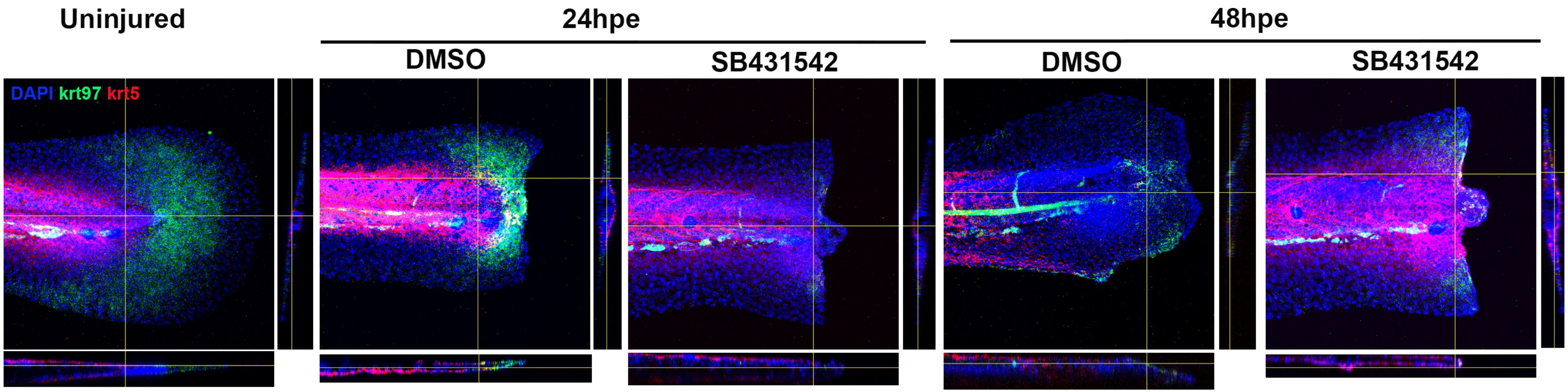

Supplemental Figure 6: Images from Figure 5a showing orthogonal views. Yellow lines indicate the plane shown in the orthogonal views.

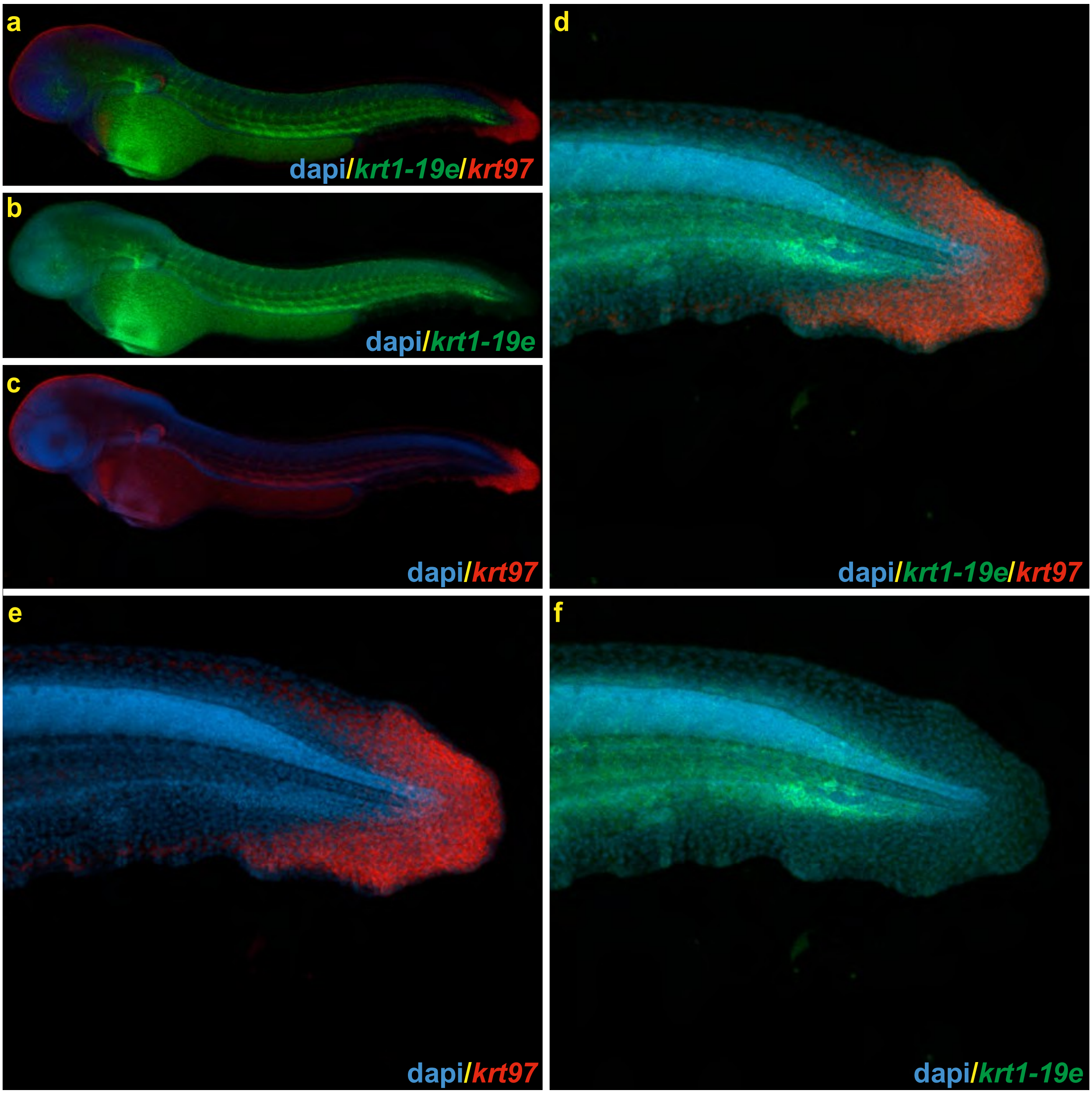

Supplemental Figure 7: *krt1-19e* is expressed in the trunk region and does not overlap extensively with *krt97* at 48hpf. (a-c) Low magnification and (d-f) high magnification images of *krt1-19e* expression is shown in green, DAPI staining of nuclei in blue and *krt97* in red. *krt1-19e* is expressed in the trunk and does not overlap with *krt97* at this stage.

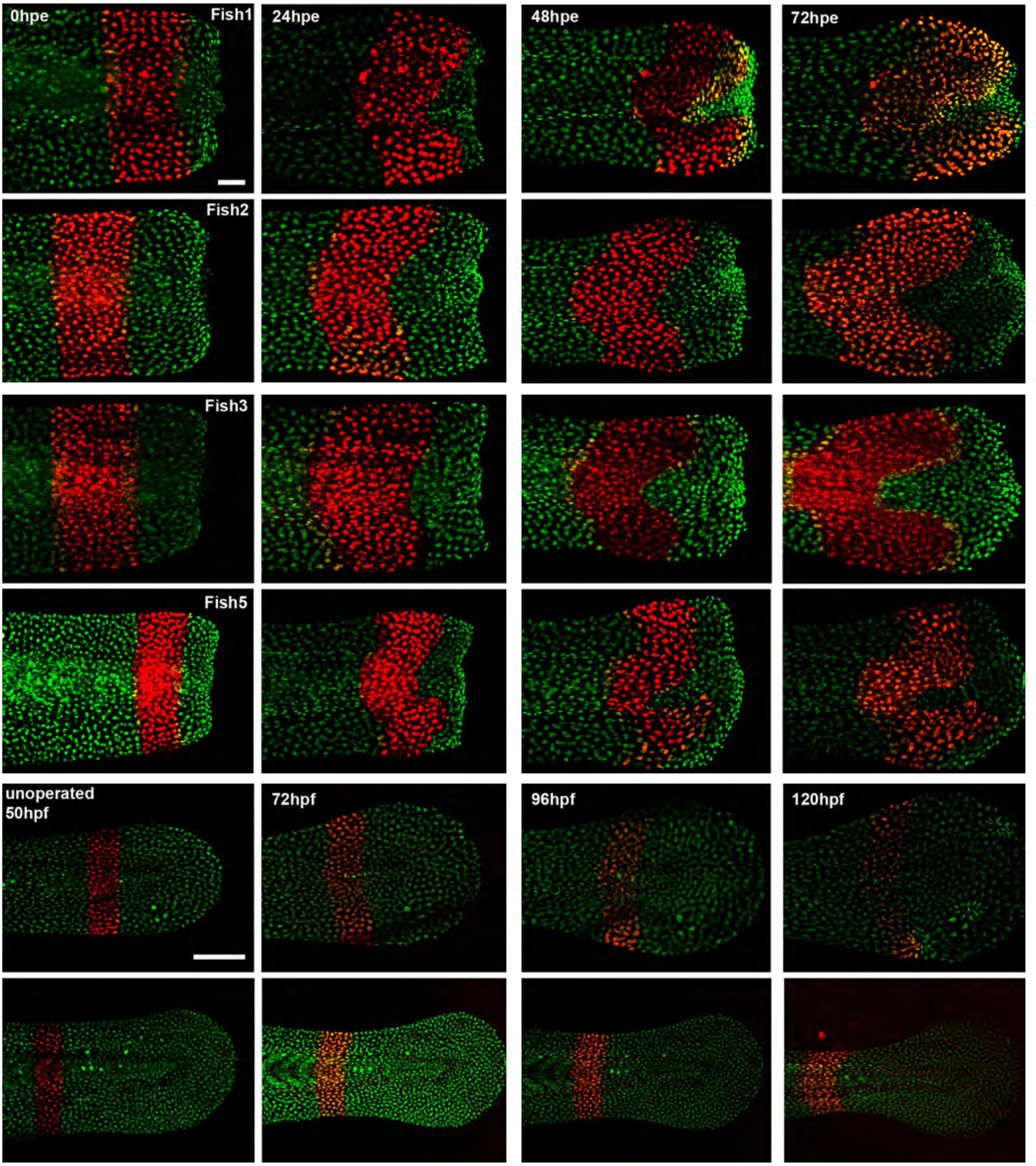

Supplemental Figure 8: Examples of photoconversion experiments during regeneration.
