# Supplementary material for: TGF-beta coordinates changes in Keratin gene expression during complex tissue regeneration
Source: Sci Rep. 2025 Nov 4;15:38645. doi: 10.1038/s41598-025-22590-2 (PMC12586446; doi:10.1038/s41598-025-22590-2)
Supplement: Supplementary file 2 — Supplementary Information 2. [file 41598_2025_22590_MOESM2_ESM.pdf]

Supplemental Table 1. R packages. This table lists the packages and versions used for the scI

Package	Version	
abind	1.4-8	
askpass	1.2.0	
assertthat	0.2.1	
assorthead	0.99.8	
backports	1.5.0	
base64enc	0.1-3	
beachmat	2.21.6	
beeswarm	0.4.0	
BH	1.84.0-0	
Biobase	2.65.1	
BiocGenerics	0.51.1	
BiocManage	1.30.25	
BiocParallel	1.39.0	
BiocSingular	1.21.3	
BiocVersion	3.20.0	
bit	4.5.0	
bit64	4.5.2	
bitops	1.0-8	
blob	1.2.4	
brew	1.0-10	
brio	1.1.5	
broom	1.0.6	
bslib	0.8.0	
cachem	1.1.0	
Cairo	1.6-2	
callr	3.7.6	
car	3.1-2	
carData	3.0-5	
caTools	1.18.3	
cellranger	1.1.0	
circlize	0.4.16	
cli	3.6.3	
clipr	0.8.0	
clock	0.7.1	
clue	0.3-65	
colorspace	2.1-1	
commonmar	1.9.1	
ComplexHea	2.21.0	
conflicted	1.2.0	
corrplot		0.94
COTAN	2.5.7	

cowplot	1.1.3
cpp11	0.5.0
crayon	1.5.3
credentials	2.0.1
crosstalk	1.2.1
curl	5.2.3
data.table	1.16.0
DBI	1.2.3
dbplyr	2.5.0
DelayedArray	0.31.11
DelayedMatr	1.27.3
deldir	2.0-4
dendextend	1.17.1
Deriv	4.1.6
desc	1.4.3
DESeq2	1.45.3
devtools	2.4.5
diagram	1.6.5
dials	1.3.0
DiceDesign	1.1
diffobj	0.3.5
digest	0.6.37
doBy	4.6.22
doFuture	1.0.1
doParallel	1.0.17
dotCall64	1.1-1
downlit	0.4.4
dplyr	1.1.4
dqrng	0.4.1
DropletUtils	1.25.2
dtplyr	1.3.1
edgeR	4.3.15
ellipsis	0.3.2
evaluate	1.0.0
fansi	1.0.6
farver	2.1.2
fastDummies	1.7.4
fastmap	1.2.0
fitdistrplus	1.2-1
FNN	1.1.4.1
fontawesom	0.5.2
forcats	1.0.0
foreach	1.5.2
formatR	1.14
fs	1.6.4
furrr	0.3.1

futile.logger	1.4.3
futile.options	1.0.1
future	1.34.0
future.apply	1.11.2
gargle	1.5.2
generics	0.1.3
GenomeInfo	1.41.1
GenomeInfo	1.2.12
GenomicRan	1.57.1
gert	2.1.2
GetoptLong	1.0.5
ggbeeswarm	0.7.2
ggplot2	3.4.4
ggplotify	0.1.2
ggprism	1.0.5
ggpubr	0.6.0
ggrastr	1.0.2
ggrepel	0.9.6
ggridges	0.5.6
ggsci	3.2.0
ggsignif	0.6.4
ggthemes	5.1.0
gh	1.4.1
gitcreds	0.1.2
GlobalOption	0.1.2
globals	0.16.3
glue	1.7.0
goftest	1.2-3
googledrive	2.1.1
googlesheets	1.1.1
gower	1.0.1
GPfit	1.0-8
gplots	3.1.3.1
gridExtra	2.3
gridGraphics	0.5-1
gtable	0.3.5
gtools	3.9.5
hardhat	1.4.0
haven	2.5.4
HDF5Array	1.33.6
hdf5r	1.3.11
here	1.0.1
highr	0.11
hms	1.1.3
htmltools	0.5.8.1
htmlwidgets	1.6.4

httpuv	1.6.15
httr	1.4.7
httr2	1.0.4
ica	1.0-3
ids	1.0.1
igraph	2.0.3
ini	0.3.1
ipred	0.9-15
IRanges	2.39.2
irlba	2.3.5.1
isoband	0.2.7
iterators	1.0.14
janitor	2.2.0
jquerylib	0.1.4
jsonlite	1.8.9
kernlab	0.9-33
knitr	1.48
ks	1.14.3
labeling	0.4.3
lambda.r	1.2.4
later	1.3.2
latex2exp	0.9.6
lava	1.8.0
lazyeval	0.2.2
leiden	0.4.3.1
lhs	1.2.0
lifecycle	1.0.4
limma	3.61.9
listenv	0.9.1
lme4	1.1-35.5
lmtest	0.9-40
locfit	1.5-9.10
lubridate	1.9.3
magrittr	2.0.3
markdown	1.13
MatrixGener	1.17.0
MatrixModel	0.5-3
matrixStats	1.4.1
mclust	6.1.1
memoise	2.0.1
microbenchn	1.5.0
mime	0.12
miniUI	0.1.1.1
minqa	1.2.8
modelenv	0.1.1
modelr	0.1.11

multicool	1.0.1
munsell	0.5.1
mvtnorm	1.3-1
Nebulosa	1.15.0
nloptr	2.1.1
numDeriv	2016.8-1.1
openssl	2.2.2
openxlsx	4.2.7.1
paletteer	1.6.0
parallelDist	0.2.6
parallelly	1.38.0
parsnip	1.2.1
patchwork	1.3.0
pbapply	1.7-2
pbkrtest	0.5.3
PCAtools	2.17.0
pillar	1.9.0
pkgbuild	1.4.4
pkgconfig	2.0.3
pkgdown	2.1.1
pkgload	1.4.0
plotly	4.10.4
plyr	1.8.9
png	0.1-8
polyclip	1.10-7
polynom	1.4-1
pracma	2.4.4
praise	1.0.0
presto	1.0.0
prettyunits	1.2.0
prismatic	1.1.2
processx	3.8.4
prodlim	2024.06.25
profvis	0.4.0
progress	1.2.3
progressr	0.14.0
promises	1.3.0
ps	1.8.0
purrr	1.0.2
quantreg	5.98
R.methodsS3	1.8.2
R.oo	1.26.0
R.utils	2.12.3
R6	2.5.1
ragg	1.3.3
RANN	2.6.2

rappdirs	0.3.3	
rcmdcheck	1.4.0	
RColorBrewe	1.1-3	
Rcpp	1.0.13	
RcppAnnoy	0.0.22	
RcppArmadil	14.0.2-1	
RcppEigen	0.3.4.0.2	
RcppGSL	0.3.13	
RcppHNSW	0.6.0	
RcppParallel	5.1.9	
RcppProgres	0.4.2	
RcppTOML	0.2.2	
RcppZiggura	0.1.6	
RCurl	1.98-1.16	
readr	2.1.5	
readxl	1.4.3	
recipes	1.1.0	
rematch	2.0.0	
rematch2	2.1.2	
remotes	2.5.0	
reprex	2.1.1	
reshape2	1.4.4	
reticulate	1.39.0	
Rfast	2.1.0	
rhdf5	2.49.0	
rhdf5filters	1.17.0	
Rhdf5lib	1.27.0	
rjson	0.2.23	
rlang	1.1.4	
rmarkdown		2.28
ROCR	1.0-11	
roxygen2	7.3.2	
rprojroot	2.0.4	
rsample	1.2.1	
RSpectra	0.16-2	
rstatix	0.7.2	
rstudioapi	0.16.0	
rsvd	1.0.5	
Rtsne		0.17
rversions	2.1.2	
rvest	1.0.4	
S4Arrays	1.5.7	
S4Vectors	0.43.2	
sass	0.4.9	
ScaledMatri	1.13.0	
scales	1.3.0	

scattermore	1.2
scCustomize	2.1.2
SCpubr	2.0.2
sctransform	0.4.1
scuttle	1.15.4
selectr	0.4-2
sessioninfo	1.2.2
Seurat	5.1.0
SeuratData	0.2.2.9001
SeuratObject	5.0.2
SeuratWrapp	0.3.2
sfd	0.1.0
shape	1.4.6.1
shiny	1.9.1
SingleCellExp	1.27.2
sitmo	2.0.2
slider	0.3.1
snakecase	0.11.1
snow	0.4-4
sourcetools	0.1.7-1
sp	2.1-4
spam	2.10-0
SparseArray	1.5.37
SparseM	1.84-2
sparseMatrix	1.17.2
spatstat.data	3.1-2
spatstat.expl	3.3-2
spatstat.geor	3.3-3
spatstat.ranc	3.3-2
spatstat.spar	3.1-0
spatstat.univ	3.0-1
spatstat.utils	3.1-0
SQUAREM	2021.1
statmod	1.5.0
stringi	1.8.4
stringr	1.5.1
Summarized	1.35.1
sys	3.4.2
systemfonts	1.1.0
tensor	1.5
testthat	3.2.1.1
textshaping	0.4.0
tibble	3.2.1
tidyr	1.3.1
tidyselect	1.2.1
tidyverse	2.0.0

timechange	0.3.0
timeDate	4041.11
tinytex	0.53
tune	1.2.1
tzdb	0.4.0
UCSC.utils	1.1.0
umap	0.2.10.0
urlchecker	1.0.1
usethis	3.0.0
utf8	1.2.4
uuid	1.2-1
uwot	0.2.2
vctrs	0.6.5
VennDiagran	1.7.3
vipor	0.4.7
viridis	0.6.5
viridisLite	0.4.2
vroom	1.6.5
waldo	0.5.3
warp	0.2.1
whisker	0.4.1
withr	3.0.1
workflows	1.1.4
xfun	0.47
xml2	1.3.6
xopen	1.0.1
xtable	1.8-4
XVector	0.45.0
yaml	2.3.10
yardstick	1.3.1
yulab.utils	0.1.7
zeallot	0.1.0
zip	2.3.1
zlibbioc	1.51.1
zoo	1.8-12

RNA-seq analysis presented in the study.
